# σ-GeH and Germyl Cationic Pt(II) Complexes

**DOI:** 10.1021/acs.inorgchem.2c03186

**Published:** 2022-11-02

**Authors:** Carlos J. Laglera-Gándara, Pablo Ríos, Francisco José Fernández-de-Córdova, Marina Barturen, Israel Fernández, Salvador Conejero

**Affiliations:** † Instituto de Investigaciones Químicas (IIQ), Departamento de Química Inorgánica, Centro de Innovación en Química Avanzada (ORFEO-CINQA), CSIC and Universidad de Sevilla, Sevilla 41092, Spain; ‡ Departamento de Química Orgánica I y Centro de Innovación en Química Avanzada (ORFEO-CINQA), facultad de Químicas, Universidad Complutense de Madrid, Madrid 28040, Spain

## Abstract

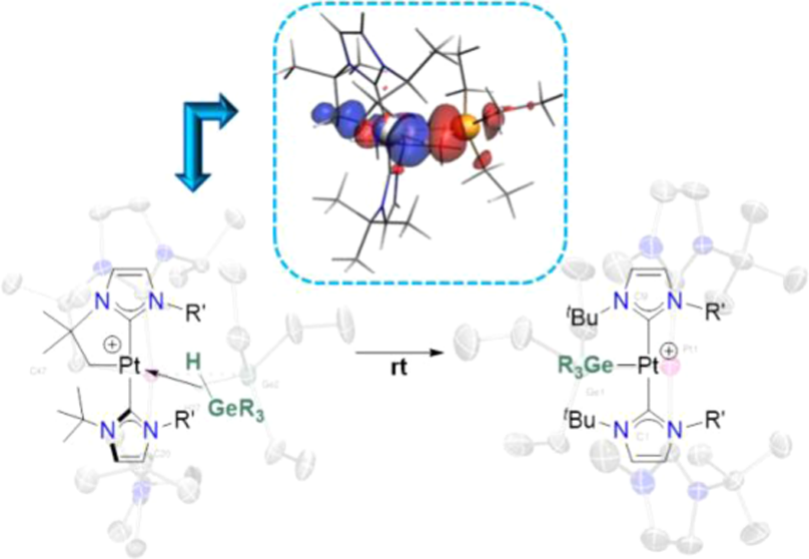

The low electron count Pt(II) complexes [Pt(NHC′)(NHC)][BAr^F^] (where NHC is a *N*-heterocyclic carbene ligand and NHC′ its metalated form) react with tertiary hydrogermanes HGeR_3_ at room temperature to generate the 14-electron platinum(II) germyl derivatives [Pt(GeR_3_)(NHC)_2_][BAr^F^]. Low-temperature NMR studies allowed us to detect and characterize spectroscopically some of the σ-GeH intermediates [Pt(η^2^-HGeR_3_)(NHC′)(NHC)][BAr^F^] that evolve into the platinum-germyl species. One of these compounds has been characterized by X-ray diffraction studies, and the interaction of the H–Ge bond with the platinum center has been analyzed in detail by computational methods, which suggest that the main contribution is the donation of the H–Ge to a σ*(Pt–C) orbital, but backdonation from the platinum to the σ*(Ge–H) orbital is significant. Primary and secondary hydrogermanes also produce the corresponding platinum-germyl complexes, a result that contrasts with the reactivity observed with primary silanes, in which carbon–silicon bond-forming reactions have been reported. According to density functional theory calculations, the formation of Pt–Ge/C–H bonds is both kinetically and thermodynamically preferred over the competitive reaction pathway leading to Pt–H/C–Ge bonds.

## Abstract 2

Cyclometalated Pt(II) complexes interact with hydrogermanes to form σ-GeH complexes. Theoretical calculations suggest that the GeH → Pt interactions fall between classical η^2^ and η^1^ coordination modes. These compounds are thermally unstable, evolving through activation of the Ge−H bond to form platinum-germyl species in a process that involves the formation of C−H/Pt−Ge bonds. DFT studies point to a σ-CAM mechanism in which the competitive reaction pathway leading to C−Ge/Pt−H bonds is both kinetically and thermodynamically less favored.

## Introduction

**Scheme 1:**
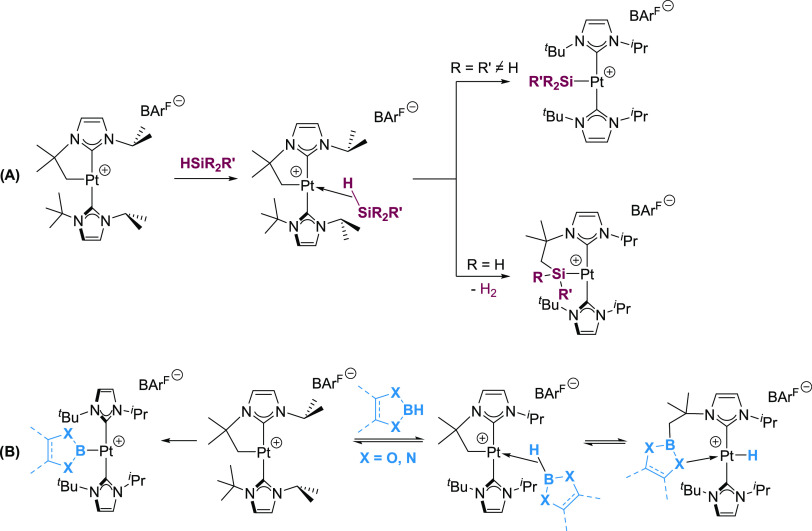
Reactions of Cyclometalated Complex [Pt(I^*t*^Bu^*i*^Pr′)(I^*t*^Bu^*i*^Pr)][BAr^F^] with Hydrosilanes (**A**) and Hydroboranes (**B**)

The interaction of E–H bonds (E = H, B, Si, Ge,···) with transition metals leading to σ-EH complexes is considered the first step toward the cleavage of the E–H bond to forge two new M–E and M–H bonds through a formal oxidative addition process. In the last decades, our understanding of this type of interaction has reached a high degree of knowledge, thanks to the spectroscopic observation, crystallographic isolation, and computational analyses of a great number of species of this type, particularly regarding those systems with dihydrogen and hydrosilanes, as well as with hydroboranes.^1^ Obviously, this has been particularly driven by the participation of this type of compounds in catalytic hydrogenation, hydrosilylation, and hydroboration reactions. On the contrary, the number of σ-GeH complexes spectroscopically observed is very limited,^2^ and only very few have been characterized by X-ray diffraction methods.^3^ Besides, all of these complexes are neutral derivatives, but, to the best of our knowledge, no cationic systems have been reported. This might be due, at least in part, to the highly reactive nature of cationic σ-GeH complexes (similar to their σ-SiH counterparts), which are prone to the heterolytic cleavage of the Ge–H bond even by weak nucleophiles.^1h^ In a series of recent contributions by our group, we have been able to isolate and characterize, by spectroscopic means and X-ray diffraction studies, σ-EH cationic platinum complexes of dihydrogen, silanes, and boranes.^4^ We have observed that these species are thermally unstable and evolve through E–H bond cleavage. Intriguingly, these processes result, in subsequent steps, in C–H/Pt–Si or C–Si/Pt–H coupling reactions for silanes (depending on the nature of the silane, Scheme 1A)^4b^ and in reversible C–B/Pt–H coupling events (finally leading to the thermodynamically more stable C–H/Pt–B species, Scheme 1B).^4a^ Considering all of the above and the increasing interest in the use of organo-germanium compounds,^5^ in this contribution, we have explored the reactivity of cationic Pt(II) complexes [Pt(NHC′)(NHC)][BAr^F^] toward hydrogermanes, which have led to the successful isolation of the corresponding σ-GeH derivatives.

## Results and Discussion

**Figure 1:**
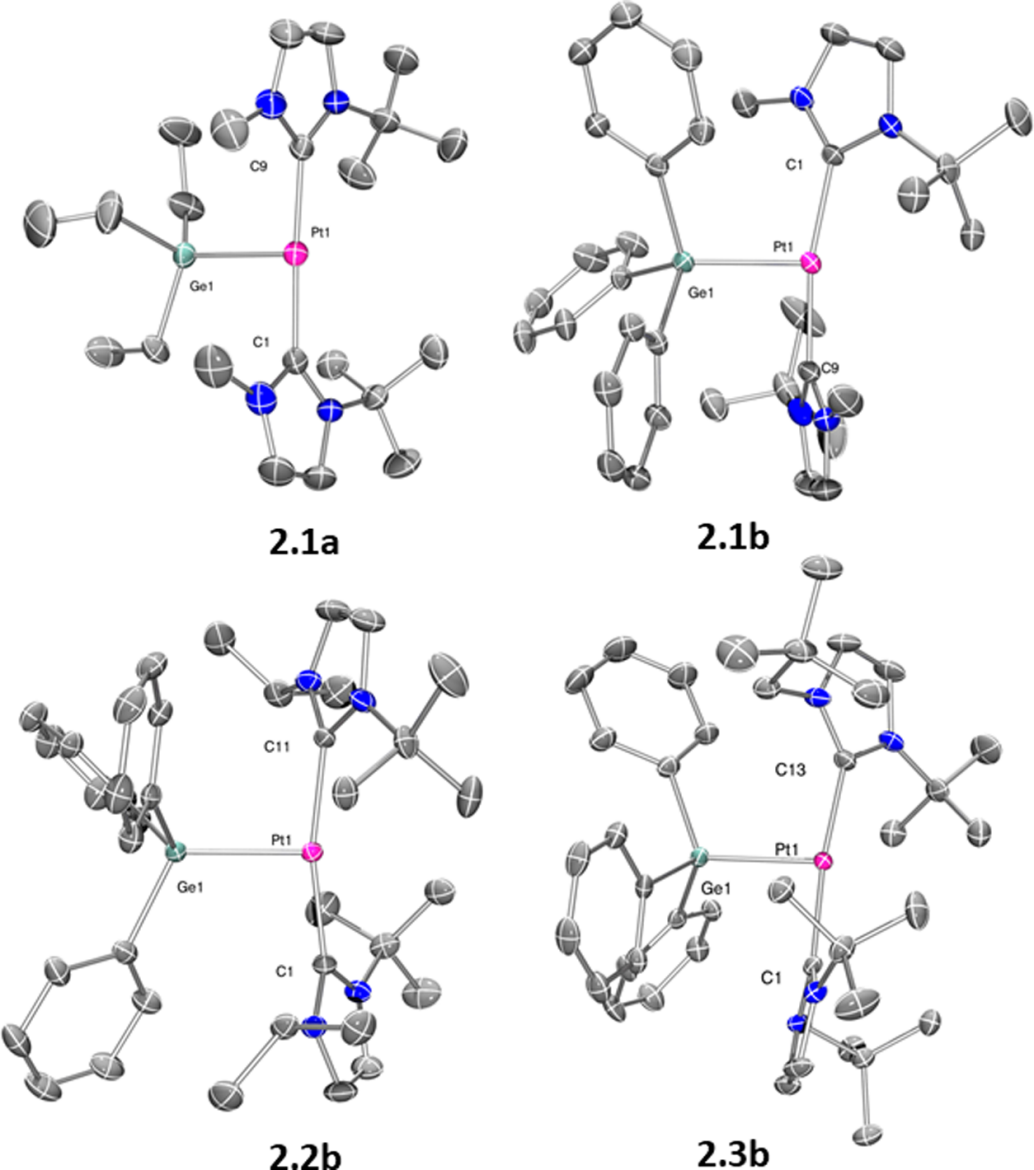
Thermal ellipsoid depiction of the cationic fragments of complexes **2.1a**, **2.1b**, **2.2b**, and **2.3b** (hydrogens omitted for clarity). Thermal ellipsoids at 30% probability. Selected bond distances (Å) and angles (°): **2.1a**: Pt1-Ge1, 2.3938(6); Pt1-C1, 2.029(6); Pt1-C9, 2.020(6); and C1-Pt1-C9, 172.1(2). **2.1b**: Pt1-Ge1, 2.3953(5); Pt1-C1, 2.031(3); Pt1-C9, 2.028(3); and C1-Pt1-C9, 169.0(1). **2.2b**: Pt1-Ge1, 2.3881(5); Pt1-C1, 2.015(3); Pt1-C11, 2.027(3); and C1-Pt1-C11, 167.5(1). **2.3b**: Pt1-Ge1, 2.4075(6); Pt1-C1, 2.029(4); Pt1-C13, 2.041(4); and C1-Pt1-C9, 172.8(2).

**Scheme 2:**
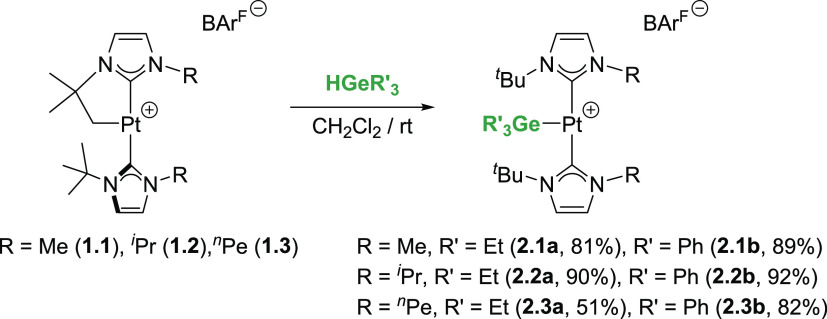
Reaction of Complexes [Pt(NHC′)(NHC)][BAr^F^] (**1.1**–**1.3**) with Tertiary Hydrogermanes

**Scheme 3:**
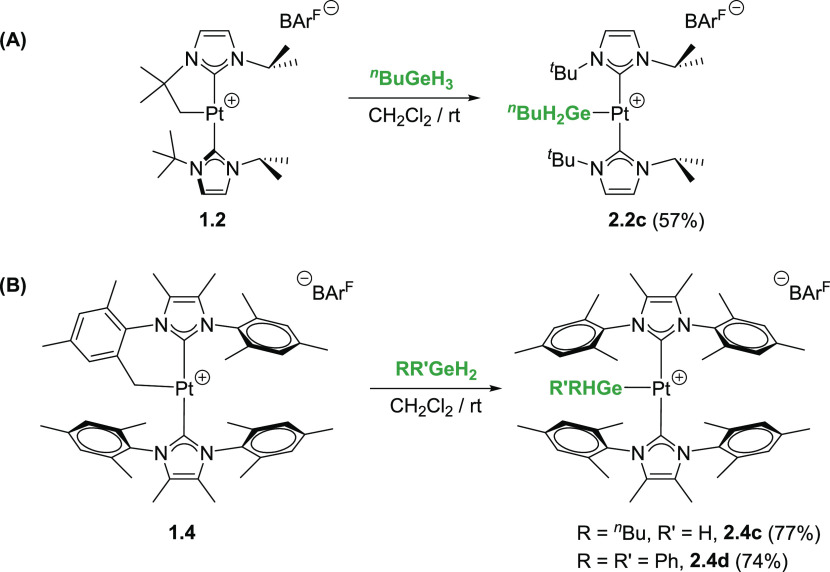
Reactions of Complexes **1.2** (A) and **1.4** (B) with Primary and Secondary Hydrogermanes

Following our previous procedure for the reaction of complexes [Pt(NHC′)(NHC)][BAr^F^] (**1.1**–**1.3**) with hydrosilanes,^4b,4c^ we have investigated their reactivity toward tertiary, secondary, and primary hydrogermanes. Initially, we focused our attention on tertiary hydrogermanes, since the parent hydrosilanes led to the formation of the corresponding platinum-silyl derivatives [Pt(SiR_3_)(NHC)_2_][BAr^F^].^4c^ In the same way, the reaction of complexes [Pt(NHC′)(NHC)][BAr^F^] with HGeEt_3_ or HGePh_3_ resulted in the generation of the germyl complexes [Pt(GeR_3_)(NHC)_2_][BAr^F^] through the formal C–H and Pt–Ge bond-forming processes (Scheme 2). The reaction proceeds smoothly at room temperature in dichloromethane, although the reaction times vary depending on the hydrogermane used and the *N*-heterocyclic carbene ligands. Thus, very fast reactions are observed with HGePh_3_ (typically less than 10 min), whereas longer times are required for completion in the case of HGeEt_3_ (ca. 18–24 h). On the other hand, no reaction takes place with tertiary hydrogermanes and the IMes derivative [Pt(IMes*′)(IMes*)][BAr^F^], **1.4** (see Scheme 3 above). The ^1^H and ^13^C{^1^H} NMR of the products revealed a highly symmetrical environment, in agreement with the presence of two chemically equivalent NHCs. It is worth mentioning that the CH_2_ carbon atoms of the Et_3_Ge- fragment exhibit coupling to ^195^Pt nucleus in the ^13^C{^1^H} NMR (^2^*J*_Pt,C_ = 118.7 (**2.1a**), 118.5 (**2.2a**) and 155.0 (**2.3a**) Hz), providing evidence for the direct bonding of the platinum and germyl moieties. Definite proof of the structure of these compounds came from X-ray diffraction studies of complexes **2.1a**, **2.1b**, **2.2b**, and **2.3b** (Figure 1). Similar to the silyl derivatives, these compounds show a T-shaped structure in which the two NHCs are mutually *trans* and the germyl fragment occupies the third coordination site in *trans* to the formal vacant site. Agostic interactions,^6^ if present, should be weak, with the closest carbon to platinum atoms at 3.502(2) (**2.1a**), 3.086(3) (**2.1b**), 3.193(4) (**2.2b**), and 3.265(4) (**2.3b**) Å. The platinum–germanium bond distances (2.3938(6), **2.1a**; 2.3953(5), **2.1b**; 2.3881(5), **2.2b**; and 2.4075(6) Å, **2.3b**) fall between those reported for platinum(II) germyl complexes.^3g,7^

**Scheme 4:**
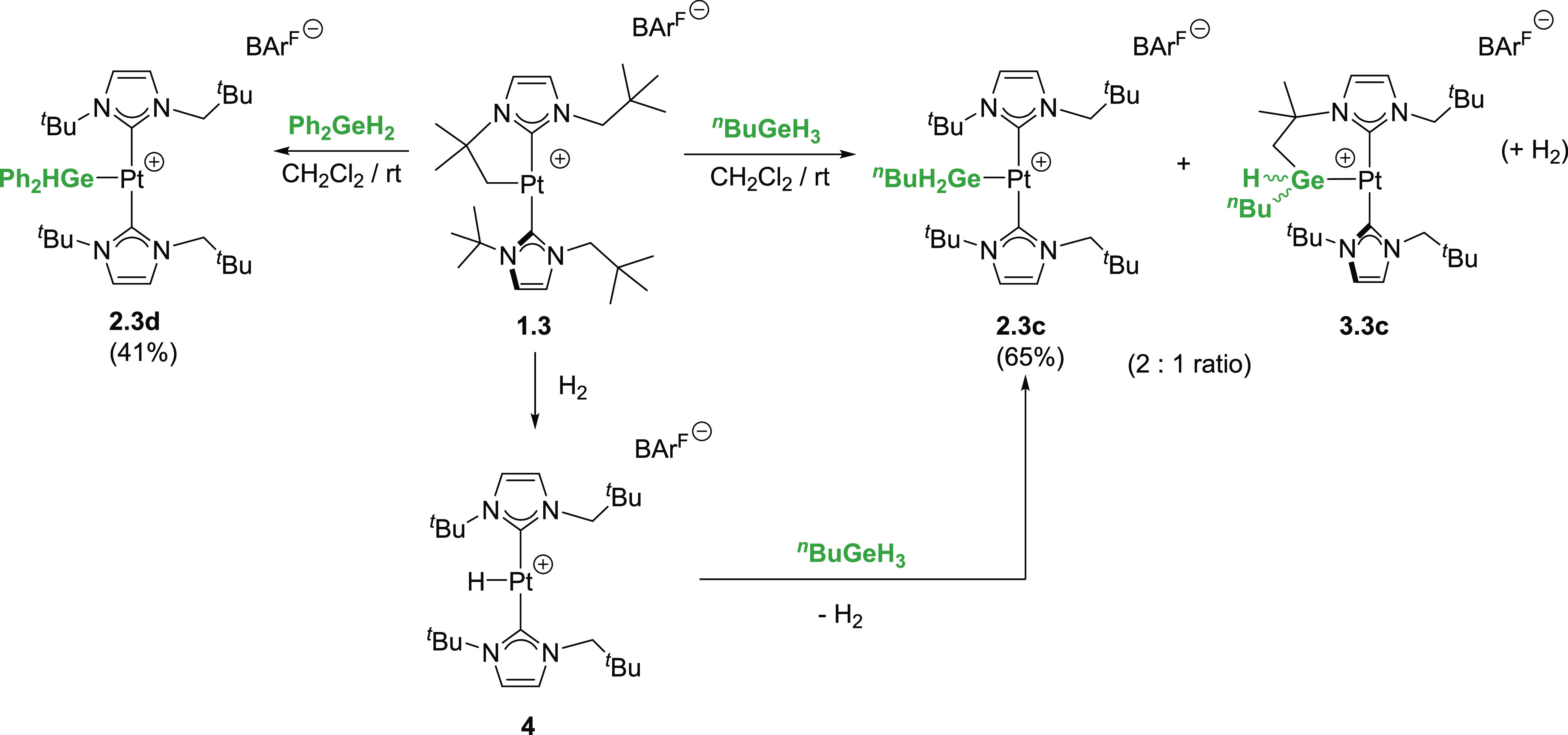
Reaction of Cyclometalated Complex **1.3** and Hydride **4** with Primary and Secondary Hydrogermanes

The reaction of complexes **1.1**–**1.4** with primary and secondary hydrogermanes has also been explored. It is worth recalling at this point that the reaction of complexes **1.2** and **1.4** with primary silanes RSiH_3_ follows a different outcome than that involving tertiary silanes. Previously, we have reported that a double Si–H bond activation takes place involving the initial formation of C–Si and Pt–H bonds (first Si–H bond activation), followed by a cyclometalation event that forges a new Pt–Si bond with extrusion of H_2_ (second Si–H activation).^4b^ However, the reaction of primary germane ^*n*^BuGeH_3_ with the cyclometalated complexes **1.2** and **1.4** yielded the platinum-germyl complexes [Pt(GeH_2_^*n*^Bu)(NHC)_2_][BAr^F^] (**2.2c** and **2.4c**), with no signs of formation of the products arising from C–Ge bond forming (Scheme 3).^8^ At variance with the silyl derivatives, for which carbon–silicon products are observed, leading to two distinct NHCs (Scheme 1A), the germyl derivatives **2.2c** and **2.4c** exhibit a highly symmetrical environment in their ^1^H and ^13^C{^1^H} NMR spectra, in agreement with the presence of two equivalent NHC ligands. In addition, a signal at 2.59 (**2.2c**) ppm with a coupling constant to ^195^Pt of ca. 190 Hz integrating for two protons has been assigned to the GeH_2_^*n*^Bu moiety.^9^ Nevertheless, the reaction of complex **1.3** with ^*n*^BuGeH_3_ follows a slightly different outcome. The ^1^H NMR of the crude reaction mixture shows the formation of two main products in ca. 2:1 ratio (Scheme 4 and Figure S42). The major compound is symmetrical, and, according to an alternative synthetic route (see below), the signals correspond to the platinum-germyl complex **2.3c**. The other species is unsymmetrical, based on the resonances for two different NHC ligands. In addition, a signal at 2.95 ppm with a *J*_Pt,H_ of 200 Hz (similar to that of complex **2.2c**, 188 Hz), with an integral value for one proton, suggests the presence of a Pt–Ge–H fragment. These data, together with the detection of H_2_ evolution, point to the possible formation of the carbon–germanium coupled product **3.3c**. Unfortunately, we have not been able to isolate this complex. Nevertheless, as mentioned above, complex **2.3c** can be obtained in pure form by a sequential process that involves hydrogenation of starting material **1.3**, leading to hydride **4**, followed by addition of ^*n*^BuGeH_3_ (with concomitant release of H_2_, as shown in Scheme 4).

**Figure 2:**
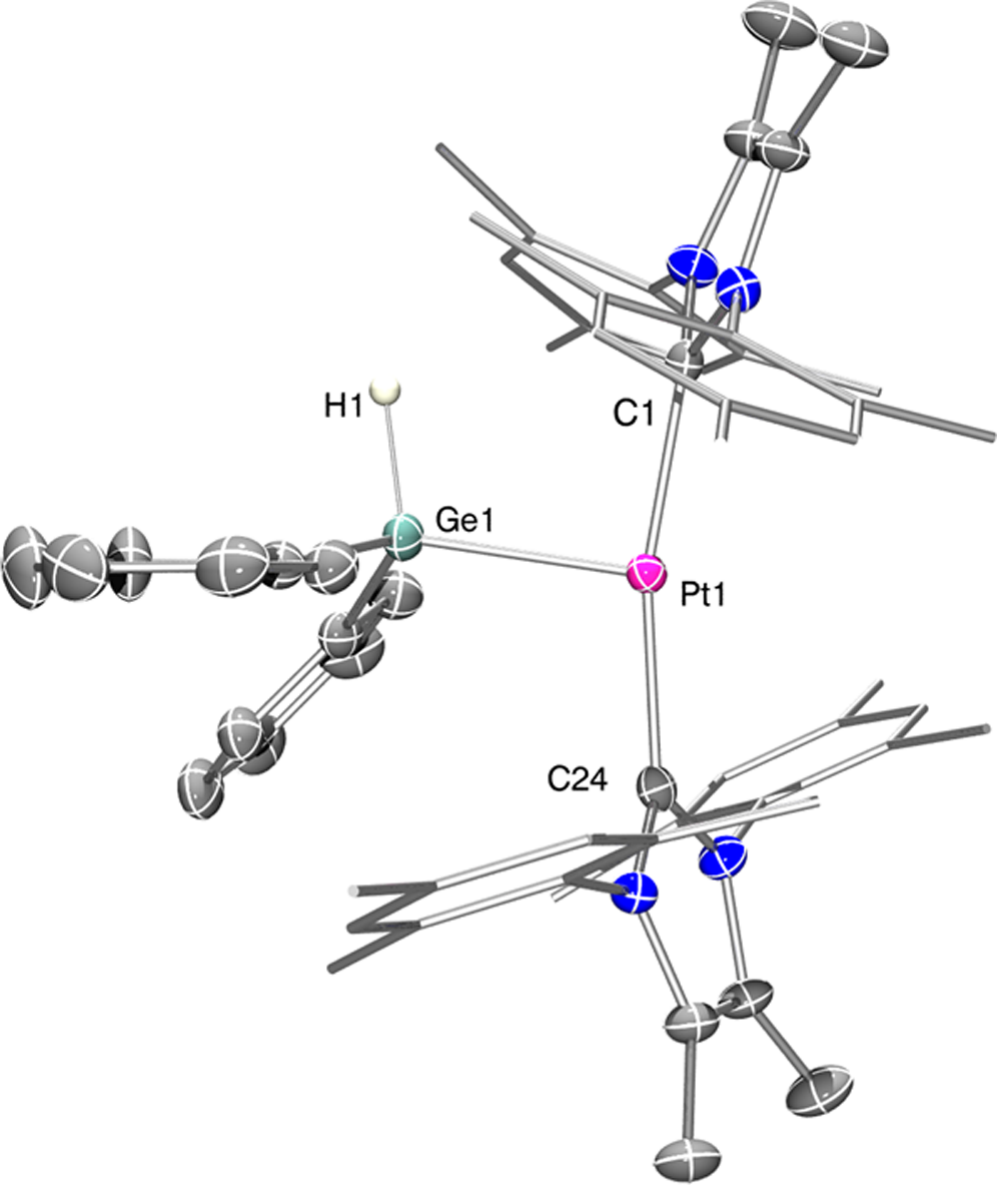
Thermal ellipsoid depiction of the cationic fragment of complex **2.4d** (hydrogen, except H1, and some carbon atoms omitted for clarity). Thermal ellipsoids at 30% probability. Selected bond distances (Å) and angles (°): Pt1-Ge1, 2.36248(6); Ge1-H1, 1.45(3); Pt1-C1, 2.025(4); Pt1-C24, 2.038(4); and C1-Pt1-C24, 167.1(2).

Interestingly, this lack of selectivity is not observed in the reaction of **1.3** and the secondary hydrogermane Ph_2_GeH_2_ (Scheme 4). In this case, the only product observed is the platinum-germyl complex [Pt(GeHPh_2_)(I^*t*^Bu^*n*^Pe)_2_][BAr^F^], **2.3d** (see the Supporting Information). This result hints at small differences in the energetic barriers, leading to either C–H/Pt–Ge or C–Ge/Pt–H bond-forming processes (see below) and suggests that sterics may be relevant in directing the reaction in one direction or another. Similarly, the reaction between complex **1.4** and diphenylgermane Ph_2_GeH_2_ leads exclusively to the platinum-germyl complex **2.4d** (Scheme 3B). As in the previous reactions, the ^1^H NMR of **2.4d** is very simple as a consequence of the high symmetry of the molecule. The most characteristic resonance in the ^1^H NMR is that of the Ge*H* proton that appears at 3.45 ppm with a large ^2^*J*_Pt,H_ of 207 Hz, which is considerably larger than that found in related hydrogermyl-platinum complexes (ca. 50–65 Hz).^3f,3g,7j^ The X-ray structure of this compound is depicted in Figure 2. Bond distances and angles are in the range of those described before, and **2.4d** can be described as a low electron count Pt(II) species, since the closest carbon–platinum distance of 3.841(7) Å (corresponding Pt–H = 3.05 Å) is too long for an agostic interaction to be present.^6^

**Scheme 5:**
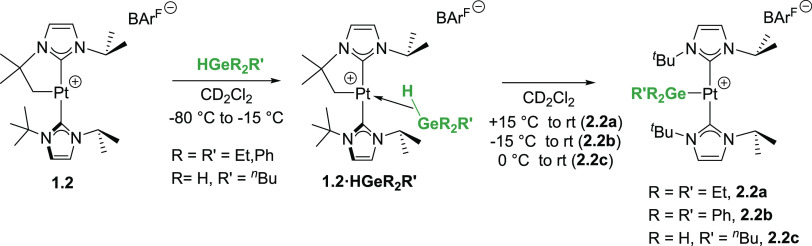
Low-Temperature NMR Experiments between Complex **1.2** and Hydrogermanes

To look for possible intermediates in the reaction of complex **1.2** with hydrogermanes R_3_GeH (R = Et, Ph) and ^*n*^BuGeH_3_ (particularly the formation of σ-GeH complexes), low-temperature NMR studies were carried out. First, we analyzed the reaction of **1.2** and Et_3_GeH in CD_2_Cl_2_ from −80 °C to room temperature. Similar to the reactions with Et_3_SiH, a clean reaction takes place, leading to the nonclassical σ-GeH complex **1.2·HGeEt**_**3**_ (Scheme 5). The most significant NMR resonance in the ^1^H NMR spectrum is that of the bridging Pt–H–Ge proton, which appears at −5.36 ppm (−60 °C) with a ^1^*J*_Pt,H_ of 400 Hz. Both the chemical shift and coupling constant are similar to those observed in the related σ-SiH and σ-BH complexes **1.2·HSiEt**_**3**_ (δ −4.90; ^1^*J*_Pt,H_ = 396 Hz) and **1.2·HBpin** (δ −3.95; ^1^*J*_Pt,H_ = 357 Hz) but significantly different to those observed in agostic Pt···H···Ge interactions (δ 0.43, ^1^*J*_Pt,H_ = 785 Hz).^3g^ Like **1.2·HSiEt**_**3**_ and **1.2·HBpin**, the diastereotopic protons of the Pt–CH_2_ fragment resonate at 2.23 and 2.06 ppm with reduced coupling constants to ^195^Pt (66 and 94 Hz at −10 °C) with respect to the cyclometalated starting material **1.2** (^2^*J*_Pt,H_ = 109 Hz). This smaller value is consistent with the presence of a weak ligand *trans* to the CH_2_–Pt fragment.^10^ The integrity of complex **1.2·HGeEt**_**3**_ is maintained at temperatures below 15 °C, but it slowly evolves over a period of 24 h at room temperature to the germyl complex **2.2a**. Analogous results were obtained in the reaction of **1.2** and Ph_3_GeH. At low temperatures, formation of **1.2·HGePh**_**3**_ is characterized by a ^1^H NMR spectrum, with a signal in the hydride region at −4.68 ppm (^1^*J*_Pt,H_ = 396 Hz, at −80 °C, cf. **1.2**·**HSiPh**_**3**_ analogue resonates at −4.00 ppm with ^1^*J*_Pt,H_ = 410 Hz) (see the Supporting Information). Complex **1.2·HGePh**_**3**_ is considerably less stable, and it starts rearranging into **2.2b** at −15 °C, being fully transformed within a few minutes at 15 °C. This different behavior with respect to Et_3_GeH is very likely a consequence of the increased electronegativity of the Ph groups compared to Et fragments, which lowers the energy of the σ*(Ge–H) molecular orbital, allowing a more efficient backdonation from the platinum atom.^1a,2d,3b,11^ These results are in good agreement with those observed for Ph_3_SiH and Et_3_SiH.^4c^

Finally, the case of ^*n*^BuGeH_3_ was examined. The different behavior of this hydrogermane in comparison to the parent hydrosilane ^*n*^BuSiH_3_ is somewhat puzzling. One possibility is that a reversible C–Ge/Pt–H coupling reaction is taking place similar to that observed in the reaction of tricoordinated boranes HBR_2_ with complex **1.2** (Scheme 1B):^4a^ a C–B bond coupling process occurs in a first step, but this reaction is reversible, leading eventually to the thermodynamically favored platinum-boryl complex Pt–BR_2_. Therefore, to look for similar potential intermediates, low-temperature NMR studies were undertaken. Upon mixing **1.2** with ^*n*^BuGeH_3_ at −40 °C, the exclusive formation of the σ-GeH complex was detected. As in the previous systems, the bridging Pt–H–Ge proton resonates at −5.59 ppm (^1^*J*_Pt,H_ = 416 Hz), whereas the terminal GeH protons appear as broad signals at 4.81 and 4.52 ppm, that is, downfield shifted with respect to free ^*n*^BuGeH_3_ (ca. 3.5 ppm). Then, upon increasing the temperature to 0 °C, the final product **2.2c** begins to form without detecting any other intermediates during the course of the transformation. Thus, the platinum-germyl complex is, very likely, both kinetically and thermodynamically favored over the products deriving from C–Ge bond coupling. As detailed below, density functional theory (DFT) calculations support this hypothesis.

**Figure 3:**
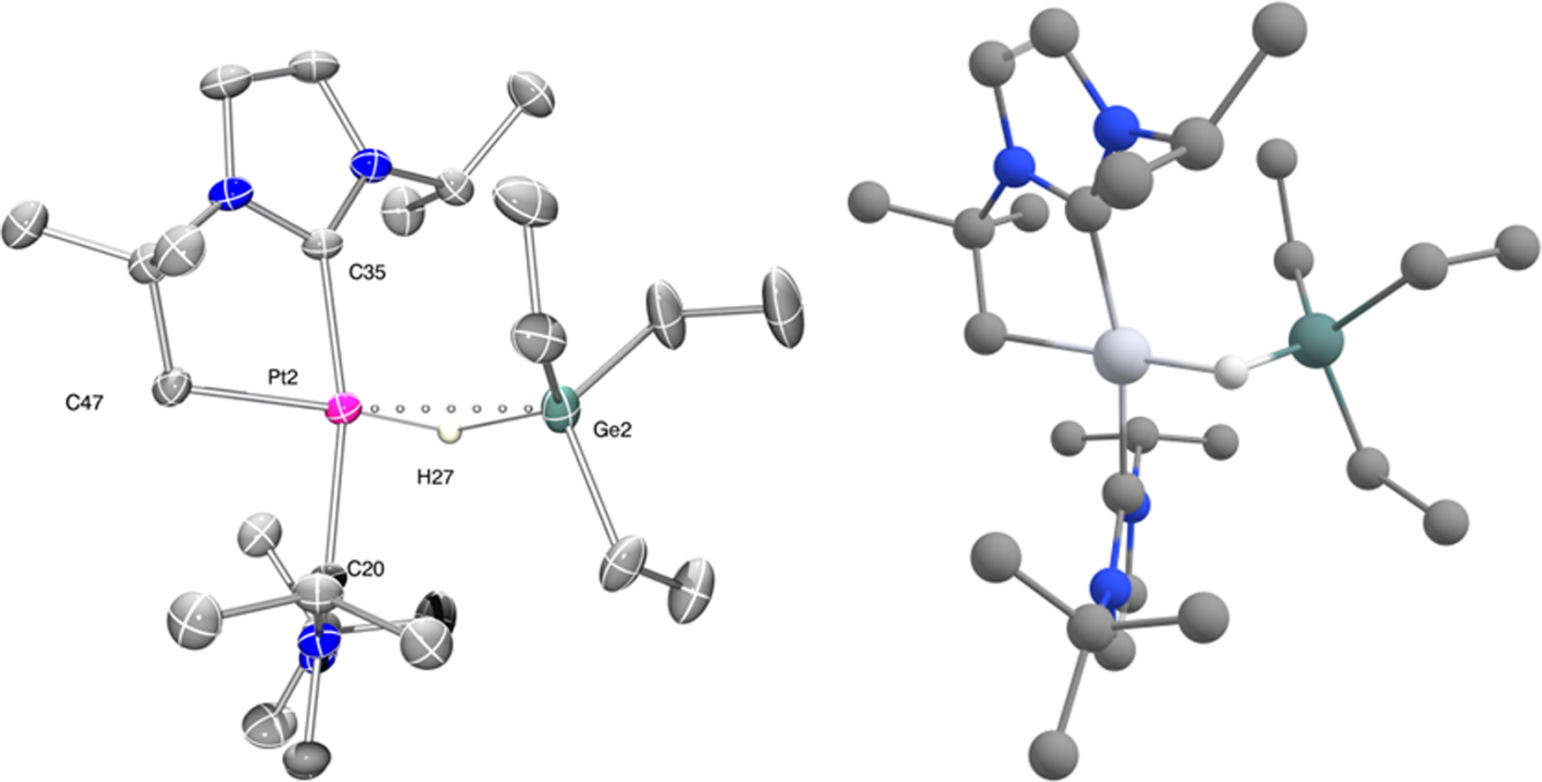
Thermal ellipsoid (left) and DFT-calculated (right) representations of **1.2·HGeEt**_**3**_. Thermal ellipsoids are set to 30% probability. BAr^F^ anion and hydrogen atoms (except H27) are omitted for clarity. Selected bond distances [Å] and angles [°]: Experimental [Theoretical]: Pt2-C20, 2.041(3) [2.062]; Pt2-C35, 2.012(2) [2.024]; Pt2-C47, 2.089(3) [2.102]; Pt2-H27, 1.41(4) [1.695]; Ge2-H27, 1.78(4) [1.786]; Pt2···Ge2, 2.6468(8) [2.659]; Pt2-H27-Ge2, 111(2) [99.6]; and C47-Pt2-H27, 172.1 [168.4], C20-Pt2-C35, 167.8 (1) [167.7].

With all of the information extracted from the NMR studies, it becomes clear that complex **1.2·HGeEt**_**3**_ appears to be the most stable of the three systems studied and should be the most accessible to be crystallized for X-ray diffraction studies. Indeed, we obtained crystals suitable for X-ray analysis of complex **1.2·HGeEt**_**3**_ by slow diffusion of a concentrated solution in CH_2_Cl_2_ into pentane at −20 °C. Figure 3 shows the thermal ellipsoid representation of complex **1.2·HGeEt**_**3**_. The metrical parameters are very similar to those observed for the parent **1.2·HSiEt**_**3**_. The cation contains the two NHC ligands in *trans*, one of which is cyclometalated, and the fourth coordination site is occupied by the hydrogermane. The Ge–H bond distance of 1.78(4) Å is elongated with respect to free germanes (1.53 Å)^3a,3b^ and in the range for other complexes having η^2^-GeH type interactions. The Pt–H bond length is rather short (1.41(4) Å) (1.58(3) for the related **1.2·HSiEt**_**3**_), although caution should be taken considering the drawbacks of X-ray diffraction to locate hydrogen atoms. Interestingly, the Pt···Ge bond separation is 2.6468(8) Å, longer than the sum of covalent radii of germanium and platinum (2.44 Å)^12^ and longer than that observed for the platinum-germyl complexes **2.1a**, **2.1b**, **2.2b**, and **2.3b**. Another remarkable metrical parameter is the angle defined by the Pt···H···Ge atoms of 111(2)°, which is wider than that observed for the silane derivative (103(2)°). All of the data point to a bonding scenario halfway between a η^2^- and η^1^-type. To shed light into the coordination mode of the germane in **1.2·HGeEt**_**3**_, the nature of the bonding interaction between the cationic platinum complex **1.2** and the germane HGeEt_3_ has been analyzed in detail.

**Figure 4:**
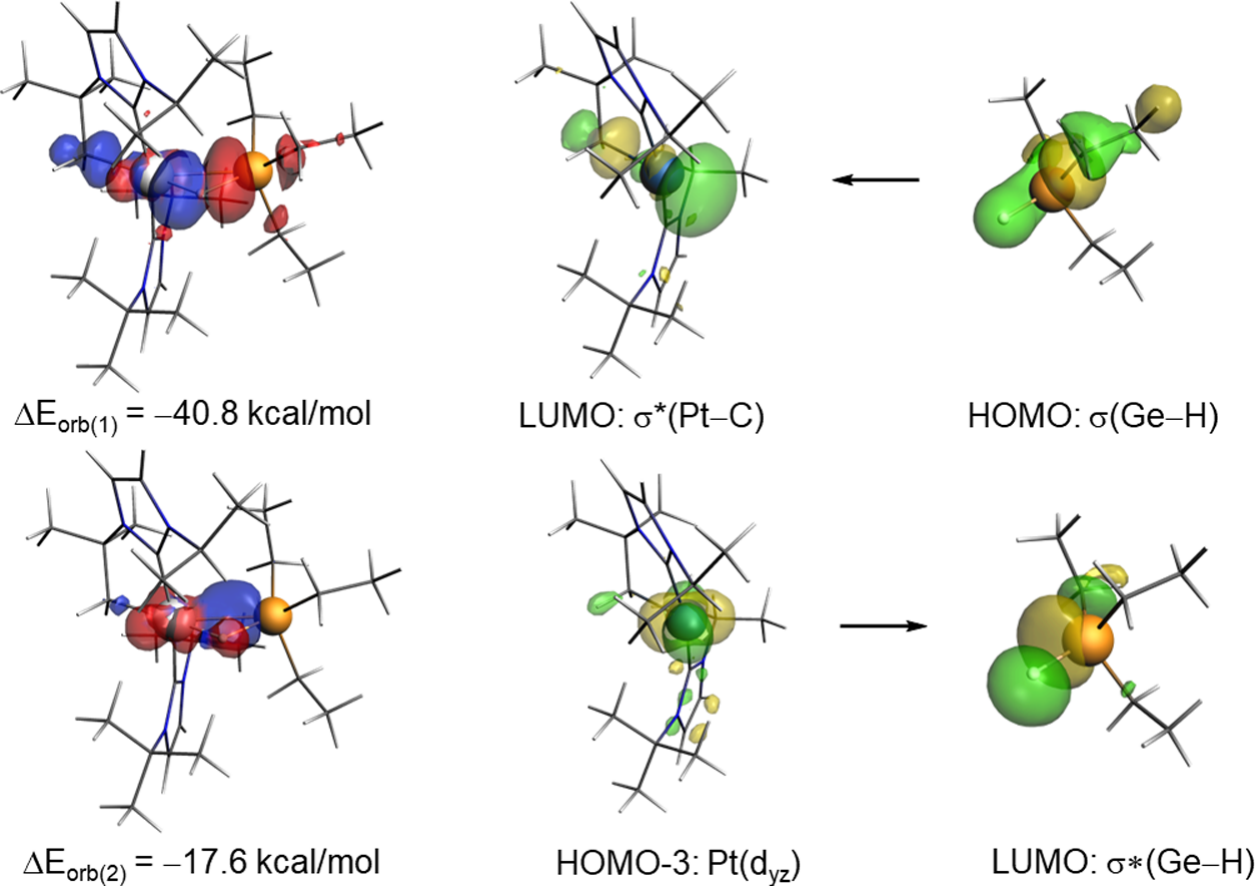
Deformation densities and associated molecular orbitals of the most important orbital interactions, Δ*E*_orb_(1) and Δ*E*_orb_(2), in complex **1.2·HGeEt**_**3**_. The color code used to represent the flow of charge is red→blue. All data were computed at the ZORA–BP86-D3/TZ2P//RI-BP86-D3/def2-TZVPP level. Results from EDA-NOCV (in kcal mol^–1^): Δ*E*_Pauli_ = 185.4; Δ*E*_elstat_ = −137.4; Δ*E*_orb_ = −78.1; Δ*E*_disp_ = −26.2; and Δ*E*_int_ = −56.3 (see the Supporting Information for a description of each term).

To this end, the energy decomposition analysis (EDA) in combination with the natural orbital for chemical valence (NOCV) method was applied at the relativistic and dispersion-corrected ZORA–BP86-D3/TZ2P//RI-BP86-D3/def2-TZVPP level. This methodology has been chosen to enable a direct comparison with the data recently reported by us for its silicon counterpart σ-SiH complex **1.2·HSiEt**_**3**_.^13^ Once again, it is confirmed that the main contribution to the total interaction (Δ*E*_int_) between the [Pt]^+^ and HGeEt_3_ fragments in complex **1.2·HGeEt**_**3**_ comes from the electrostatic attractions (Δ*E*_elstat_), which represent ca. 57% of the total attractive interactions, and are almost twice as strong as the orbital interactions (Δ*E*_orb_), contributing ca. 32% to the total bonding. The partitioning of the Δ*E*_orb_ into pairwise orbital contributions by means of the NOCV method indicates that two main interactions dominate the total orbital interactions, namely, the donation from the σ(Ge–H) molecular orbital to a vacant σ*(Pt–C) (denoted Δ*E*_orb_(1), Figure 4) and the backdonation from a doubly occupied d atomic orbital of the transition metal to the vacant σ*(Ge–H) molecular orbital (denoted Δ*E*_orb_(2)). Similar to **1.2·HSiEt**_**3**_, the σ(Ge–H) σ*(Pt–C) interaction is significantly stronger (Δ*E*_orb_(1) = −40.8 kcal mol^–1^) than the d(Pt) → σ*(Ge–H) backdonation (Δ*E*_orb_(2) = −17.6 kcal mol^–1^, Figure 4). These values are rather similar to those reported for the analogous platinum σ-SiH complex (Δ*E*_orb_(1) = −41.8 kcal mol^–1^ and Δ*E*_orb_(2) = −20.7),^13^ thus indicating that the donor and acceptor abilities of the σ(Ge–H) bond strongly resembles those of σ(Si–H) bonds. Despite that, it becomes evident that the backdonation in this germane complex is substantial, which suggests a coordination mode intermediate between the extreme situations represented by η^1^ (i.e., no backdonation) and η^2^ bonding modes.

The reaction between **1.2** and ^*n*^BuGeH_3_ was chosen as a representative process to be studied computationally^14^ to explore the mechanism by which these germyl complexes are formed. Following our previous studies on σ-SiH and σ-BH complexes, different pathways can be envisaged, starting from σ-GeH complex **1.2·HGeH**_**2**_^***n***^**Bu**. On the one hand, hydride transfer from the germane to the CH_2_ moiety would forge C–H and Pt–Ge bonds, giving rise to the aforementioned germyl species (C–H bond formation pathway). On the other hand, this hydride could instead form a Pt–H bond with concomitant formation of a C–Ge linkage (C–Ge bond formation pathway), which might account for the observation of complexes like **3.3c** (Scheme 4). Additionally, the relatively small volume of the NHC fragments allows *cis*(4c,^15^) or *trans* geometries of these ligands around the metal center. Thus, DFT calculations were carried out for each mechanistic pathway (i.e., C–H or C–Ge coupling) considering both isomers.

**Figure 5:**
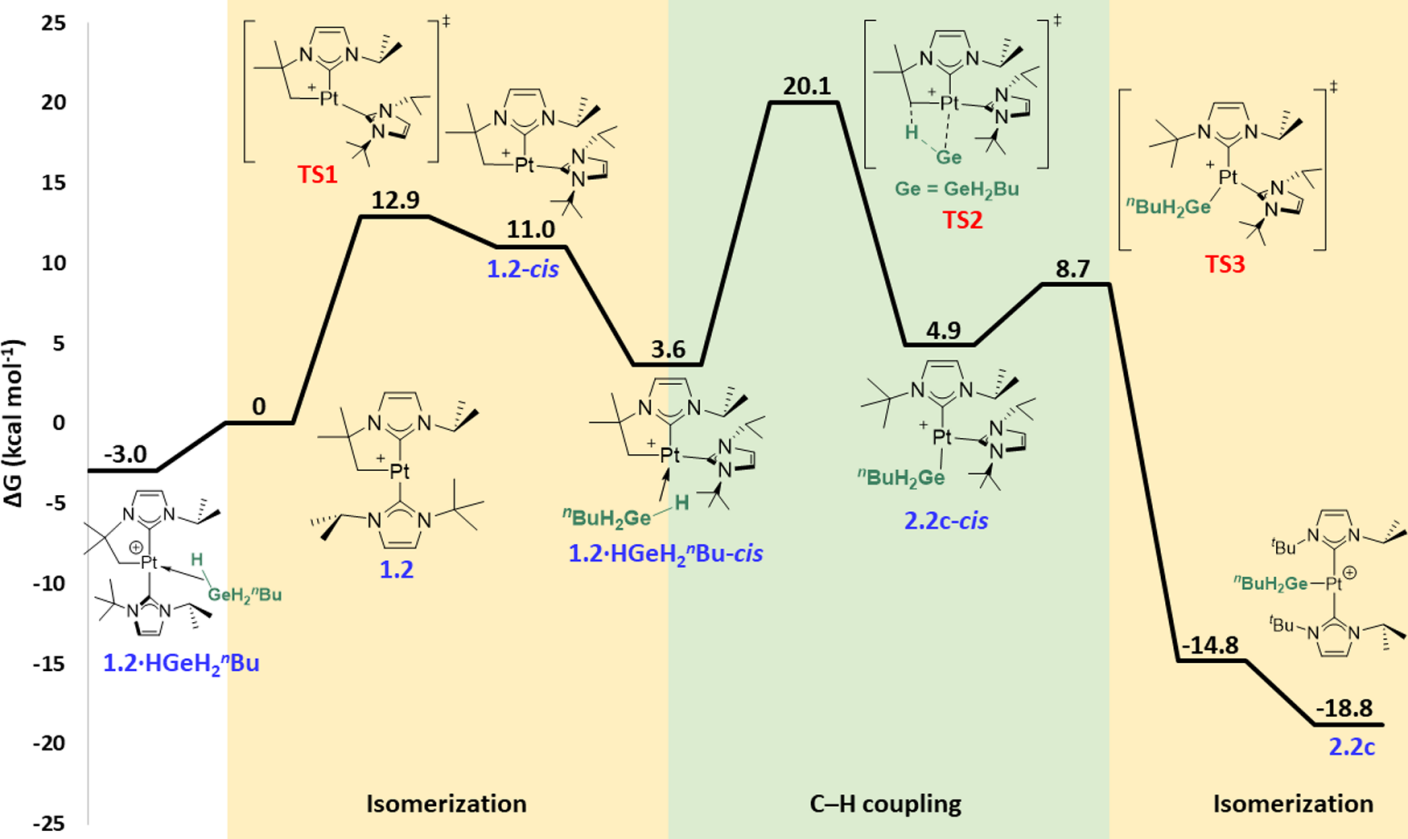
Computed Gibbs energy profile in dichloromethane for the C–H coupling pathway (NHCs in *cis*) upon reaction between **1.2** and ^*n*^BuGeH_3_. Gibbs energies computed at 298 K are given in kcal mol^–1^. The Gibbs energy of **1.2** + ^*n*^BuGeH_3_ has been taken as zero-energy.

First, the C–H bond pathway was calculated based on the experimental selective formation of the platinum-germyl complexes and the lack of observed intermediates during low-temperature NMR experiments. Whereas the process involving the NHC ligands in a *trans* geometry requires overcoming a kinetic barrier of 27.7 kcal mol^–1^ for the oxidative addition of the germane (Figure S48), analysis of the analogous reaction involving the NHC ligands in a *cis* arrangement gave lower energy barriers (Figure 5). As expected, germane coordination to give **1.2·HGeH**_**2**_^***n***^**Bu** is thermodynamically favored, since the σ-GeH complex is 3.0 kcal mol^–1^ more stable than **1.2** (energy reference). This species exhibits Pt–H and Pt···Ge distances of 1.78 and 2.77 Å, respectively, and a Pt–H–Ge angle of 107.8°, in line with the metrics mentioned above for similar σ-GeH species. *Trans*-*cis* isomerization from **1.2** through **TS1** (12.9 kcal mol^–1^) yields cyclometalated Pt(II) complex **1.2**-***cis*** (11.0 kcal mol^–1^).^4a,16^ In this isomerization process, the C_NHC_–Pt–C_NHC_ contracts from 176.4 to 112.5°. From this point, ^*n*^BuGeH_3_ can bind the metal center to form the σ complex **1.2·HGeH**_**2**_^***n***^**Bu-*****cis***, 3.6 kcal mol^–1^ above the origin. Contrary to that observed for σ-BH complexes,^4a^ the *cis* σ-GeH species did not clearly exhibit an orientation of the germane molecule that would place the Ge or H atoms close to the CH_2_ moiety. Formation of the C–H/Pt–Ge bonds takes place through **TS2** (23.1 kcal mol^–1^ barrier), which involves H transfer (Pt–H = 1.60 Å, Pt···Ge = 2.55 Å, Ge···H = 2.08 Å, Pt–H–Ge = 86.6°) to the methylene unit and formation of germyl species **2.2c-*****cis*** (4.9 kcal mol^–1^ above the energy reference). This transition state gives the *cis* isomer of the observed product in a concerted fashion through a σ-CAM (σ-complex assisted metathesis) mechanism,^17^ similar to that observed for σ-BH complexes.^4a,4f^ Thus, the orientation of the NHC ligands in the complex has a direct impact on the nature and energy barriers of the resulting mechanism for the formation of germyl species (i.e., oxidative addition for a *trans* arrangement vs σ-CAM for a *cis* geometry). From **2.2c-*****cis***, *cis*-*trans* isomerization occurs via **TS3** (8.7 kcal mol^–1^), leading to germyl complex **2.2c** (−14.8 kcal mol^–1^). However, this complex possesses both NHC ligands almost coplanar (dihedral angle = 15.0°), and a different isomer with a wider dihedral angle (42.8°) between both carbene ligands was found to be more stable (−18.8 kcal mol^–1^).

**Figure 6:**
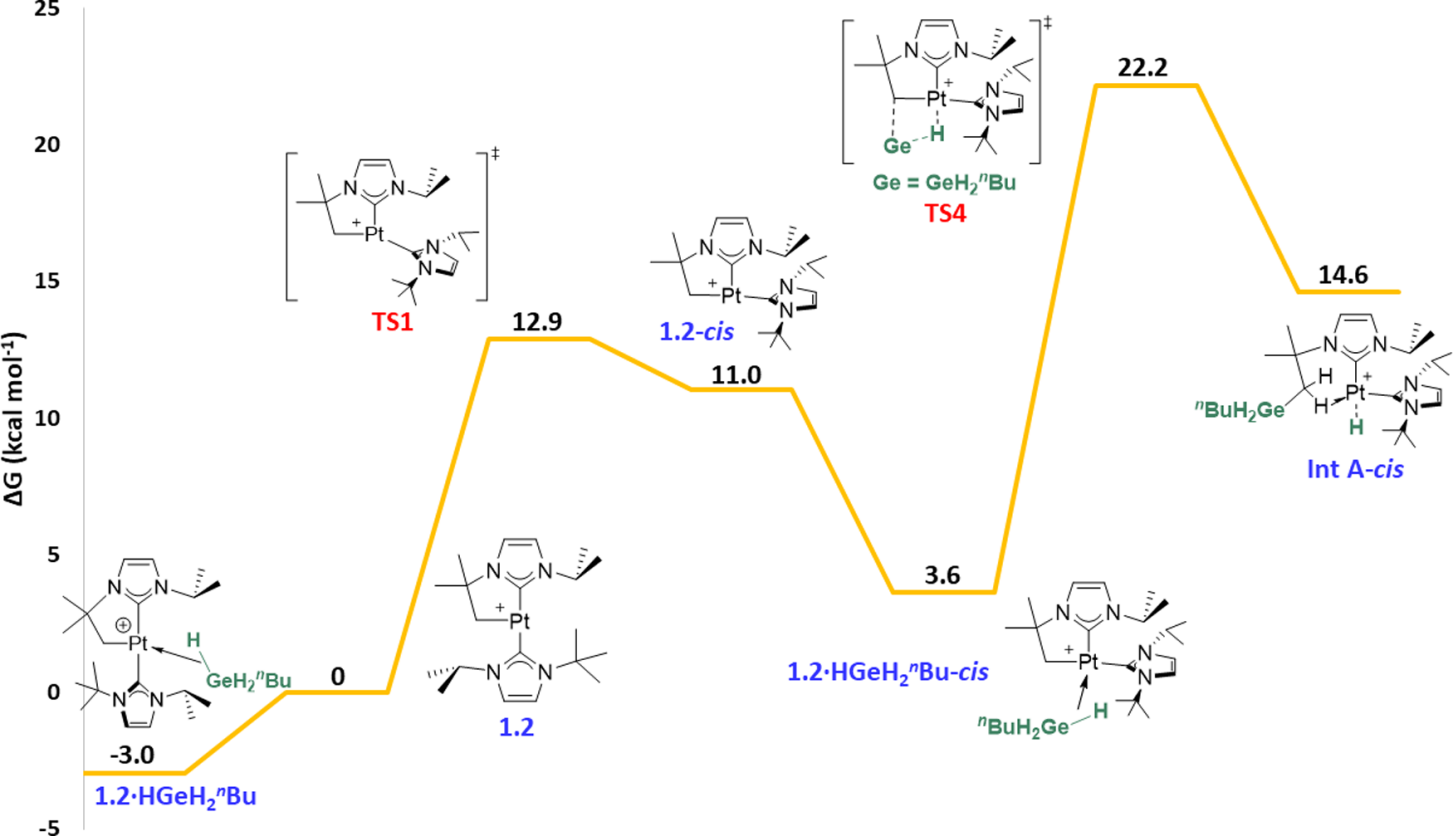
Computed Gibbs energy profile in dichloromethane for the C–Ge coupling pathway (NHCs in *cis*) upon reaction between **1.2** and ^*n*^BuGeH_3_. Gibbs energies computed at 298 K are given in kcal mol^–1^. The Gibbs energy of **1.2** + ^*n*^BuGeH_3_ has been taken as zero-energy.

**Figure 7:**
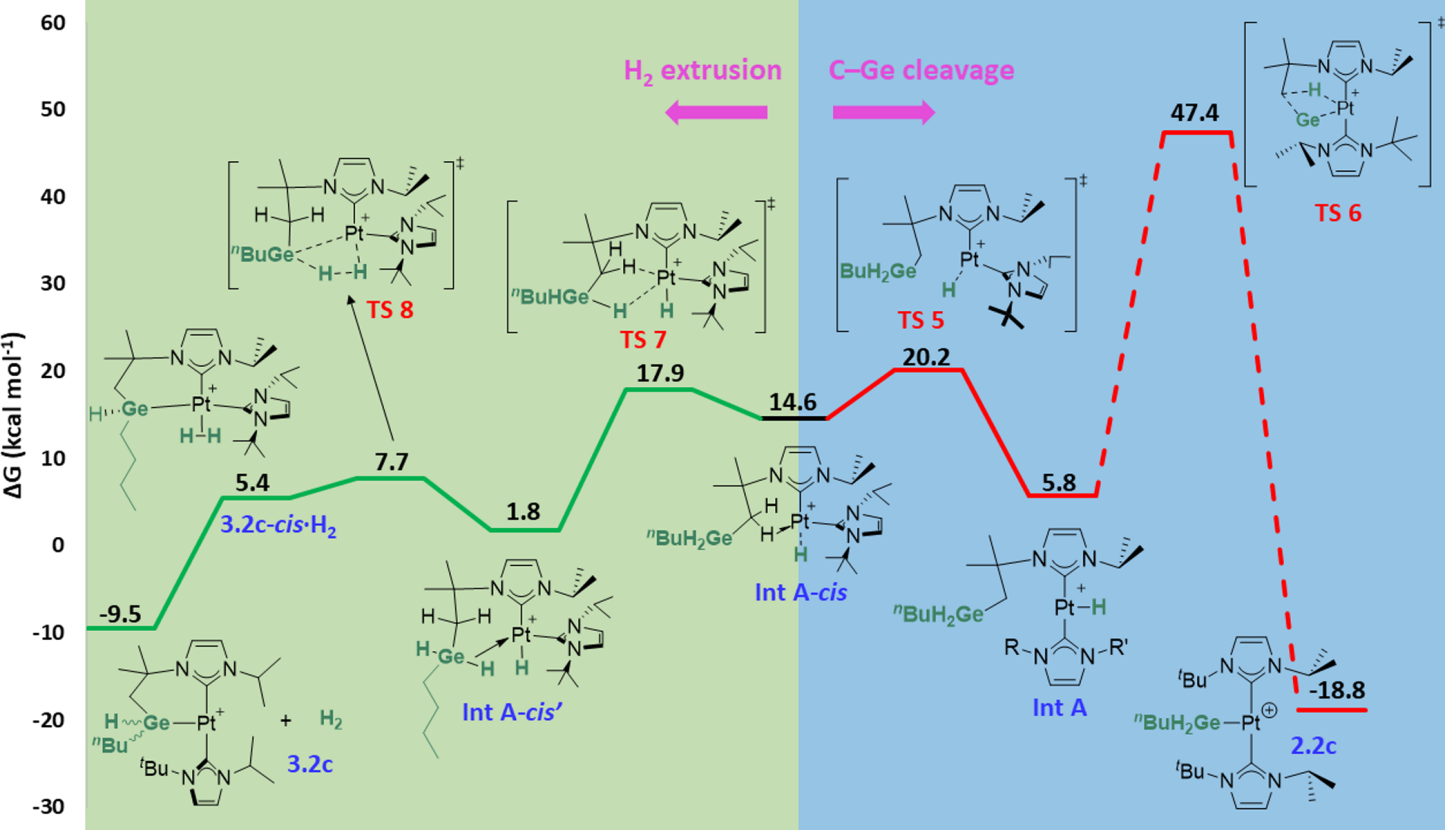
Computed Gibbs energy profile in dichloromethane for the processes that might take place after C–Ge bond formation (NHCs in *cis*), namely, H_2_ extrusion (left) or C–Ge bond cleavage (right). Gibbs energies computed at 298 K are given in kcal mol^–1^. The Gibbs energy of **1.2** + ^*n*^BuGeH_3_ has been taken as zero-energy.

Next, the C–Ge bond formation pathways were calculated. The mechanism for the system possessing both NHC ligands in a *cis* arrangement is displayed in Figure 6 (the analogous C–Ge coupling pathway involving both NHC ligands in *trans* requires a barrier of 27.4 kcal mol^–1^, see Figure S46). While the first isomerization part is identical to that shown in Figure 5, **TS4** (22.2 kcal mol^–1^ energy barrier, 25.2 kcal mol^–1^ overall) describes the C–Ge and Pt–H coupling in a concerted step (σ-CAM process). Whereas the Pt–H (1.62 Å) and Pt···Ge (2.69 Å) distances are similar to those observed in **TS2**, the Ge···H distance is much longer (2.55 Å). Indeed, the geometry of **TS4** and the energy of **Int A-*****cis*** (14.6 kcal mol^–1^) indicate that **TS4** is a late transition state. Species **Int A-*****cis*** exhibits an agostic interaction^6a^ (Pt–H = 1.89 Å, Pt–H–C = 105.7°), stabilizing the vacant position on platinum through one of the C–H bonds of the methylene group. From this geometry, all attempts to cleave the C–Ge bond leading to a *cis* germyl species **2.2c-*****cis*** (Figure 5) have proven futile, given that the σ-GeH complex was observed instead, in line with our observations of σ-BH complexes.^4a^ Therefore, cleaving the C–Ge bond from a structure with both NHC ligands in a *trans* geometry was calculated instead (Figure 7, C–Ge cleavage pathway). Thus, isomerization through **TS5** (20.2 kcal mol^–1^) is necessary, giving complex **Int A**, 5.8 kcal mol^–1^ above the energy reference. Unlike **Int A-*****cis***, this species does not possess an agostic interaction. From here, a concerted process can take place in which the hydride ligand bound to platinum is transferred to the CH_2_ moiety at the same time the germyl fragment forms a bond with the metal center. Nevertheless, this step is too energy-demanding (**TS6**, 47.4 kcal mol^–1^) and not feasible under the reaction conditions experimentally employed, which might explain why these C–Ge coupling species are observed in some cases like **3.3c** (Scheme 4). Indeed, this alternative pathway was also explored by DFT methods (Figure 7, H_2_ extrusion pathway), which would first proceed via **TS7** (17.9 kcal mol^–1^), where the C–H bond of the agostic interaction in **Int A-*****cis*** is replaced by one of the Ge–H bonds, yielding **Int A-*****cis***′ (1.8 kcal mol^–1^). This complex exhibits shorter contacts of the germane fragment with the metal compared to that observed in **1.2·HGeH**_**2**_^***n***^**Bu**, according to key bonding metrics [Pt–H–Ge = 99.5° (vs 107.8°), Pt···Ge = 2.62 Å (vs 2.77 Å), Ge–H = 1.74 Å (vs 1.65 Å)]. This might be due to the reduced steric bulk around the germane moiety since one of the NHC ligands is now *trans* to it. From this geometry, Pt–Ge and H–H bond formation occurs through a σ-CAM process in **TS8** (7.7 kcal mol^–1^). Then, **3.2c-*****cis*****·H**_**2**_ (5.4 kcal mol^–1^) is obtained, which exhibits a 6-membered germametalacycle and a coordinated H_2_ molecule (H–H = 0.85 Å). Finally, dihydrogen dissociation directly leads to a *trans* geometry of both carbene ligands (**3.2c**, −9.5 kcal mol^–1^), analogous to that observed for C–Si coupling products.^4b^ A similar H_2_ extrusion mechanism involving both NHC ligands in a *trans* arrangement gave higher energy barriers compared to the *cis* pathway (see Figure S47).

In light of the aforementioned energy profiles, it is reasonable to propose that the formation of the cationic Pt(II) germyl complexes experimentally obtained can occur through a mechanism in which both NHC ligands are in a *cis* geometry, given the lower barriers computed for both C–H and C–Ge coupling compared to their *trans* analogues. In particular, the C–H bond formation pathway (Figure 5) involving NHC ligands in *cis* seems to be the most likely mechanism, given that it best describes the evolution of the system along the reaction coordinate, with the lowest kinetic barrier (23.1 kcal mol^–1^) of the computed profiles. Then, although C–Ge coupling seems energetically feasible as well (Figure 6), it is higher in energy (25.2 kcal mol^–1^), which might explain why no intermediates possessing C–Ge bonds are observed (unlike C–B coupling processes using the same Pt system).^4a^ Nonetheless, this energy difference is relatively small, and if some C–Ge product is formed, it can enter the H_2_ extrusion pathway (with smaller kinetic barriers, Figure 7), leading to the corresponding germametalacycle. This small energy gap might then account for the experimentally detected amounts of complex **3.3c** (where isopropyl has been replaced by neopentyl). A summary of the highest kinetic barriers for all of the computed reaction pathways can be found in Table 1.

**Table 1:** Summary of the Highest Kinetic Barriers for the Computed Reaction Mechanisms^a^

mechanism	C–H coupling (*cis*)	C–H coupling (*trans*)	C–Ge coupling (*cis*)	C–Ge coupling (*trans*)	C–Ge cleavage (*cis*)	C–Ge cleavage (*trans*)	H_2_ extrusion (*cis*)	H_2_ extrusion (*trans*)
Δ*G* (kcal mol^–1^)	23.1	27.7	25.2	27.4	not found (gives back **2.2c-*****cis***)	50.4	20.9	25.6

a NB: C–Ge and H_2_ extrusion pathways are subsequent steps following the C–Ge coupling process.

**Scheme 6:**
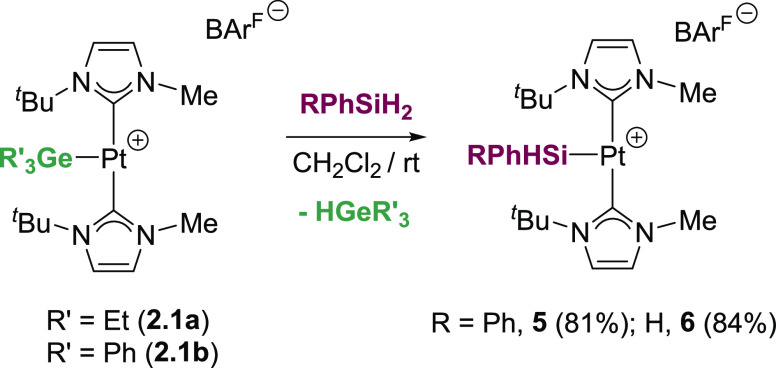
Exchange Reactions of Complexes **2.1a,b** and Hydrosilanes

Finally, to gain some information about the reactivity of some of these platinum-germyl complexes, exchange reactions with silanes have been carried out. To this aim, complexes **2.1a,b** were treated with a slight excess of Ph_2_SiH_2_ or PhSiH_3_ (Scheme 6). The reaction proceeds smoothly at room temperature, yielding the platinum-silyl complexes [Pt(SiHPhR)(I^*t*^BuMe)_2_][BAr^F^] (R = Ph, **5**; R = H, **6**). The process is rather fast for triphenylgermyl derivative **2.1b** (full conversion in less than 1 h), whereas it is slow for triethylgermyl-complex **2.1a** (full conversion after 24 h). Both reactions are very clean and serve as an alternative way to obtain this type of product without producing C–Si coupling products (as in Scheme 1A). No exchange was observed when Ph_3_SiH was used, suggesting (at least in part) that sterics play an important role in the process. Another peculiarity of the reaction is that this behavior appears to be opposite to previous exchange processes in which the metal-germyl compounds are more stable than the corresponding silyl derivatives, and thus they can be synthesized by exchange reactions from the silyl derivatives. This effect has been attributed to the higher ease of cleavage of Ge–H bonds in comparison to Si–H bonds.^3b,18^ However, other factors such as steric constraints and the strength of the Pt–E (E = Si, Ge) bond generated should be taken into consideration to explain our results.

## Conclusions

The platinum-cyclometalated complexes [Pt(NHC′)(NHC)][BAr^F^] react with primary, secondary, and tertiary germanes to generate the platinum-germyl derivatives [Pt(GeR_3_)(NHC)_2_][BAr^F^], arising from C–H/Pt–Ge reaction pathways. At variance with reactions with primary silanes, the competitive process leading to C–Ge/Pt–H bonds is not observed when primary or secondary germanes are used, with the exception of the reaction of complex **1.3** with ^*n*^BuGeH_3_, in which mixtures of compounds seem to be formed in different ratios. DFT calculations suggest that the mechanism involves a σ-CAM process through a *trans*-to-*cis* isomerization of the NHCs in which the barriers computed for the formation C–H/Pt–Ge bonds are lower (ca. 2 kcal mol^–1^) than those for C–Ge/Pt–H bonds. The relatively small energetic difference might explain why in the case reaction of complex **1.3** with ^*n*^BuGeH_3_ both types of processes are observed. Low-temperature NMR studies allowed us to detect σ-GeH complexes [Pt(η^2^-HGeR_3_)(NHC′)(NHC)][BAr^F^] as intermediates, one of which was sufficiently stable to be characterized by X-ray diffraction studies. The solid-state structure of complex [Pt(HGeEt_3_)(I^*t*^Bu^*i*^Pr′)(I^*t*^Bu^*i*^Pr)][BAr^F^] indicates that the coordination mode of the Ge–H fragment falls between η^2^ and η^1^, which is consistent with the significant backdonation from the cationic platinum fragment into the σ*(Ge–H) molecular orbital suggested by the EDA-NOCV method. The results shown in this contribution are, therefore, complementary to those previously reported for hydrosilanes (in which irreversible formation of C–Si bonds are observed with primary silanes) and tricoordinated hydroboranes (with processes involving reversible formation of C–B bonds).

## Supporting Information Available

Synthetic and experimental procedures, characterization of new compounds, X-ray crystallographic details, and computational methods (PDF)

## References

[ref1] aWhited M. T.; Taylor B. L. H. Metal/organosilicon complexes: structure, reactivity, and considerations for catalysis. Comments Inorg. Chem. 2020, 40, 217–276. 10.1080/02603594.2020.1737026.

[ref2] aHandzlik J.; Kochel A.; Szymańska-Buzar T. H–Ge bond activation by tungsten carbonyls: An experimental and theoretical study. Polyhedron 2012, 31, 622–637. 10.1016/j.poly.2011.10.040.

[ref3] aSmart K. A.; Mothes-Martin E.; Vendier L.; Perutz R. N.; Grellier M.; Sabo-Etienne S. A ruthenium dihydrogen germylene complex and the catalytic synthesis of digermoxane. Organometallics 2015, 34, 4158–4163. 10.1021/acs.organomet.5b00570.

[ref4] aRíos P.; Martín-de la Calle R.; Vidossich P.; Fernández-de-Córdova F. J.; Lledós A.; Conejero S. Reversible carbon–boron bond formation at platinum centers through σ-BH complexes. Chem. Sci. 2021, 12, 1647–1655. 10.1039/D0SC05522K. PMC8179255 34163924

[ref5] aSelmani A.; Schoetz M. D.; Queen A. E.; Schoenebeck F. Modularity in the Csp^3^ space–alkyl germanes as orthogonal molecular handles for chemoselective diversification. ACS Catal. 2022, 12, 4833–4839. 10.1021/acscatal.2c00852.

[ref6] aBrookhart M.; Green M. L. H.; Parkin G. Agostic interactions in transition metal compounds. Proc. Natl. Acad. Sci. U.S.A. 2007, 104, 6908–6914. 10.1073/pnas.0610747104. 17442749 PMC1855361

[ref7] aKarimi M.; Tabei E. S.; Fayad R.; Saber M. R.; Danilov E. O.; Jones C.; Castellano F. N.; Gabbaï F. P. Photodriven elimination of chlorine from germanium and platinum in a dinuclear Pt^II^→Ge^IV^ complex. Angew. Chem., Int. Ed. 2021, 60, 22352–22358. 10.1002/anie.202107485. 34399026

[ref8] The reaction of complex **1.1** with GeH_3_^*n*^Bu initially leads to a complex that appears to be the platinum germyl species [Pt(GeH_2_^n^Bu)(I^*t*^BuMe)_2_][BAr^F^]. However, this compound decomposed both in solution as well as during the purification process to a very complex mixture of compounds.

[ref9] The signal for the GeH_2_ protons in complex **2.4c** is masked by the resonances of the CH_3_ groups of the IMes* ligand, and therefore no coupling constant to ^195^Pt could be obtained.

[ref10] Ortuño M. A.; Conejero S.; Lledós A. True and masked three-coordinate T-shaped platinum(II) intermediates. Beilstein J. Org. Chem. 2013, 9, 1352–1382. 10.3762/bjoc.9.153. 23946831 PMC3740817

[ref11] McGrady G. S.; Sirsch P.; Chatterton N. P.; Ostermann A.; Gatti C.; Altmannshofer S.; Herz V.; Eickerling G.; Scherer W. Nature of the bonding in metal-silane σ-complexes. Inorg. Chem. 2009, 48, 1588–1598. 10.1021/ic8019777. 19146446

[ref12] Pyykkö P.; Atsumi M. Molecular single-bond covalent radii for elements 1–118. Chem. - Eur. J. 2009, 15, 186–197. 10.1002/chem.200800987. 19058281

[ref13] Ríos P.; Conejero S.; Fernández I. Bonding situation of σ-E-H complexes in transition metal and main group compounds. Chem. - Eur. J. 2022, 28, e202201920 10.1002/chem.202201920. 35900796 PMC9804526

[ref14] Calculations were carried out using the M06 functional in dichloromethane (SMD). Further details and references can be found in the Supporting Information file.

[ref15] Fortman G. C.; Scott N. M.; Linden A.; Stevens E. D.; Dorta R.; Nolan S. P. Unusual reactivities of *N*-heterocyclic carbenes upon coordination to the platinum(II)–dimethyl moiety. Chem. Commun. 2010, 46, 1050–1052. 10.1039/b920482b. 20126709

[ref16] *cis*-*trans* isomerization without the H–E substrate bound gives lower energy barriers. See reference 4a for some examples of isomerization of σ-BH complexes.

[ref17] aPerutz R. N.; Sabo-Etienne S.; Weller A. S. Metathesis by partner interchange in σ-bond ligands: expanding applications of the σ-CAM mechanism. Angew. Chem., Int. Ed. 2022, 61, e202111462 10.1002/anie.202111462. PMC9299125 34694734

[ref18] Kubas G. J. Dihydrogen complexes as prototypes for the coordination chemistry of saturated molecules. Proc. Natl. Acad. Sci. U.S.A. 2007, 104, 6901–6907. 10.1073/pnas.0609707104. 17442752 PMC1855383

