# Supplementary material for: σ-GeH and Germyl Cationic Pt(II) Complexes
Source: Inorg Chem. 2022 Nov 2;61(51):20848–59. doi: 10.1021/acs.inorgchem.2c03186 (PMC9949701; doi:10.1021/acs.inorgchem.2c03186)
Supplement: Supplementary file 1 — ic2c03186_si_001.pdf [file ic2c03186_si_001.pdf]

Supporting Information

σ-GeH and germyl cationic Pt(II) complexes

Carlos J. Laglera-Gándara,[a] Pablo Ríos, [a,] Francisco José Fernández-de-Córdova,[a] Marina Barturen, [a] Israel Fernández, [b,]* Salvador Conejero [a,]**

[a] Instituto de Investigaciones Químicas (IIQ), Departamento de Química Inorgánica, Centro de Innovación en Química Avanzada (ORFEO-CINQA), CSIC and Universidad de Sevilla, Sevilla, 41092, Spain.

[b] Departamento de Química Orgánica I y Centro de Innovación en Química Avanzada (ORFEO-CINQA), facultad de Químicas, Universidad Complutense de Madrid, 28040, Madrid, Spain.

Dr. Pablo Ríos; e-mail: pablo.rios@iiq.csic.es

Prof. Israel Fernández; e-mail: israel@quim.ucm.es

Dr. Salvador Conejero; e-mail: sconejero@iiq.csic.es

Table of Contents

1. General methods S2
2. Synthesis and characterization of 1-(*tert*-butyl)-3-(*neo*-pentyl)-1*H*-imidazolium iodide, $I^tBu^nPe·HI$ S2
3. Synthesis and characterization of *cis*-$[Pt(CH_3)_2(I^tBu^nPe)_2]$ S2
4. Synthesis and characterization of $[Pt(I^tBu^nPe')(I^tBu^nPe)][BAr^F]$, **1.3** S3
5. Synthesis and characterization of germyl complexes $[Pt(GeR_3)(I^tBuMe)_2][BAr^F]$ (R = Et (**2.1a**), Ph (**2.1b**)). S4
6. Synthesis and characterization of germyl complexes $[Pt(GeR_2R')(I^tBu^iPr)_2][BAr^F]$ (R = R' = Et (**2.2a**), Ph (**2.2b**); R = H, R' = ^{n}Bu (**2.2c**); R = Ph, R' =H (**2.2d**)). S4
7. Synthesis and characterization of germyl complexes $[Pt(GeR_2R')(I^tBu^nPe)_2][BAr^F]$ (R = R' = Et **(2.3a**), Ph (**2.3b**); R = H, R' = ^{n}Bu (**2.3c**); R = Ph, R' = H (**2.3d**)). S6
8. Synthesis and characterization of germyl complexes $[Pt(GeH_2{}^nBu)(IMes^*)_2][BAr^F]$, **2.4c**, and $[Pt(GeHPh_2)(IMes^*)_2][BAr^F]$, **2.4d**. S8
9. Low temperature studies NMR studies. Characterization of complexes **1.2·HGeEt$_3$**, **1.2·HGePh$_3$** and **1.2·HGeH$_2{}^n$Bu** S8
10. Reaction of complex $[Pt(I^tBu^nPe')(I^tBu^nPe)][BAr^F]$ with $^nBuSiH_3$ S10
11. Synthesis of hydride complex $[Pt(H)(I^tBu^nPe)_2][BAr^F]$ S10
12. Synthesis and characterization of silyl complexes $[Pt(SiHPh_2)(I^tBuMe)_2][BAr^F]$, **5**, and $[Pt(SiH_2Ph)(I^tBuMe)_2][BAr^F]$, **6**. S10
13. NMR Spectra S12
14. Computational Details S33
15. Calculated energy profiles S35
16. Cartesian coordinates of the optimized structures S38

17. Crystallographic details S82
18. References S84

1. General methods

All manipulations were carried out using standard Schlenk and glovebox techniques, under an atmosphere of argon and of high purity nitrogen, respectively. All solvents were dried and degassed prior to use. n-Pentane was distilled over sodium and stored under Na/K alloy. Dichloromethane-d_2 (CD_2Cl_2) was heated under reflux over calcium hydride, distilled under Argon and stored under 3 Å activated molecular sieves. NMR spectra were recorded on Bruker DRX-500, DRX-400 and DPX-300 spectrometers, and they were referenced to external $SiMe_4$ (δ 0 ppm) using the residual protio solvent peaks as internal standard (^{1}H NMR experiments) or the characteristic resonances of the solvent nuclei (^{13}C NMR experiments). Spectral assignments were made by routine one- and two-dimensional NMR experiments where appropriate. Elemental analysis was carried out with a LECO TruSpec CHN elementary analyser. Et_3GeH, Ph_3GeH, Ph_2GeH_2, ^{n}BuGeH_3, Ph_2SiH_2 and $PhSiH_3$ were purchased from Aldrich, Gelest, ABCR or TCI, stored under argon and used as received. *Tert*-butylimidazol,[1] [H(OEt_2)][BAr^F][2] and complexes [Pt(CH_3)$_2$(cod)],[3] [Pt(I^{t}Bu^{i}Pr')(I^{t}Bu^{i}Pr)][BAr^F][4] and [Pt(I^{t}BuMe')(I^{t}BuMe)][BAr^F][5] were obtained accordingly to published procedures.

2. Synthesis and characterization of 1-(*tert*-butyl)-3-(*neo*-pentyl)-1*H*-imidazolium iodide, I^{t}Bu^{n}Pe·HI

1 g (8.05 mmol) of 1-(*tert*-butyl)-1*H*-imidazol and 1.6 mL of 2-iodoneopentane (2.39 g, 12.08 mmol) were dissolved in a J. Young flask under argon. The mixture was heated at 125 °C for 24 h, cooled down and the resulted precipitate was washed with THF (2 x 10 mL) and filtered off leading to a white pure solid (1.90 g, 73%). **^{1}H NMR** (400 MHz, CD_2Cl_2, 298 K): δ 10.15 (s, 1H, =CH), 7.54 (s, 1H, =CH), 7.38 (s, 1H, =CH), 4.30 (s, 2H, C***H***$_2$C(CH_3)$_3$), 1.72 (s, 9H, C(CH_3)$_3$), 1.02 (s, 9H, CH_2C(C***H***$_3$)$_3$). **^{13}C{^{1}H} NMR** (100 MHz, CD_2Cl_2, 298 K): δ 136.2 (=CH), 124.0 and 119.4 (=CH), 61.0 (***C***(CH_3)$_3$), 60.7 (***C***H_2C(CH_3)$_3$), 30.3 (CH_2C(***C***H_3)$_3$), 27.1 (CH_2C(***C***H_3)$_3$). Elem. Anal. Calcd. for $C_{12}H_{23}IN_2$: C, 44.73; H, 7.19; N, 8.69. Found: C, 44.61; H, 7.50; N, 8.55.

3. Synthesis and characterization of *cis*-[Pt(CH_3)$_2$(I^{t}Bu^{n}Pe)$_2$]

1-(*tert*-butyl)-3-(*neo*-pentyl)-1*H*-imidazolium iodide, I^{t}Bu^{n}Pe·HI (580 mg, 1.8 mmol) and ^{t}BuOK (300 mg, 1.98 mmol) were mixed in a J. Young flash and suspended, under argon at –30 °C, in 6 mL of dry THF. The mixture was stirred for 1h at room

temperature. Thereafter, the mixture was cooled to –30 ^{o}C and a solution of complex $[Pt(CH_3)_2(cod)]$ (300 mg, 0.9 mmol) in 3 mL of THF was added slowly via canula. The mixture was left to reach room temperature and stirred for 2h. The solvent was removed under vacuum and the residue was suspended in 10 mL of pentane. Pentane was then evaporated and extracted with 10 + 5 mL of toluene, filtering it via canula. The solvent was removed under vacuum leading to a viscous oil. Pentane (6 mL) was added, and the suspension was vigorously stirred until a white precipitate appears. The solid was filtered off leading to a white solid (360 mg, 65%). **^{1}H NMR** (500 MHz, C_6D_6, 298 K): δ 6.83 and 6.61 (d, $^{3}J_{H,H}$ = 2.2 Hz, 2H each, =CH), 6.22 (d, $^{2}J_{H,H}$ = 13.9 Hz, 2H, $C\underline{\boldsymbol{H}}_2C(CH_3)_3$), 3.69 (d, $^{2}J_{H,H}$ = 13.9 Hz, 2H, $C\underline{\boldsymbol{H}}_2C(CH_3)_3$), 1.59 (s, 18H, $C(CH_3)_3$), 1.13 (s, 18H, $CH_2C(C\underline{\boldsymbol{H}}_3)_3$), 0.76 (s+d, $^{2}J_{Pt,H}$ = 65 Hz, 6H, Pt-CH_3). **^{13}C{^{1}H} NMR** (126 MHz, C_6D_6, 298 K): δ 190.6 (s+d, $J_{Pt,C}$ = 853 Hz, Pt=C), 117.4 (s+d, $^{4}J_{Pt,C}$ = 20 Hz, =CH), 116.6 (s+d, $^{4}J_{Pt,C}$ = 26 Hz, =CH), 62.5 ($\underline{\boldsymbol{C}}H_2C(CH_3)_3$), 57.1 ($\underline{\boldsymbol{C}}(CH_3)_3$), 31.8 ($CH_2\underline{\boldsymbol{C}}(CH_3)_3$), 29.8 ($C(\underline{\boldsymbol{C}}H_3)_3$), 29.2 ($CH_2C(\underline{\boldsymbol{C}}H_3)_3$). Elem. Anal. Calcd. for $C_{26}H_{50}N_4Pt$: C, 50.88; H, 8.21; N, 9.13. Found: C, 51.07; H, 8.04; N, 9.33.

4. Synthesis and characterization of $[Pt(I^{t}Bu^{n}Pe')(I^{t}Bu^{n}Pe)][BAr^{F}]$, 1.3

Complex $[Pt(CH_3)_2(I^{t}Bu^{n}Pe)_2]$ (181 mg, 0.29 mmol) and $H(Et_2O)_2{\cdot}BAr^{F}$ (300 mg, 0.29 mmol) were dissolved in 6 mL of CH_2Cl_2, under argon, at –78°C. After 5 minutes, the reaction mixture was allowed to reach the room temperature and was stirred for 1h. The solvent was evaporated under vacuum. The resulting yellow solid was washed twice with pentane (2 x 5 mL), re-dissolved in CH_2Cl_2 (5 mL) and evaporated under vacuum in order to remove the remaining diethyl ether. This last process was repeated twice leading to **1.3** as a pale-yellow solid which was dried under vacuum for 2h (328 mg, 77%). **^{1}H NMR** (400 MHz, CD_2Cl_2, 298 K): δ 7.73 (br, 8H, H_{ortho}-BAr^{F}), 7.57 (br, 4H, H_{para}-BAr^{F}), 7.16 and 7.02 (d, $^{3}J_{H,H}$ = 2.1 Hz, 1H each, =CH), 6.98 and 6.96 (d, $^{3}J_{H,H}$ = 2.1 Hz, 1H each, =CH), 4.17 (br s, 2H, $C\underline{\boldsymbol{H}}_2C(CH_3)_3$), 3.73 (br s, 2H, $C\underline{\boldsymbol{H}}_2C(CH_3)_3$), 2.90 (s+d, $J_{Pt,H}$ = 108 Hz, 2H, Pt-CH_2), 1.74 (s, 9H, $C(CH_3)_3$), 1.50 (s, 6H, 2 CH_3), 1.00 (s, 18H, $CH_2C(C\underline{\boldsymbol{H}}_3)_3$). **^{13}C{^{1}H} NMR** (100 MHz, CD_2Cl_2, 298 K): δ 178.3 and 171.6 (Pt=C), 162.2 (q, $J_{C,B}$ = 50 Hz, C_{ipso}-BAr^{F}), 135.2 (br, C_{ortho}-BAr^{F}), 129.2 (br q, $J_{C,F}$ = 31 Hz, C_{meta}-BAr^{F}, Ph), 125.0 (q, $J_{C,F}$ = 272 Hz, CF_3-BAr^{F}), 122.9 (s+d, $^{4}J_{Pt,C}$ = 24 Hz, =CH), 122.3 (s, =CH), 119.2 (s+d, $^{4}J_{Pt,C}$ = 32 Hz, =CH), 117.5 (br, C_{para}-BAr^{F}),115.5 (s+d, $^{4}J_{Pt,C}$ = 55 Hz, =CH), 65.5 ($\underline{\boldsymbol{C}}(CH_3)_3$), 62.3 ($\underline{\boldsymbol{C}}H_2C(CH_3)_3$), 61.4 ($\underline{\boldsymbol{C}}H_2C(CH_3)_3$), 59.2 ($\underline{\boldsymbol{C}}(CH_3)_3$), 33.1 ($CH_2\underline{\boldsymbol{C}}(CH_3)_3$), 33.0 ($CH_2\underline{\boldsymbol{C}}(CH_3)_3$), 31.6 ($C(\underline{\boldsymbol{C}}H_3)_3$), 29.9 (s+d, $^{3}J_{Pt,C}$ = 50 Hz, 2 CH_3), 28.3 ($CH_2C(\underline{\boldsymbol{C}}H_3)_3$), 28.0 ($CH_2C(\underline{\boldsymbol{C}}H_3)_3$), 21.9 (br s, Pt-$CH_2$). Elem. Anal. Calcd. for $C_{56}H_{55}BF_{24}N_4Pt$: C, 46.52; H, 3.83; N, 3.87. Found: C, 46.56; H, 3.96; N, 4.06.

5. Synthesis and characterization of germyl complexes [Pt(GeR_3)(I^{t}BuMe)$_2$][BAr^F] (R = Et (2.1a), Ph (2.1b)).

Complex [Pt(I^{t}BuMe)(I^{t}BuMe)][BAr^F] (0.112 mmol) was dissolved in 5 mL of CH_2Cl_2 under argon and, thereafter, the corresponding germane was added (0.168 mmol). The reaction mixture is stirred at room temperature for 18h (**2.1a**) or 30 min (**2.1b**) and the solvent was evaporated under vacuum. The resulting orange solid was washed twice with 5 mL of pentane, and dried under vacuum (**2.1a**, 136 mg, 81%; **2.1b,** 164 mg, 89%).

Complex [Pt($GeEt_3$)(I^{t}BuMe)$_2$][BAr^F], 2.1a: ^{1}H NMR (400 MHz, CD_2Cl_2, 298 K): δ 7.74 (br, 8H, H_{ortho}-BAr^F), 7.57 (br, 4H, H_{para}-BAr^F), 7.17 and 6.95 ($^3J_{H,H}$ = 2.1 Hz, 2H each =CH), 4.03 (s, 6H, N-CH_3), 1.93 (s, 18H, C(CH_3)$_3$), 0.93 (q, $J_{H,H}$ = 7.8 Hz, 6H, C$\underline{\boldsymbol{H}}_2$$CH_3$), 0.72 (t, $J_{H\text{-}H}$ = 7.8 Hz, 9H, CH_2C$\underline{\boldsymbol{H}}_3$). **^{13}C{^{1}H} NMR** (100 MHz, CD_2Cl_2, 298 K): δ 180.9 (Pt=C), 162.2 (q, $J_{C,B}$ = 50 Hz, C_{ipso}-BAr^F), 135.3 (br, C_{ortho}-BAr^F), 129.4 (br, q $J_{C,F}$ = 32 Hz, C_{meta}-BAr^F), 125.0 (q, $J_{C,F}$ = 272 Hz, CF_3-BAr^F), 121.3 (s+d, $^3J_{Pt,C}$ = 35 Hz, =CH), 117.9 (br, C_{para}-BAr^F), 120.4 (s+d, $^3J_{Pt,C}$ = 35 Hz, =CH), 59.8 ($\underline{\boldsymbol{C}}$($CH_3$)$_3$), 40.2 (N-$CH_3$), 33.1 (C($\underline{\boldsymbol{C}}H_3$)$_3$), 11.4 (s+d, $^2J_{Pt,C}$ = 118.7 Hz, Ge-$\underline{\boldsymbol{C}}H_2CH_3$), 9.40 (s, Ge-$CH_2$$\underline{\boldsymbol{C}}H_3$). Elem. Anal. Calcd. for $C_{54}H_{55}BF_{24}GeN_4Pt$: C, 43.40; H, 3.71; N, 3.75. Found: C, 43.64; H, 3.51; N, 3.43.

Complex [Pt($GePh_3$)(I^{t}BuMe)$_2$][BAr^F], 2.1b: ^{1}H NMR (300 MHz, CD_2Cl_2, 298 K): δ 7.72 (br, 8H, H_{ortho}-BAr^F), 7.56 (br, 4H, H_{para}-BAr^F), 7.30 (m, 3H, Ph), 7.19 (m, 12H, Ph), 7.13 and 6.67 ($^3J_{H,H}$ = 2.1 Hz, 2H each =CH), 3.33 (s, 6H, N-CH_3), 1.81 (s, 18H, C(CH_3)$_3$). **^{13}C{^{1}H} NMR** (100 MHz, CD_2Cl_2, 298 K): δ 174.7 (Pt=C), 162.2 (q, $J_{C,B}$ = 50 Hz, C_{ipso}-BAr^F), 139.2 (Ph), 135.3 (br, C_{ortho}-BAr^F), 135.1 (Ph), 129.4–128.9 (m, C_{meta}-BAr^F and Ph), 128.4 (Ph), 125.0 (q, $J_{C,F}$ = 272 Hz, CF_3-BAr^F), 122.2 and 120.7 (=CH), 117.9 (br, C_{para}-BAr^F), 59.7 ($\underline{\boldsymbol{C}}$($CH_3$)$_3$), 39.9 (N-$CH_3$), 33.4 (C($\underline{\boldsymbol{C}}H_3$)$_3$). Elem. Anal. Calcd. for $C_{66}H_{55}BF_{24}GeN_4Pt$: C, 48.38; H, 3.38; N, 3.41. Found: C, 48.59; H, 3.58; N, 3.38.

6. Synthesis and characterization of germyl complexes [Pt(GeR_2R')(I^{t}Bu^{i}Pr)$_2$][BAr^F] (R = R' = Et (2.2a), Ph (2.2b); R = H, R' = ^{n}Bu (2.2c); R = Ph, R' =H (2.2d)).

Complex [Pt(I^{t}Bu^{i}Pr')(I^{t}Bu^{i}Pr)][BAr^F], **1.2**, (**2.2a** and **2.2b,** 0.112 mmol; **2.2c**, 0.057 mmol; **2.2d**, 0.036 mmol) was dissolved in CH_2Cl_2 (5 mL) under argon and, thereafter, the corresponding germane was added (**2.2a** and **2.2b**, 0.168 mmol; **2.2c**, 0.063 mmol; **2.2d**, 0.036 mmol). Further stirring for 18h (**2.2a**) or 30 min (**2.2b**, **2.2c** and **2.2d**), followed by evaporation of the solvent and washing with dry pentane (2 x 5 mL) yielded complexes **2.2** as orange solids (**2.2a**, 156 mg, 90%; **2.2b**, 176 mg, 92%; **2.2c**, 55 mg,

57%; **2.2d**, 40 mg, 69%). Complex **2.2d** contains ca. 5% of an imidazolium salt that precluded its isolation in pure form.

Complex [Pt(GeEt$_3$)(I^{t}Bu^{i}Pr)$_2$][BAr^F], 2.2a: ^{1}H NMR (500 MHz, CD_2Cl_2, 298 K): δ 7.74 (br, 8H, H_{ortho}-BAr^F), 7.57 (br, 4H, H_{para}-BAr^F), 7.20 and 7.05 ($^3J_{H\text{-}H}$ = 2.2 Hz, 2H each =CH), 5.48 (sept, $^3J_{H,H}$ = 6.8 Hz, 2H, C***H***(CH$_3$)$_2$), 1.94 (s, 18H, C(CH$_3$)$_3$), 1.52 (d, $^3J_{H,H}$ = 6.8 Hz, 12H, CH(C***H***$_3$)$_2$), 0.94 (q, $^3J_{H,H}$ = 7.8 Hz, 6H, C***H***$_2$CH$_3$), 0.73 (t, $^3J_{H,H}$ = 7.8 Hz, 9H, CH$_2$C***H***$_3$). **^{13}C{^{1}H} NMR** (126 MHz, CD_2Cl_2, K): δ 179.7 (Pt=C), 162.2 (q, $J_{C,B}$ = 50 Hz, C_{ipso}-BAr^F), 135.2 (br, C_{ortho}-BAr^F), 129.3 (br, q $J_{C,F}$ = 31 Hz, C_{meta}-BAr^F), 125.0 (q, $J_{C,F}$ = 272 Hz, CF$_3$-BAr^F), 120.8 (s+d, $^3J_{Pt,C}$ = 31 Hz, =CH), 117.9 (br, C_{para}-BAr^F), 115.8 (s+d, $^3J_{Pt,C}$ = 31 Hz, =CH), 59.6 (***C***(CH$_3$)$_3$), 53.8 (***C***H(CH$_3$)$_2$), 33.4 (C(***C***H$_3$)$_3$), 23.9 (CH(***C***H$_3$)$_2$), 11.5 (s+d, $^2J_{Pt,C}$ = 118.5 Hz, Ge-***C***H$_2$CH$_3$), 9.40 (s, Ge-CH$_2$***C***H$_3$). Elem. Anal. Calcd. for $C_{58}H_{63}BF_{24}GeN_4Pt$: C, 44.93; H, 4.10; N, 3.61. Found: C, 44.83; H, 4.32; N, 3.68.

Complex [Pt(GePh$_3$)(I^{t}Bu^{i}Pr)$_2$][BAr^F] 2.2b: ^{1}H NMR (400 MHz, CD_2Cl_2, 298 K): δ 7.73 (br, 8H, H_{ortho}-BAr^F), 7.57 (br, 4H, H_{para}-BAr^F), 7.28 (m, 3H, Ph), 7.23–7.20 (m, 14H, Ph, =CH), 6.90 (d, $^3J_{H,H}$ = 2.2 Hz, 2H, =CH), 5.06 (sept, $^3J_{H,H}$ = 6.7 Hz, 2H, C***H***(CH$_3$)$_2$), 1.79 (s, 18H, C(CH$_3$)$_3$), 0.89 (d, $^3J_{H,H}$ = 6.7 Hz, 12H, CH(C***H***$_3$)$_2$). **^{13}C{^{1}H} NMR** (100 MHz, CD_2Cl_2, 298 K): δ 175.9 (Pt=C), 162.2 (q, $J_{C,B}$ = 50 Hz, C_{ipso}-BAr^F), 139.5 (C_{ipso}-Ph), 135.2 (br, C_{ortho}-BAr^F), 134.3 (Ph), 129.8–129.1 (m, C_{meta}-BAr^F, Ph), 128.9 (Ph), 125.0 (q, $J_{C,F}$ = 272 Hz, CF$_3$-BAr^F), 121.2 (=CH), 117.5 (br, C_{para}-BAr^F), 116.3 (=CH), 59.7 (***C***(CH$_3$)$_3$), 54.8 (***C***H(CH$_3$)$_2$), 33.7 (C(***C***H$_3$)$_3$), 22.9 (CH(***C***H$_3$)$_2$). Elem. Anal. Calcd. for $C_{70}H_{63}BF_{24}GeN_4Pt$: C, 49.61; H, 3.75; N, 3.31. Found: C, 49.60; H, 3.73; N, 3.25 .

Complex [Pt(GeH$_2$^{n}Bu)(I^{t}Bu^{i}Pr)$_2$][BAr^F], 2.2c: ^{1}H NMR (400 MHz, CD_2Cl_2, 298 K): δ 7.73 (br, 8H, H_{ortho}-BAr^F), 7.56 (br, 4H, H_{para}-BAr^F), 7.19 and 7.05 (s, 2H each =CH), 5.65 (m, 2H, C***H***(CH$_3$)$_2$), 2.59 (s+d, $^3J_{Pt,H}$ = 188 Hz, 2H, Ge*H$_2$*) 1.90 (s, 18H, C(CH$_3$)$_3$), 1.50 (d, $^3J_{H,H}$ = 8.0 Hz, 12H, CH(C***H***$_3$)$_2$), 1.20 (m, 4H, Ge-(CH$_2$)$_3$CH$_3$), 0.76 (m, 5H, Ge-(CH$_2$)$_3$CH$_3$). **^{13}C{^{1}H} NMR** (100 MHz, CD_2Cl_2, 298 K): δ 177.0 (Pt=C), 162.2 (q, $J_{C,B}$ = 50 Hz, C_{ipso}-BAr^F), 135.2 (br, C_{ortho}-BAr^F), 129.3 (br, q $J_{C,F}$ = 31.4 Hz, C_{meta}-BAr^F), 125.0 (q, $J_{C,F}$ = 272 Hz, CF$_3$-BAr^F), 120.9 (=CH), 117.9 (br, C_{para}-BAr^F), 116.2 (=CH), 59.4 (***C***(CH$_3$)$_3$), 54.5 (***C***H(CH$_3$)$_2$), 32.9 (C(***C***H$_3$)$_3$), 23.5 (CH(***C***H$_3$)$_2$), 29.9, 25.4, 16.4 and 13.7 (Ge-(CH$_2$)$_3$CH$_3$). Elem. Anal. Calcd. for $C_{56}H_{59}BF_{24}GeN_4Pt$: C, 44.18; H, 3.91; N, 3.68. Found: C, 44.44; H, 3.86; N, 3.69.

Complex [Pt(GeHPh$_2$)(I^{t}Bu^{i}Pr)$_2$][BAr^F], 2.2d: ^{1}H NMR (500 MHz, CD_2Cl_2, 298 K): δ 7.77 (br, 8H, H_{ortho}-BAr^F), 7.60 (br, 4H, H_{para}-BAr^F), 7.30 (m, 2H, Ph_{para}), 7.21–7.19 (m,

6H, Ph_{para} and =CH), 7.05 (d, $^3J_{H,H}$ = 7.3 Hz, 4H, Ph_{ortho}), 6.96 (s, 2H, =CH), 5.33 (m, 2H, C***H***$(CH_3)_2$), 4.28 (s+d, $^3J_{Pt,H}$ = 240 Hz, 1H, GeH) 1.83 (s, 18H, $C(CH_3)_3$), 1.23 (d, $^3J_{H,H}$ = 6.8 Hz, 12H, CH(C***H***$_3$)$_2$). **$^{13}C\{^1H\}$ NMR** (126 MHz, CD_2Cl_2, 298 K): δ 176.8 (Pt=C), 162.2 (q, $J_{C,B}$ = 50 Hz, C_{ipso}-BAr^F), 137.7 (Ph_{ipso}), 135.2 (br, C_{ortho}-BAr^F), 134.1 (CH-Ph_{ortho}), 129.6 (CH-Ph_{para}), 129.3 (br, q $J_{C,F}$ = 31.4 Hz, C_{meta}-BAr^F), 128.8 (CH-Ph_{meta}), 125.0 (q, $J_{C,F}$ = 272 Hz, CF_3-BAr^F), 121.0 (s, =CH), 117.9 (br, C_{para}-BAr^F), 116.3 (s, =CH), 59.6 (***C***$(CH_3)_3$), 54.6 (***C***$H(CH_3)_2$), 33.4 (C(***C***$H_3)_3$), 23.4 (CH(***C***$H_3)_2$).

7. Synthesis and characterization of germyl complexes [Pt(GeR$_2$R')(I^{t}Bu^{n}Pe)$_2$][BAr^F] (R = R' = Et (2.3a), Ph (2.3b); R = H, R' = ^{n}Bu (2.3c); R = Ph, R' = H (2.3d)).

Complex [Pt(I^{t}Bu^{n}Pe)(I^{t}Bu^{n}Pe)][BAr^F] (0.024 mmol, **2.3a**; 0.067 mmol, **2.3b**; 0.055 mml, **2.3d**) was dissolved in 0.6 mL of CD_2Cl_2 under argon and, thereafter, the corresponding germane was added (0.040 mmol, **2.3a**; 0.100 mmol, **2.3b**; 0.055 mmol, **2.3d**). The reaction mixture is stirred at room temperature for 18h min (**2.3a**), 30 min (**2.3b**) or 15 min (**2.3d**) and the solvent was evaporated under vacuum. The resulting orange solid was washed twice with 3 mL of pentane, and dried under vacuum (**2.3a,** 20 mg, 51%; **2.3b**, 99 mg, 82%; **2.3d**, 38 mg, 41%). On the other hand, complex **2.3c** was synthesized using an alternative route because the direct synthesis between **1.3** and the corresponding germane has given rise a mixture of compounds. In this case, hydride complex **4** (0.060 mmol), generated *in situ* by pressurizing complex **1.3** with 2 bar of H_2 in a J-valved NMR in 0.4 mL of CD_2Cl_2 (see below) was treated with 1 equivalent of Ge^{n}BuH$_3$ (0.060 mmol) in CD_2Cl_2 (0.2 mL) and the corresponding reaction mixture was stirred for 30 min. Thereafter, the solvent was evaporated under vacuum giving rise an orange solid which was washed with pentane twice leading to **2.3c** as a pure solid (56 mg, 65 %).

Complex [Pt(GeEt$_3$)(I^{t}Bu^{n}Pe)$_2$][BAr^F], 2.3a: ^{1}H NMR (400 MHz, CD_2Cl_2, 298 K): δ 7.73 (br, 8H, H_{ortho}-BAr^F), 7.57 (br, 4H, H_{para}-BAr^F), 7.21 and 7.07 (d, $^3J_{H,H}$ = 2.1 Hz, 2H each =CH), 4.31 (br s, 4H, C***H***$_2C(CH_3)_3$), 1.98 (s, 18H, $C(CH_3)_3$), 1.03 (s, 18H, CH_2C (C***H***$_3)_2$), 0.78 (q, $J_{H,H}$ = 7.8 Hz, 6H, C***H***$_2CH_3$), 0.67 (t, $J_{H,H}$ = 7.8 Hz, 9H, CH_2C***H***$_3$). **$^{13}C\{^1H\}$ NMR** (100 MHz, CD_2Cl_2, 298 K): δ 181.5 (Pt=C), 162.2 (q, $J_{C,B}$ = 50 Hz, C_{ipso}-BAr^F), 135.2 (br, C_{ortho}-BAr^F), 129.3 (br q, $J_{C,F}$ = 31.4 Hz, C_{meta}-BAr^F), 125.0 (q, $J_{C,F}$ = 272 Hz, CF_3-BAr^F), 120.4 and 120.1 (=CH), 117.9 (br, C_{para}-BAr^F), 64.3 (***C***$H_2C(CH_3)_3$), 59.7 (***C***$(CH_3)_2$), 33.9 (C(***C***$H_3)_3$), 33.5 (CH_2***C***$(CH_3)_3$), 28.2 (CH_2C(***C***$H_3)_3$), 11.0 (s+d, $^2J_{Pt,C}$ = 155.0 Hz, Ge-***C***H_2CH_3), 9.29 (s, Ge-CH_2***C***H_3). Elem. Anal. Calcd. for $C_{62}H_{71}BF_{24}GeN_4Pt$: C, 46.35; H, 4.45; N, 3.49. Found: C, 46.48; H, 4.44; N, 3.28.

Complex [Pt(GePh$_3$)(I^{t}Bu^{n}Pe)$_2$][BAr^F], 2.3b: ^{1}H NMR (400 MHz, CD_2Cl_2, 298 K): δ 7.74 (br, 8H, H$_{ortho}$-BAr^F), 7.58 (br, 4H, H$_{para}$-BAr^F), 7.31 (t, $^3J_{H,H}$ = 7.4 Hz, 3H, Ph), 7.27–7.17 (m, 9H, Ph), 7.14 (br s, 3H, Ph), 7.13 (br s, 2H, =CH), 6.88 (d, $^3J_{H,H}$ = 2.1 Hz, 2H, =CH), 3.39 (C***H***$_2$C(CH$_3$)$_3$), 1.82 (s, 18H, C(CH$_3$)$_3$), 0.83 (s, 18H, CH$_2$C(C***H***$_3$)$_3$). **^{13}C{^{1}H} NMR** (100 MHz, CD_2Cl_2, 298 K): δ 177.5 (Pt=C), 162.2 (q, $J_{C,B}$ = 50 Hz, C$_{ipso}$-BAr^F), 139.0 (Ph$_{ipso}$), 135.2 (br, q $J_{C,F}$ = 32 Hz, C$_{meta}$-BAr^F), 134.3 (Ph), 129.3 (Ph), 128.8 (Ph), 125.0 (q, $J_{C,F}$ = 272 Hz, CF$_3$-BAr^F), 120.8–120.7 (br, 2 =CH), 117.9 (br, C$_{para}$-BAr^F), 63.7 (***C***H$_2$C(CH$_3$)$_3$), 59.8 (***C***(CH$_3$)$_3$), 34.2 (C(***C***H$_3$)$_3$), 33.2 (CH$_2$***C***(CH$_3$)$_3$), 28.0 (CH$_2$C(***C***H$_3$)$_3$). Elem. Anal. Calcd. for $C_{74}H_{71}BF_{24}GeN_4Pt$: C, 50.76; H, 4.09; N, 3.20. Found: C, 51.22; H, 4.27; N, 3.21.

Complex [Pt(GeH$_2$^{n}Bu)(I^{t}Bu^{n}Pe)$_2$][BAr^F], 2.3c: ^{1}H NMR (400 MHz, CD_2Cl_2, 298 K): δ 7.75 (br, 8H, H$_{ortho}$-BAr^F), 7.58 (br, 4H, H$_{para}$-BAr^F), 7.21 and 7.07 (d, $^3J_{H,H}$ = 2.1 Hz, 2H each =CH), 4.32 (br s, 4H, C***H***$_2$C(CH$_3$)$_3$), 2.47 (s+d, $J_{Pt,H}$ = 188 Hz, 2H, GeH$_2$) 1.96 (s, 18H, C(CH$_3$)$_3$), 1.05 (s, 18H, CH$_2$C(C***H***$_3$)$_3$), 1.22–1.08 (m, 4H, CH$_3$(C***H***$_2$)$_2$CH$_2$Ge), 0.77 (t, $^3J_{H,H}$ = 7.1 Hz, 3H, C***H***$_3$(CH$_2$)$_3$Ge), 0.68 (m, 2H, GeCH$_2$). **^{13}C{^{1}H} NMR** (100 MHz, CD_2Cl_2, 298 K): δ 179.6 (Pt=C), 162.2 (q, $J_{C,B}$ = 50 Hz, C$_{ipso}$-BAr^F), 135.2 (br, C$_{ortho}$-BAr^F), 129.3 (br q, $J_{C,F}$ = 31.4 Hz, C$_{meta}$-BAr^F), 125.0 (q, $J_{C,F}$ = 272 Hz, CF$_3$-BAr^F), 121.0 and 120.1 (=CH), 117.9 (br, C$_{para}$-BAr^F), 63.8 (***C***H$_2$C(CH$_3$)$_3$), 59.5 (***C***(CH$_3$)$_2$), 33.6 (C(***C***H$_3$)$_3$), 33.4 (CH$_2$***C***(CH$_3$)$_3$), 29.4 (CH$_2$), 28.2 (CH$_2$C(***C***H$_3$)$_3$), 25.3 (CH$_2$), 16.1 (CH$_2$), 13.7 (CH$_2$***C***H$_3$). Elem. Anal. Calcd. for $C_{60}H_{67}BF_{24}GeN_4Pt$: C, 45.65; H, 4.28; N, 3.55. Found: C, 45.60; H, 4.51; N, 3.64.

Complex [Pt(GeHPh$_2$)(I^{t}Bu^{n}Pe)$_2$][BAr^F], 2.3d: ^{1}H NMR (500 MHz, CD_2Cl_2, 298 K): δ 7.76 (br, 8H, H$_{ortho}$-BAr^F), 7.60 (br, 4H, H$_{para}$-BAr^F), 7.33 (t, $^3J_{H,H}$ = 7.5 Hz, 2H, Ph$_{para}$), 7.20 (t, $^3J_{H,H}$ = 7.4 Hz, 4H, Ph$_{meta}$), 7.16 ($^3J_{H-H}$ = 2.1 Hz, 2H, =CH), 7.08 (d, $^3J_{H,H}$ = 7.4 Hz, 4H, Ph$_{ortho}$), 6.82 ($^3J_{H-H}$ = 2.1 Hz, 2H, =CH), 4.07 (s+d, $^2J_{Pt,H}$ = 230 Hz, 1H, GeH), 3.77 (br s, 4H, C***H***$_2$C(CH$_3$)$_3$), 1.90 (s, 18H, C(CH$_3$)$_3$), 0.93 (s, 18H, CH$_2$C(C***H***$_3$)$_3$). **^{13}C{^{1}H} NMR** (126 MHz, CD_2Cl_2, 298 K): δ 178.3 (Pt=C), 162.2 (q, $J_{C,B}$ = 50 Hz, C$_{ipso}$-BAr^F), 137.5 (Ph$_{ipso}$), 135.2 (br, C$_{ortho}$-BAr^F), 134.0 (CH-Ph$_{ortho}$), 129.6 (CH-Ph$_{para}$), 129.3 (br q, $J_{C,F}$ = 31.4 Hz, C$_{meta}$-BAr^F), 128.7 (CH-Ph$_{meta}$), 125.0 (q, $J_{C,F}$ = 272 Hz, CF$_3$-BAr^F), 121.0 (=CH), 120.3 (=CH), 117.9 (br, C$_{para}$-BAr^F), 63.9 (***C***H$_2$C(CH$_3$)$_3$), 59.6 (***C***(CH$_3$)$_2$), 34.0 (C(***C***H$_3$)$_3$), 33.3 (CH$_2$***C***(CH$_3$)$_3$), 28.0 (CH$_2$C(***C***H$_3$)$_3$). Elem. Anal. Calcd. for $C_{68}H_{67}BF_{24}GeN_4Pt$: C, 48.77; H, 4.03; N, 3.35. Found: C, 48.98; H, 3.79; N, 3.26.

8. Synthesis and characterization of germyl complexes $[Pt(GeH_2{}^nBu)(IMes^*)_2][BAr^F]$, 2.4c, and $[Pt(GeHPh_2)(IMes^*)_2][BAr^F]$, 2.4d.

Complex $[Pt(I^*Mes^{*\prime})(I^*Mes^*))][BAr^F]$ (0.060 mmol) was dissolved in 3 mL of CH_2Cl_2 under argon and, thereafter, the corresponding germane was added (0.052 mmol). The reaction mixture is stirred at room temperature for 18h and the solvent was evaporated under vacuum. The resulting orange solid was washed twice with 3 mL of pentane, and dried under vacuum (**2.4c**, 50 mg, 77%; **2.4d,** 48 mg, 74%;).

Complex $[Pt(GeH_2{}^nBu)(IMes^*)_2][BAr^F]$, 2.4c: ^{1}H NMR (500 MHz, CD_2Cl_2, 298K): δ 7.74 (br, 8H, H_{ortho}-BAr^F), 7.57 (br, 4H, H_{para}-BAr^F), 6.96 (s, 8H, CH-Mes), 2.42 (s, 12H, CH_3), 1.86–1.64 (br, 38H, CH_3 and GeH_2), 1.14 (m, 2H, $CH_3C\underline{H}_2$), 0.97 (m, 2H, $CH_2C\underline{H}_2CH_2$), 0.80 (m, 3H, $C\underline{H}_3(CH_2)_3Ge$), 0.40 (m, 2H, $CH_3(CH_2)_2C\underline{H}_2Ge$). **^{13}C{^{1}H} NMR** (126 MHz, CD_2Cl_2, 298 K): δ 181.2 (Pt=C), 162.2 (q, $J_{C,B}$ = 50 Hz, C_{ipso}-BAr^F), 139.9 (C_q-Mes), 135.6 (C_q-Mes), 135.2 (br, C_{ortho}-BAr^F), 134.0 (C_q-Mes), 129.9 (CH-Mes), 129.4 (br, q $J_{C,F}$ = 32 Hz, C_{meta}-BAr^F), 127.2 (=$\underline{C}CH_3$), 125.0 (q, $J_{C,F}$ = 272 Hz, CF_3-BAr^F), 117.9 (br, C_{para}-BAr^F), 29.4 ($\underline{C}H_2$), 25.6 ($\underline{C}H_2$), 21.3 ($\underline{C}H_3$), 18.4 ($\underline{C}H_3$), 17.7 ($\underline{C}H_2Ge$), 13.7 ($CH_2\underline{C}H_3$), 9.16 (=$C\underline{C}H_3$). Elem. Anal. Calcd. for $C_{82}H_{79}BF_{24}GeN_4Pt$: C, 53.10; H, 4.30; N, 3.02. Found: C, 53.16; H, 4.57; N, 3.22.

Complex $[Pt(GeHPh_2)(IMes^*)_2][BAr^F]$, 2.4d: ^{1}H NMR (500 MHz, CD_2Cl_2, 298 K): δ 7.74 (br, 8H, H_{ortho}-BAr^F), 7.57 (br, 4H, H_{para}-BAr^F), 7.25 (t, $^{\cdot 3}J_{H,H}$ = 7.4 Hz, 2H, Ph_{para}), 6.99 (t, $^3J_{H,H}$ = 7.4 Hz, 4H, Ph_{meta}), 6.85 (s, 8H, CH-Mes), 6.72 (d, $^3J_{H,H}$ = 7.4 Hz, 4H, Ph_{ortho}), 3.45 (s+d, $^2J_{Pt,H}$ = 215 Hz, 1H, GeH), 2.47 (s, 12H, CH_3), 1.64 (s, 24H, CH_3), 1.61 (s, 12H, CH_3) . **^{13}C{^{1}H} NMR** (126 MHz, CD_2Cl_2, 298K): δ 178.6 (Pt=C), 162.2 (q, $J_{C,B}$ = 50 Hz, C_{ipso}-BAr^F), 139.6 (C_q-Mes), 136.7 (Ph_{ipso}), 135.6 (C_q-Mes), 135.2 (br, C_{ortho}-BAr^F), 134.9 (CH-Ph_{ortho}), 134.0 (C_q-Mes), 130.2 (CH-Mes), 129.4 (br, q $J_{C,F}$ = 32 Hz, C_{meta}-BAr^F), 128.9 (CH-Ph_{para}), 128.2 (=$\underline{C}CH_3$), 128.0 (CH-Ph_{meta}), 125.0 (q, $J_{C,F}$ = 272 Hz, CF_3-BAr^F), 117.9 (br, C_{para}-BAr^F), 21.5 (CH_3), 18.9 (CH_3), 9.35 (=$C\underline{C}H_3$). Elem. Anal. Calcd. for $C_{90}H_{79}BF_{24}GeN_4Pt$: C, 55.41; H, 4.08; N, 2.87. Found: C, 55.33; H, 4.44; N, 2.60.

9. Low temperature studies NMR studies. Characterization of complexes 1.2·$HGeEt_3$, 1.2·$HGePh_3$ and 1.2·$HGeH_2{}^nBu$

$[Pt(I^tBu^iPr')(I^tBu^iPr)(HGeEt_3)][BAr^F]$, 1.2·$HGeEt_3$

Complex $[Pt(I^tBu^iPr')(I^tBu^iPr)][BAr^F]$ (80 mg, 0.057 mmol) was dissolved in 0.6 mL of CD_2Cl_2 in a screw-cap NMR tube. The reaction mixture was cooled to –78 ºC and $GeHEt_3$ (10 µL, 0.063 mmol) was injected. The sample was analyzed by NMR in a

precooled (213 K) NMR apparatus. **^{1}H NMR** (400 MHz, CD_2Cl_2, 213 K): δ 7.79 (br, 8H, H_{ortho}-BAr^F), 7.59 (br, 4H, H_{para}-BAr^F), 7.20 (d, $^3J_{H,H}$ = 2.2 Hz, 1H, =CH), 7.04–6.99 (m, 3H, =CH), 5.37 (m, 1H, C***H***(CH_3)$_2$), 4.33 (m, 1H, C***H***(CH_3)$_2$), 2.23 (d, $^2J_{H,H}$ = 11.9 Hz, 1H, Pt-C***H**$_a$*H$_b$), 2.06 (d, $^2J_{H,H}$ = 11.9 Hz, 1H, Pt-CH$_a$***H**$_b$*), 1.65 (s, 9H, C(CH_3)$_3$), 1.51–1.47 (m, 9H, CH(C***H***$_3$)$_2$ and CH_3), 1.42 (m, 6H, CH(C***H***$_3$)$_2$), 1.34 (br s, 3H, CH_3), 1.16–0.98 (m, 6H, $GeEt_3$), 0.91 (m, 9H, $GeEt_3$), -5.36 (s+d, $^1J_{Pt,H}$ = 400 Hz, Pt-H-Ge). **^{13}C{^{1}H} NMR** (100 MHz, CD_2Cl_2, 213 K): δ 168.6 and 166.6 (Pt=C), 161.9 (q, $J_{C,B}$ = 50 Hz, C_{ipso}-BAr^F), 134.7 (br, C_{ortho}-BAr^F), 128.7 (br, q $J_{C,F}$ = 31 Hz, C_{meta}-BAr^F), 125.0 (q, $J_{C,F}$ = 272 Hz, CF_3-BAr^F), 120.9 (=CH), 117.6 (br, C_{para}-BAr^F and =CH), 115.8 and 115.7 (br s, =CH), 65.2 and 58.7 (***C***(CH_3)$_3$), 53.3 and 52.4 (***C***H(CH_3)$_2$), 35.2 (Pt-CH_2), 32.7 (CH(***C***H$_3$)$_2$), 30.8 (C(***C***H$_3$)$_3$), 28.3 (CH_3), 24.9, 24.7, 22.9, 21.6 (CH(***C***H$_3$)$_2$ and CH_3), 9.2 and 8.8 ($GeEt_3$).

[Pt(I^{t}Bu^{i}Pr')(I^{t}Bu^{i}Pr)(HGePh$_3$)][BAr^F], 1.2·HGePh$_3$

Complex [Pt(I^{t}Bu^{i}Pr')(I^{t}Bu^{i}Pr)][BAr^F] (80 mg, 0.057 mmol) and $GeHPh_3$ (19 mg, 0.063 mmol) were dissolved in 0.6 mL of CD_2Cl_2 in a screw-cap NMR tube at –78 ºC. The sample was analyzed by NMR in a precooled (198 K) NMR apparatus. **^{1}H NMR** (400 MHz, CD_2Cl_2, 198 K): δ 7.82 (br, 8H, H_{ortho}-BAr^F), 7.60 (br, 4H, H_{para}-BAr^F), 7.52 (br s, 2H, =CH), 7.42–7.29 (m, 8H, Ph), 7.28 (br s, 3H, Ph), 7.17 and 7.07 (br s, 2H each, Ph), 6.92–6.88 (m, 4H, =CH and Ph), 4.64 (m, 1H, C***H***(CH_3)$_2$), 4.37 (m, 1H, C***H***(CH_3)$_2$), 2.28 (d, $^2J_{H,H}$ = 12.0 Hz, 1H, Pt-C***H**$_a$*H$_b$), 2.03 (d, $^2J_{H,H}$ = 12.0 Hz, 1H, Pt-CH$_a$***H**$_b$*), 1.89 (s, 9H, C(CH_3)$_3$), 1.31–1.09 (m, 9H, CH(C***H***$_3$)$_2$ and CH_3), 0.62, 0.33 and 0.22 (br, 3H each, CH(C***H***$_3$)$_2$ and CH_3), -4.68 (s+d, $^1J_{Pt,H}$ = 396 Hz, Pt-H-Ge). **^{13}C{^{1}H} NMR** (100 MHz, CD_2Cl_2, 198 K): δ 166.5 and 163.8 (Pt=C), 161.9 (q, $J_{C,B}$ = 50 Hz, C_{ipso}-BAr^F), 140.5 (Ph), 136.0 (Ph), 135.3 (Ph), 134.7 (br, C_{ortho}-BAr^F), 134.1–133.7 (m, Ph), 130.1–128.6 (m, C_{meta}-BAr^F and Ph), 125.0 (q, $J_{C,F}$ = 272 Hz, CF_3-BAr^F), 121.4 and 118.1 (=CH), 117.7 (br, C_{para}-BAr^F), 116.6 and 116.4 (=CH), 66.8 and 59.3 (***C***(CH_3)$_3$), 53.0 and 52.9 (***C***H(CH_3)$_2$), 41.9 (br, Pt-CH_2), 31.6 (C(***C***H$_3$)$_3$), 29.1, 28.5, 25.2, 24.9, 21.2 and 19.2 (CH(***C***H$_3$)$_2$ and CH_3).

[Pt(I^{t}Bu^{i}Pr')(I^{t}Bu^{i}Pr)(HGeH$_2$^{n}Bu)][BAr^F], 1.2·HGeH$_2$^{n}Bu

Complex [Pt(I^{t}Bu^{i}Pr')(I^{t}Bu^{i}Pr)][BAr^F] (80 mg, 0.057 mmol) was dissolved in 0.3 mL of CD_2Cl_2 . The solution was cooled to –78 ºC and a solution of ^{n}BuGeH$_3$ were injected. The sample was analyzed by NMR in a pre-cooled (233 K) NMR apparatus. **^{1}H NMR** (400 MHz, CD_2Cl_2, 233 K): δ 7.78 (br, 8H, H_{ortho}-BAr^F), 7.60 (br, 4H, H_{para}-BAr^F), 7.27 (s, 1H each, =CH), 7.07 (m, 3H, =CH), 5.31 (m, 1H, C***H***(CH_3)$_2$), 4.81 (d, $^2J_{H,H}$ = 13.4 Hz,

1H, GeH_2), 4.56 (m, 1H, C***H***(CH_3)$_2$), 4.52 (d, $^2J_{H,H}$ = 13.4 Hz, 1H, GeH_2), 2.40 (d+d, $^2J_{H,H}$ = 13.0 Hz, $^2J_{Pt,H}$ = 64.0 Hz, 1H, Pt-C***H$_a$***H$_b$), 2.11 (d+d, $^2J_{H,H}$ = 13.0 Hz, $^2J_{Pt,H}$ = 92.0 Hz, 1H, Pt-CH$_a$***H$_b$***), 1.67 (s, 9H, C(CH_3)$_3$), 1.54 (br, 3H, CH_3), 1.50–1.40 (m, 15H, CH(C***H***$_3$)$_2$ and CH_3), 1.32, 1.24 and 1.15 (br, 2H each, Ge-(C***H***$_2$)$_3$$CH_3$), 0.79 (br, 3H, Ge-($CH_2$)$_3$C***H***$_3$), -5.59 (s+d, $^1J_{Pt,H}$ = 416 Hz, Pt-H-Ge). **^{13}C{^{1}H} NMR** (100 MHz, CD_2Cl_2, 233 K): δ 165.9 and 164.4 (Pt=C), 161.8 (q, $J_{C,B}$ = 50 Hz, C_{ipso}-BAr^F), 134.7 (br, C_{ortho}-BAr^F), 128.7 (br, q $J_{C,F}$ = 31 Hz, C_{meta}-BAr^F), 125.0 (q, $J_{C,F}$ = 272 Hz, CF_3-BAr^F), 121.1 and 117.8 (=CH), 117.6 (br, C_{para}-BAr^F), 116.7 and 116.2 (=CH), 65.5 and 58.6 (***C***(CH_3)$_3$), 53.3 and 52.9 (***C***H(CH_3)$_2$), 39.6 (s, Pt-CH_2), 32.5 (CH(***C***H$_3$)$_2$), 31.0 (C(***C***H$_3$)$_3$), 29.2 (CH_3), 25.4 (Ge^{n}Bu), 24.2, 24.1, 23.3 and 22.8 (CH(***C***H$_3$)$_2$ and CH_3), 16.5 and 13.6 (Ge^{n}Bu).

10. Reaction of complex [Pt(I^{t}Bu^{n}Pe')(I^{t}Bu^{n}Pe)][BAr^F] with ^{n}BuSiH$_3$

Complex [Pt(I^{t}Bu^{n}Pe)(I^{t}Bu^{n}Pe)][BAr^F] (**1.3**, 0.021 mmol) was dissolved in 0.6 mL of CD_2Cl_2 under argon and, thereafter, the corresponding ^{n}BuGeH$_3$ was added (0.025 mmol). Full conversion of the starting material was observed after 15 min, leading to a mixture of products containing complexes **2.3c** and **3.3c** (in a ca. 2:1 ratio) (see Figure SXXX).

11. Synthesis of hydride complex [Pt(H)(I^{t}Bu^{n}Pe)$_2$][BAr^F]

Complex **1.3** (80 mg, 0.06 mmol) was dissolved in 0.6 mL of CD_2 Cl_2 in a J. Young NMR tube with a screw cap. Thereafter, H_2 was introduced at 2 bar. The initial pale yellow solution became nearly colourless in about 30 min. The ^{1}H NMR spectrum at room temperature indicated the full conversion to complex **4**. This compound has been characterized in solution by ^{1}H NMR.

Complex [Pt(H)(I^{t}Bu^{n}Pe)$_2$][BAr^F], 4: ^{1}H NMR (300 MHz, CD_2Cl_2, 298 K): δ 7.76 (br, 8H, H_{ortho}-BAr^F), 7.59 (br, 4H, H_{para}-BAr^F), 7.13 (d, $^3J_{H\text{-}H}$ = 2.3 Hz, 2H, =CH), 7.01 (d, $^3J_{H,H}$ = 2.3 Hz, 2H, =CH), 4.14 (s, 4H, C***H***$_2$C(CH_3)$_3$), 1.72 (s, 18H, C(CH_3)$_3$), 1.01 (s, 18H, CH_2C(C***H***$_3$)$_3$), –25.5 (s+d, $^1J_{Pt,H}$ = 2560 Hz, 1H, Pt-H).

12. Synthesis and characterization of silyl complexes [Pt(SiHPh$_2$)(I^{t}BuMe)$_2$][BAr^F], 5, and [Pt(SiH$_2$Ph)(I^{t}BuMe)$_2$][BAr^F], 6.

Complex [Pt(GeR$_3$)(I^{t}BuMe)$_2$][BAr^F] (R = Et or Ph) (0.048 mmol) was dissolved in 5 mL of CH_2Cl_2 under argon and, thereafter, the corresponding silane was added (0.098 mmol). The reaction mixture is stirred at room temperature for 1h (R = Ph) or 18h (R = Et) and the solvent was evaporated under vacuum. The resulting orange solid was

washed twice with 4 mL of pentane, and dried under vacuum (**5,** 59 mg, 81%; **6**, 58 mg, 84%).

Complex [Pt(SiHPh$_2$)(I^{t}BuMe)$_2$][BAr^F], 5: ^{1}H NMR (500 MHz, CD_2Cl_2, 298 K): δ 7.75 (br, 8H, H_{ortho}-BAr^F), 7.58 (br, 4H, H_{para}-BAr^F), 7.29 (m, 2 H, Ph), 7.19–7.17 (m, 10H, Ph and =CH), 6.75 (d, $^3J_{H,H}$ = 2.0 Hz, 2H, =CH), 4.81 (s+d+d, $^2J_{H,Pt}$ = 125Hz, $^1J_{H,Si}$ = 202 Hz, 1H, SiH), 3.50 (s, 6H, N-CH_3), 1.89 (s, 18H, C(CH_3)$_3$). **^{13}C{^{1}H} NMR** (126 MHz, CD_2Cl_2, 298 K): δ 179.4 (Pt=C), 162.2 (q, $J_{C,B}$ = 50 Hz, C_{ipso}-BAr^F), 136.4 (Ph), 135.3 (br, C_{ortho}-BAr^F), 134.5 (Ph), 130.0 (Ph), 129.4 (br, q $J_{C,F}$ = 32 Hz, C_{meta}-BAr^F), 128.4 (Ph), 125.0 (q, $J_{C,F}$ = 272 Hz, CF_3-BAr^F), 121.9 and 120.7 (=CH), 117.9 (br, C_{para}-BAr^F), 59.9 (***C***(CH_3)$_3$), 39.3 (N-CH_3), 33.1 (C(***C***H_3)$_3$). Elem. Anal. Calcd. for $C_{60}H_{51}BF_{24}N_4PtSi$: C, 47.47; H, 3.39; N, 3.69. Found: C, 47.43; H, 3.56; N, 3.89.

Complex [Pt(SiH$_2$Ph)(I^{t}BuMe)$_2$][BAr^F], 6: ^{1}H NMR (500 MHz, CD_2Cl_2, 298 K): δ 7.74 (br, 8H, H_{ortho}-BAr^F), 7.58 (br, 4H, H_{para}-BAr^F), 7.32 (t, $^3J_{H,H}$ = 7.6 Hz, 1H, CH-Ph_{para}), 7.20–7.16 (m, 4H, =CH and CH-Ph_{meta}), 7.02 (d, $^3J_{H,H}$ = Hz, CH-Ph_{ortho}), 6.91 (s, 2H, =CH), 3.81 (s, 6H, N-CH_3), 3.71 (s+d, 2H, $^2J_{H,Pt}$ = 110.4 Hz, SiH_2), 1.90 (s, 18H, C(CH_3)$_3$). **^{13}C{^{1}H} NMR** (126 MHz, CD_2Cl_2, 298 K): δ 177.4 (Pt=C), 162.2 (q, $J_{C,B}$ = 50 Hz, C_{ipso}-BAr^F), 135.2 (br, C_{ortho}-BAr^F), 134.5 (Ph_{ortho}), 130.1 (Ph_{para}), 128.5 (Ph_{meta}), 129.3 (br, q $J_{C,F}$ = 32 Hz, C_{meta}-BAr^F), 125.0 (q, $J_{C,F}$ = 272 Hz, CF_3-BAr^F), 122.2 (s+d, $^3J_{Pt,C}$ = 33 Hz, =CH), 120.6 (s+d, $^3J_{Pt,C}$ = 38 Hz, =CH), 117.9 (br, C_{para}-BAr^F), 59.8 (***C***(CH_3)$_3$), 39.4 (N-CH_3), 32.6 (C(***C***H_3)$_3$). Elem. Anal. Calcd. for $C_{54}H_{47}BF_{24}N_4PtSi$: C, 44.98; H, 3.29; N, 3.89. Found: C, 44.79; H, 3.49; N, 3.90.

13. NMR Spectra

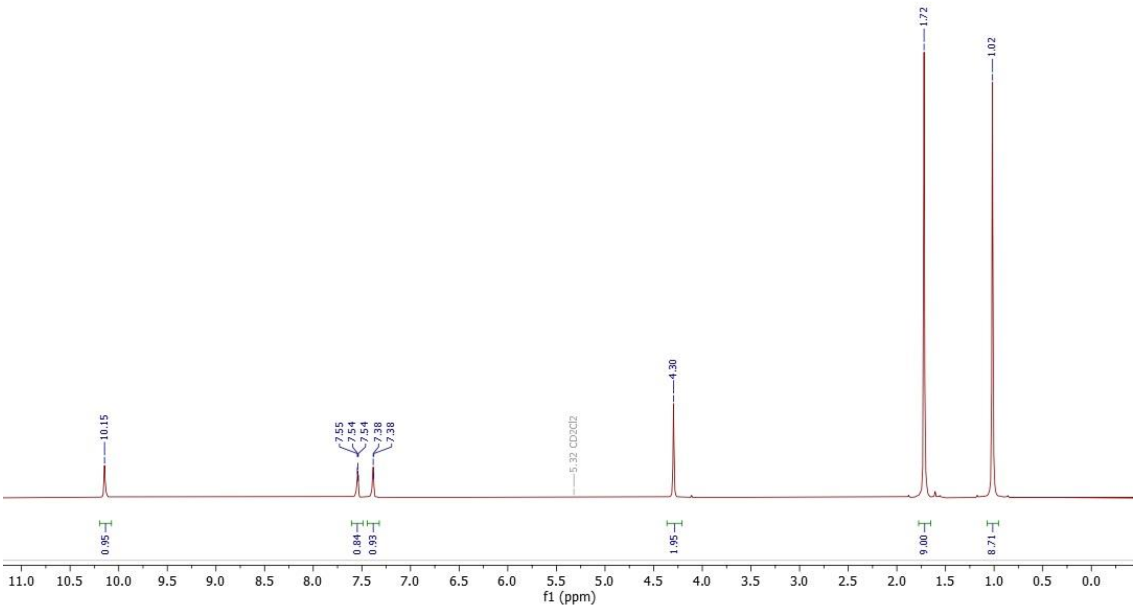

Figure S1. ^{1}H NMR (400 MHz) of I^{t}Bu^{n}Pe·HI in CD_2Cl_2 at 298 K.

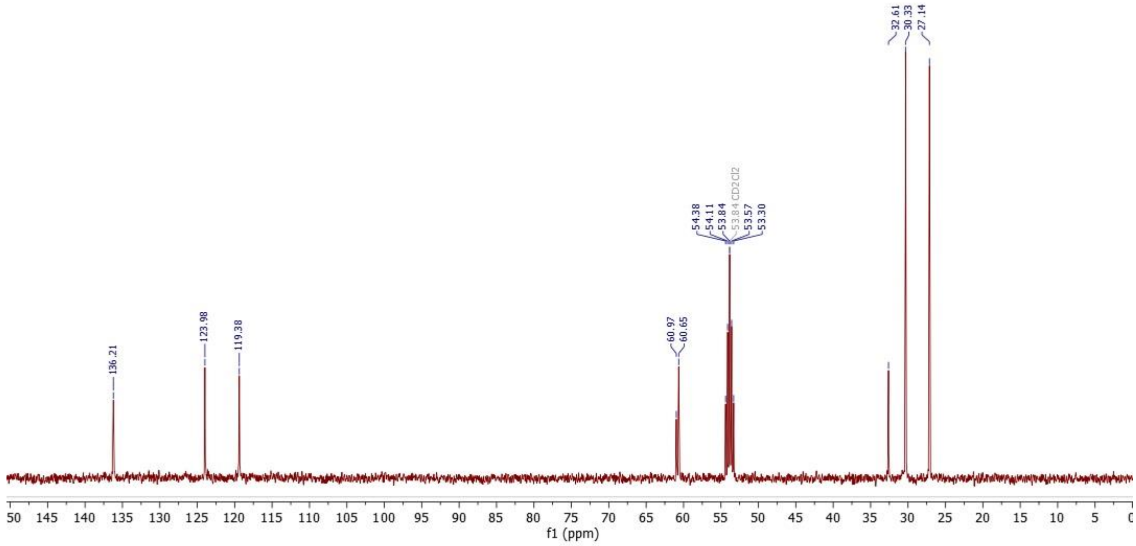

Figure S2.^{13}C{^{1}H} NMR of I^{t}Bu^{n}Pe·HI in CD_2Cl_2 at 298 K.

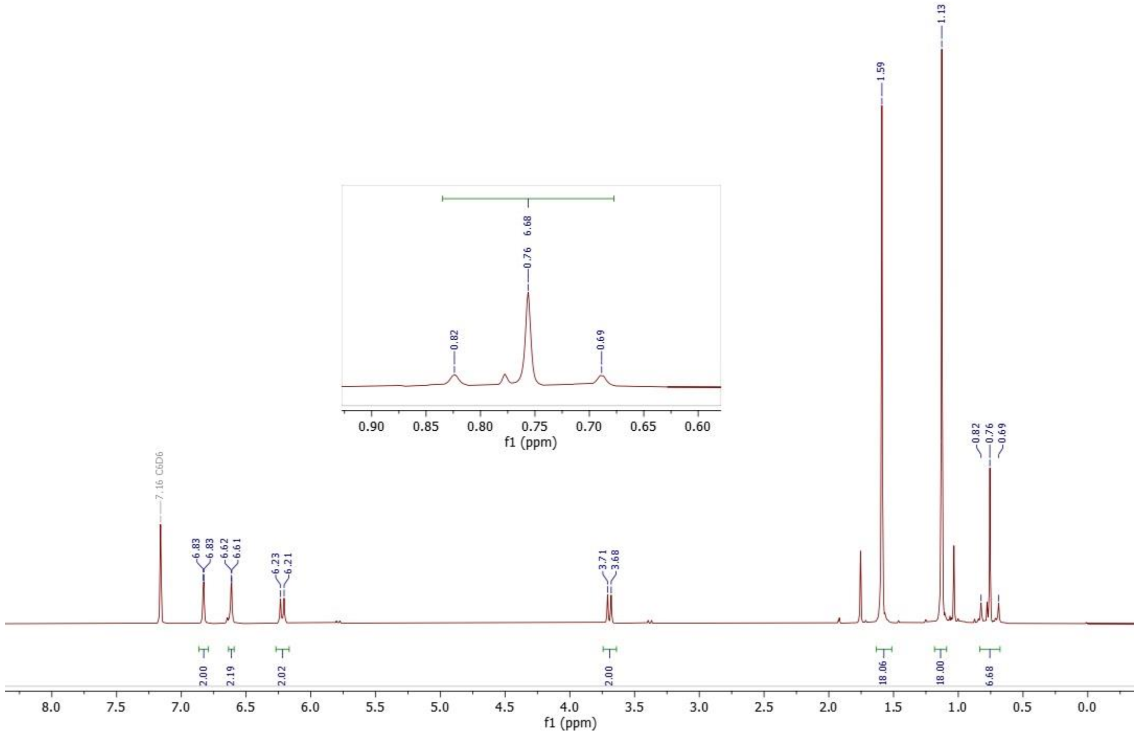

Figure S3. ^{1}H NMR (400 MHz) of $[Pt(CH_3)_2(I^tBu^nPe)]$ in CD_2Cl_2 at 298 K.

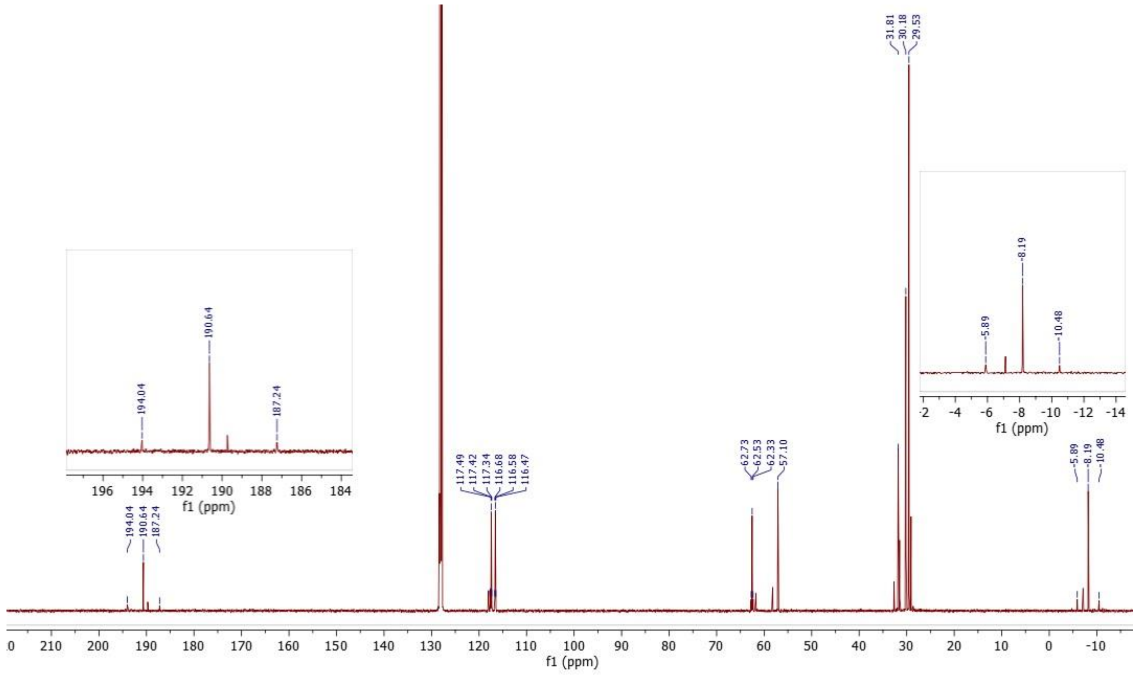

Figure S4. $^{13}C\{^{1}H\}$ NMR of $[Pt(CH_3)_2(I^tBu^nPe)]$ in CD_2Cl_2 at 298 K.

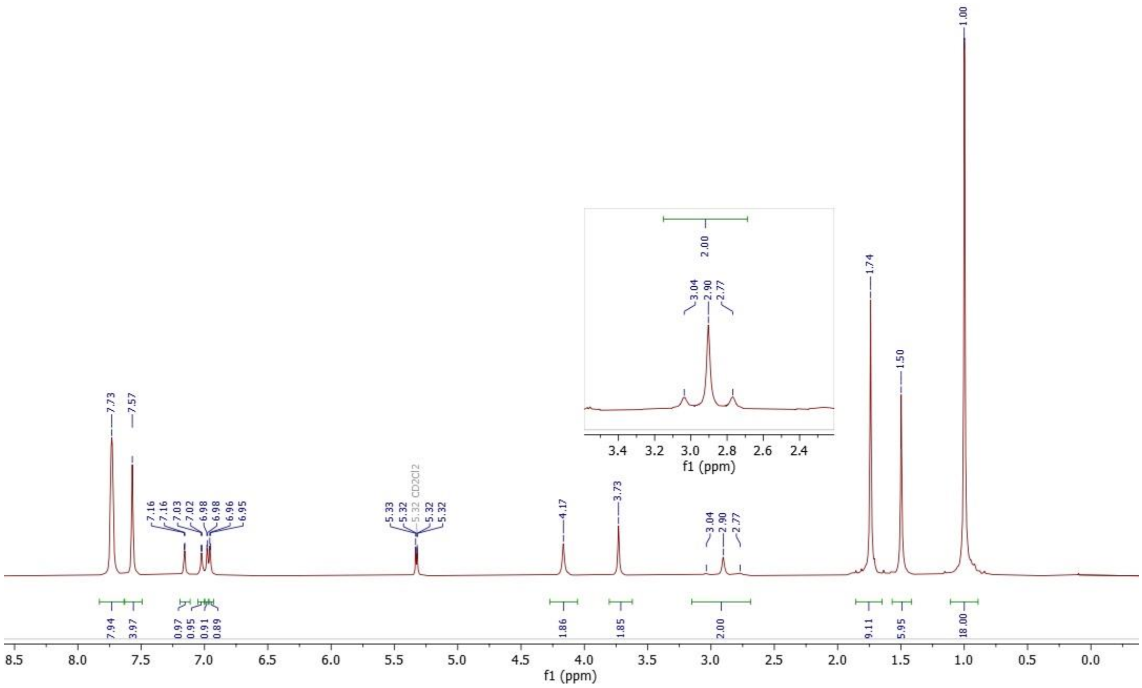

Figure S5. ^{1}H NMR (400 MHz) of **1.3** in CD_2Cl_2 at 298 K.

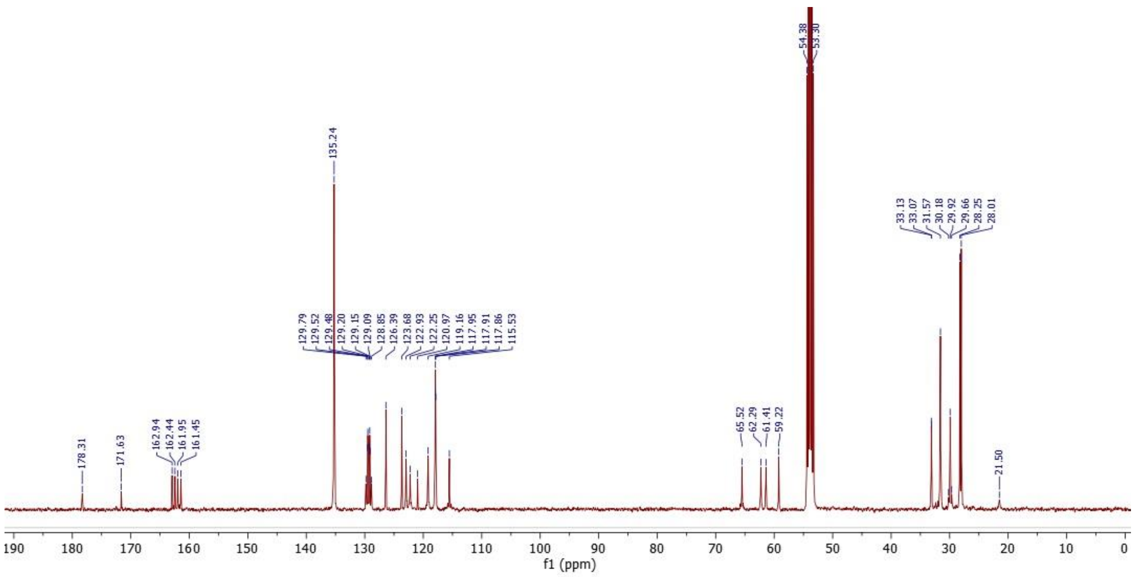

Figure S6. $^{13}C\{^{1}H\}$ NMR of **1.3** in CD_2Cl_2 at 298 K.

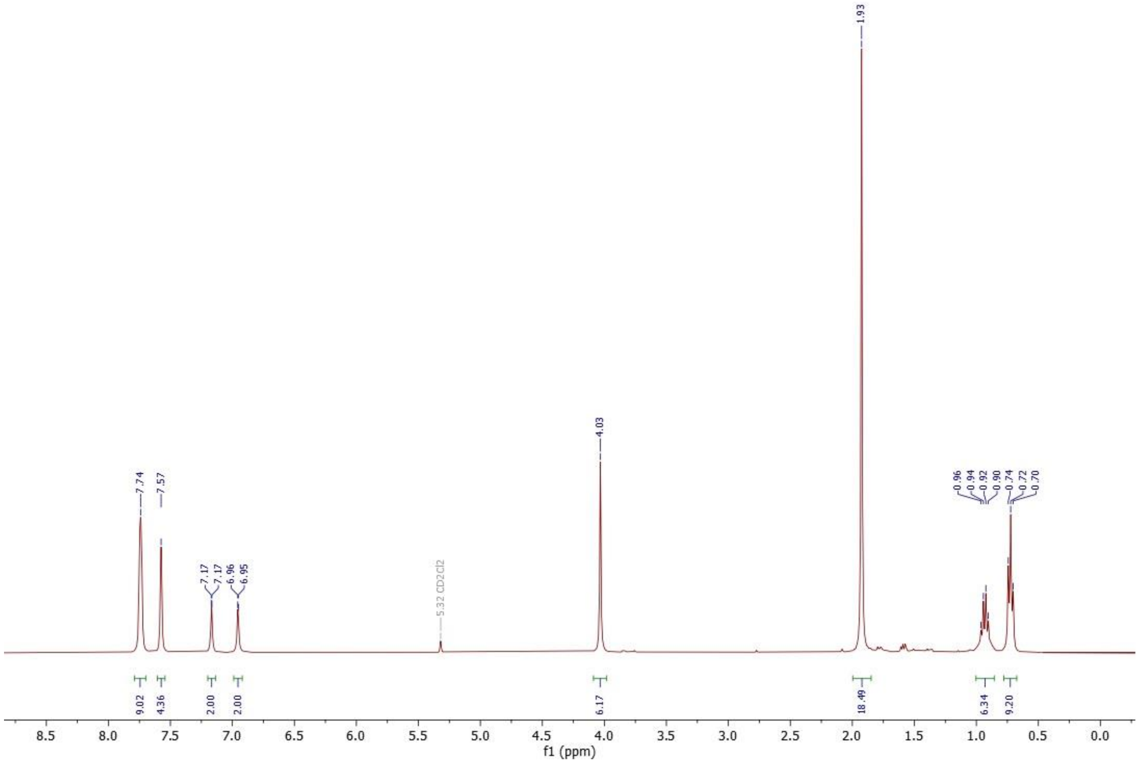

Figure S7. ^{1}H NMR (400 MHz) of complex **2.1a** in CD_2Cl_2 at 298 K.

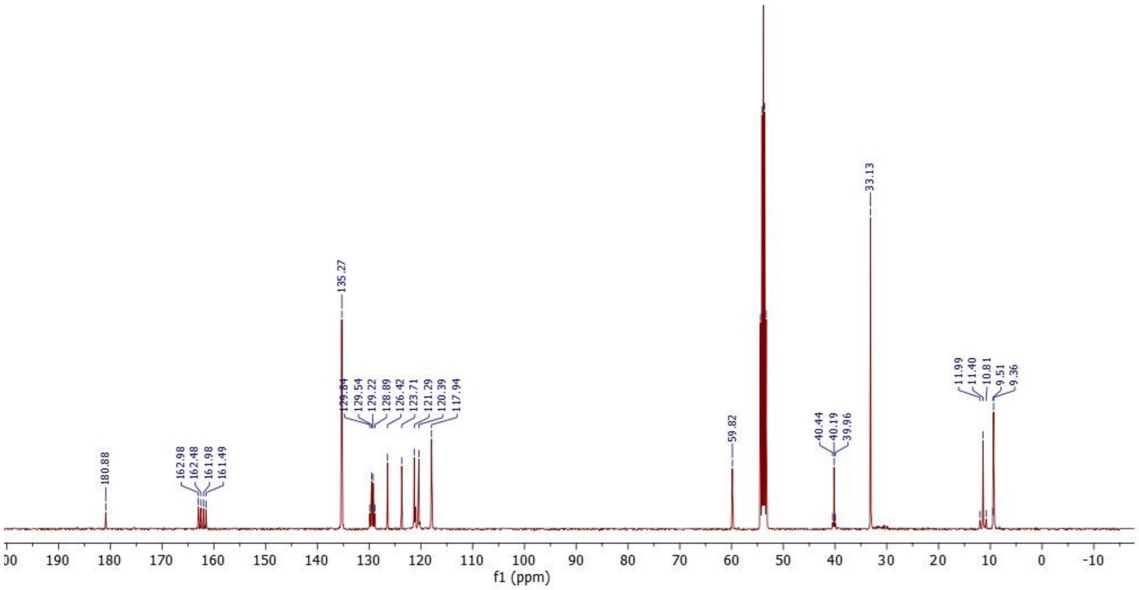

Figure S8. $^{13}C\{^{1}H\}$ NMR (100 MHz) of complex **2.1a** in CD_2Cl_2 at 298 K.

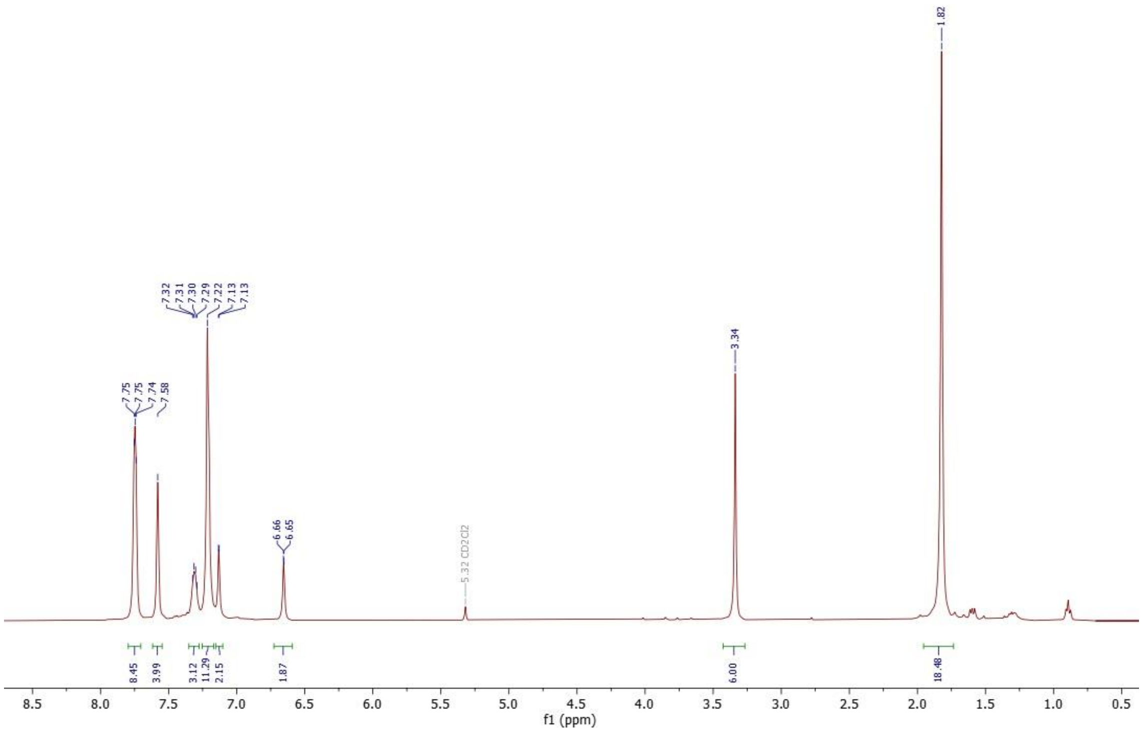

Figure S9. ^{1}H NMR (300 MHz) of complex **2.1b** in CD_2Cl_2 at 298 K.

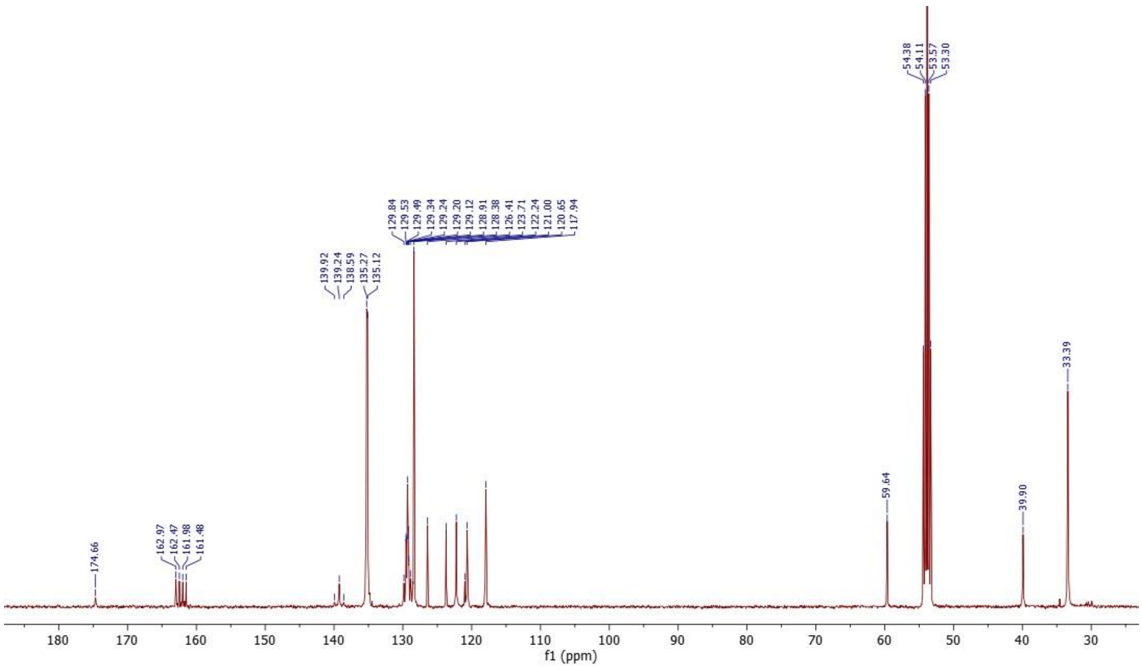

Figure S10. $^{13}C\{^{1}H\}$ NMR (100 MHz) of complex **2.1b** in CD_2Cl_2 at 298 K.

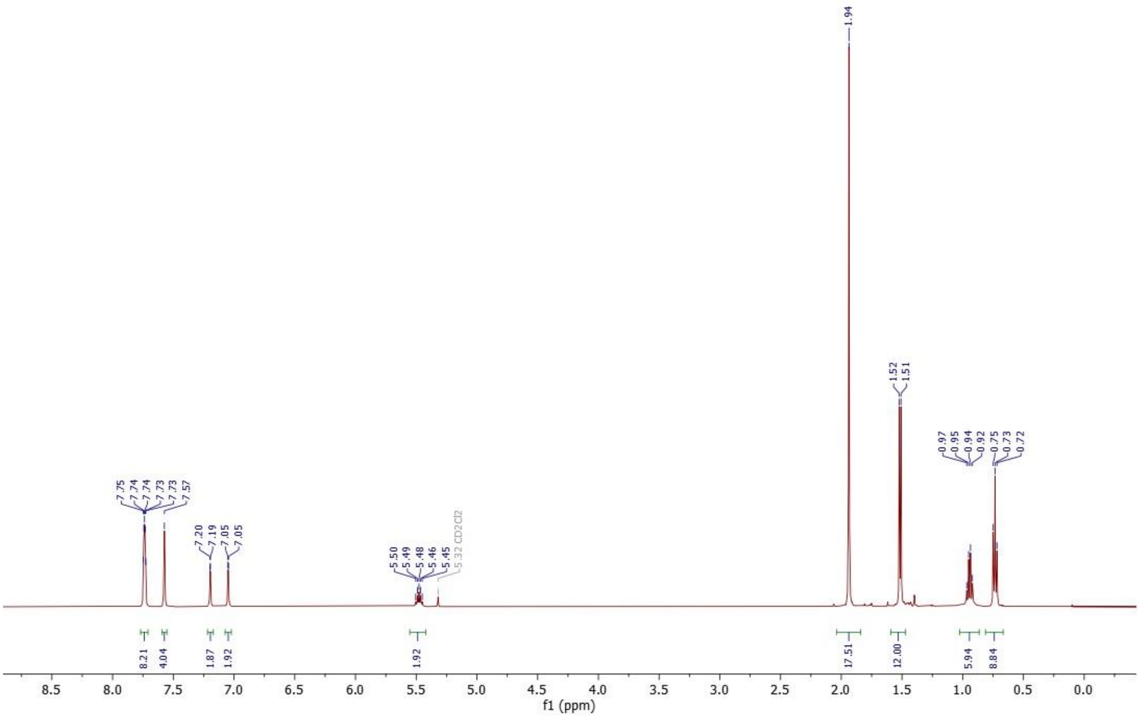

Figure S11. ^{1}H NMR (500 MHz) of complex **2.2a** in CD_2Cl_2 at 298 K.

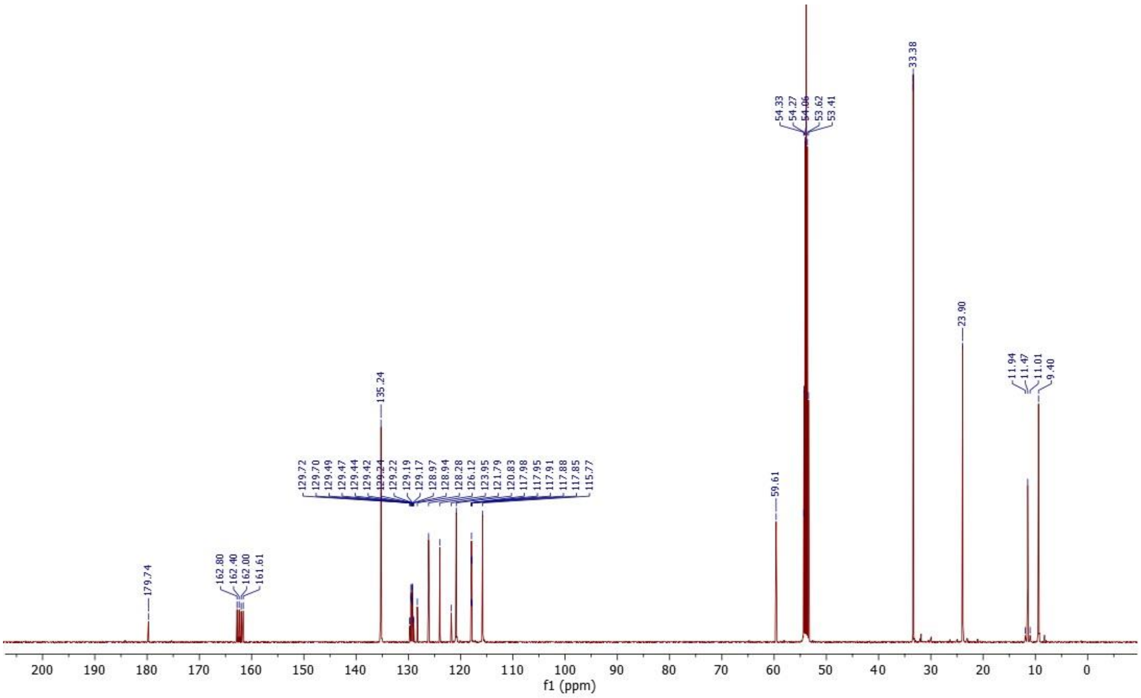

Figure S12. $^{13}C\{^{1}H\}$ NMR (126 MHz) of complex **2.2a** in CD_2Cl_2 at 298 K.

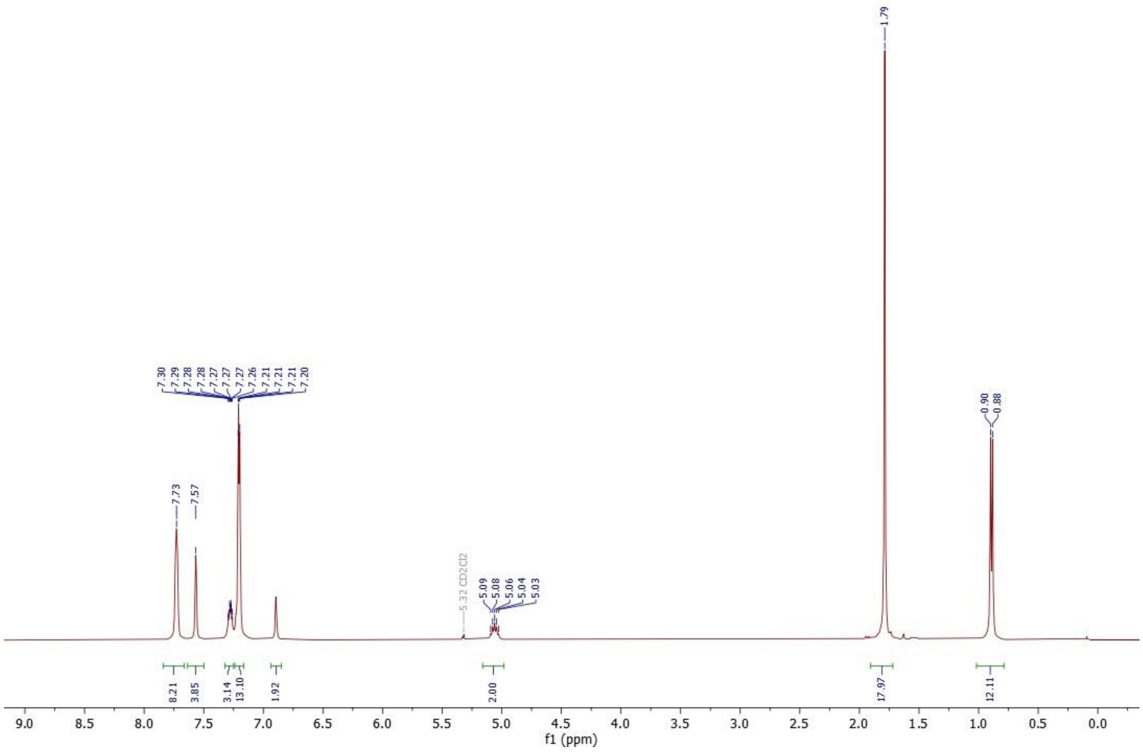

Figure S13. ^{1}H NMR (400 MHz) of complex **2.2b** in CD_2Cl_2 at 298 K.

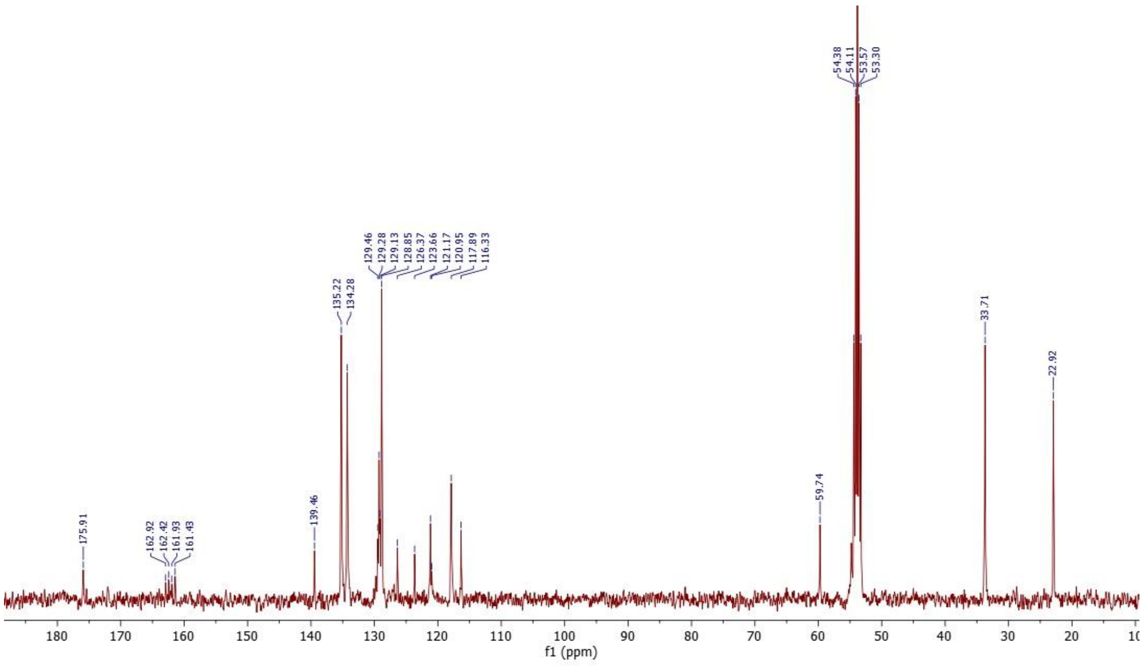

Figure S14. $^{13}C\{^{1}H\}$ NMR (100 MHz) of complex **2.2b** in CD_2Cl_2 at 298 K.

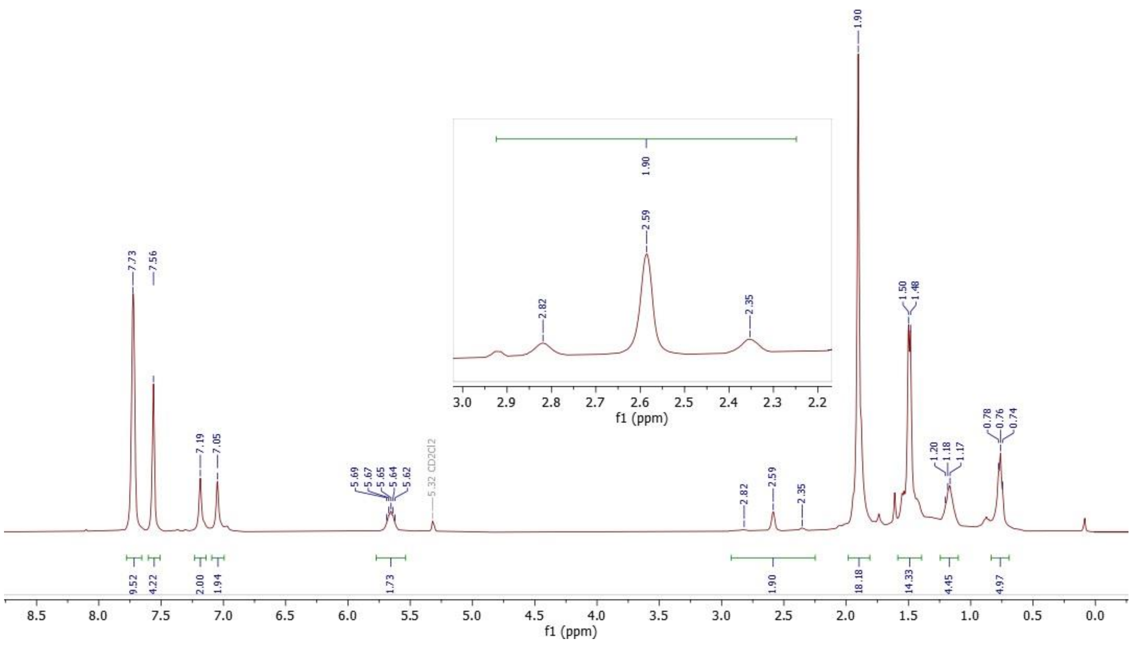

Figure S15. ^{1}H NMR (400 MHz) of complex **2.2c** in CD_2Cl_2 at 298 K.

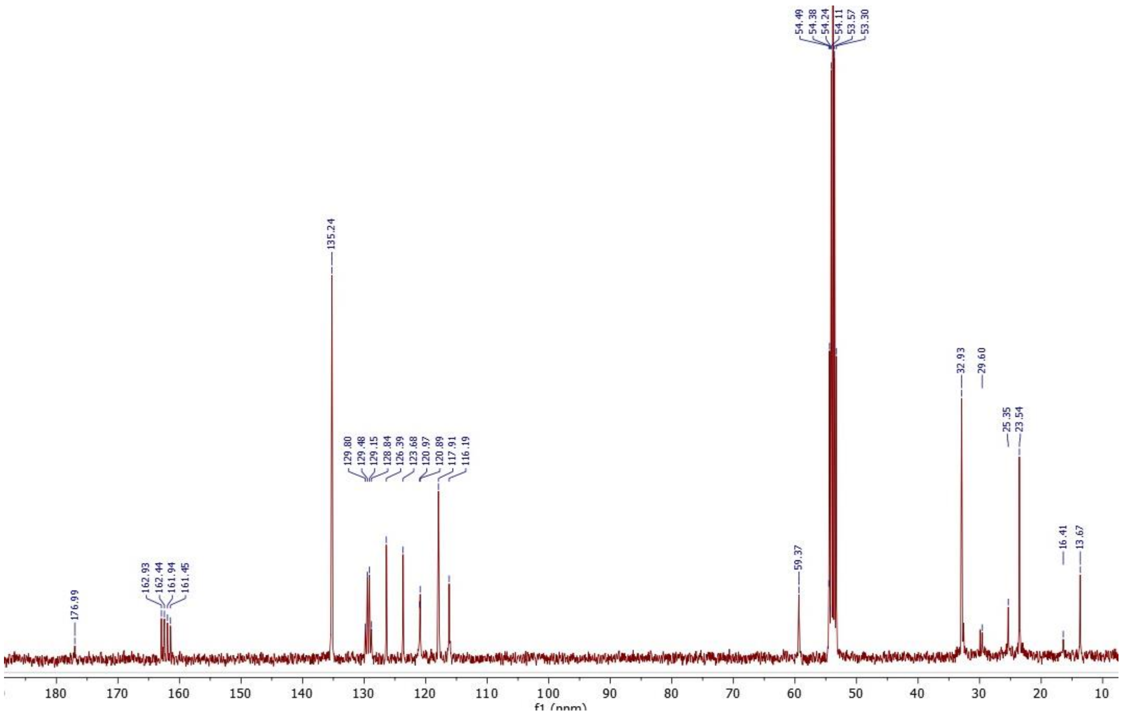

Figure S16. $^{13}C\{^{1}H\}$ NMR (100 MHz) of complex **2.2c** in CD_2Cl_2 at 298 K.

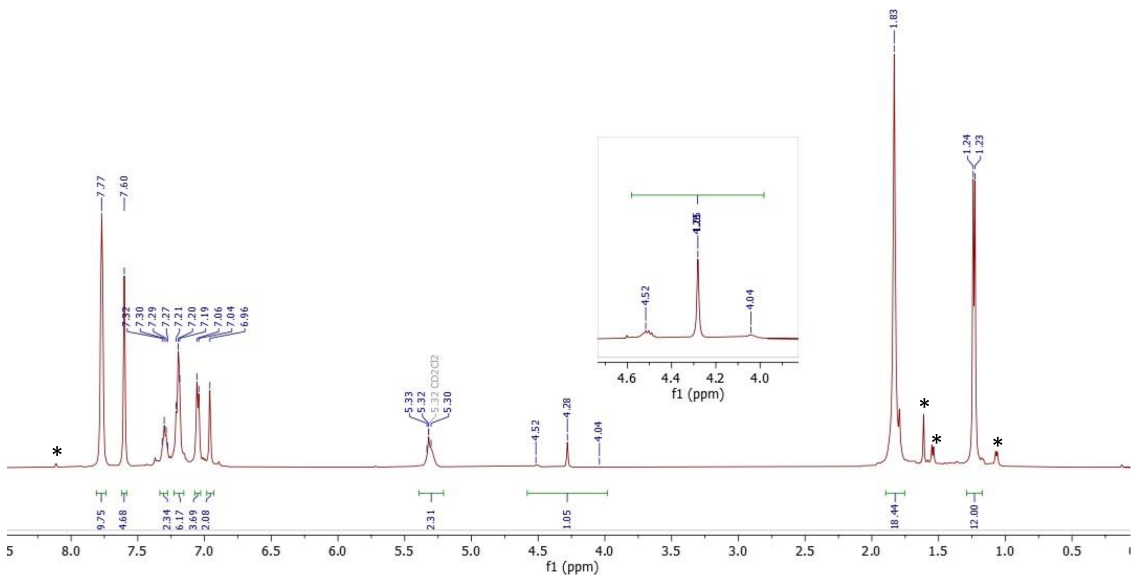

Figure S17. ^{1}H NMR (500 MHz) of complex **2.2d** in CD_2Cl_2 at 298 K. The sample contains a small amount of imidazolium salt (~5 %).

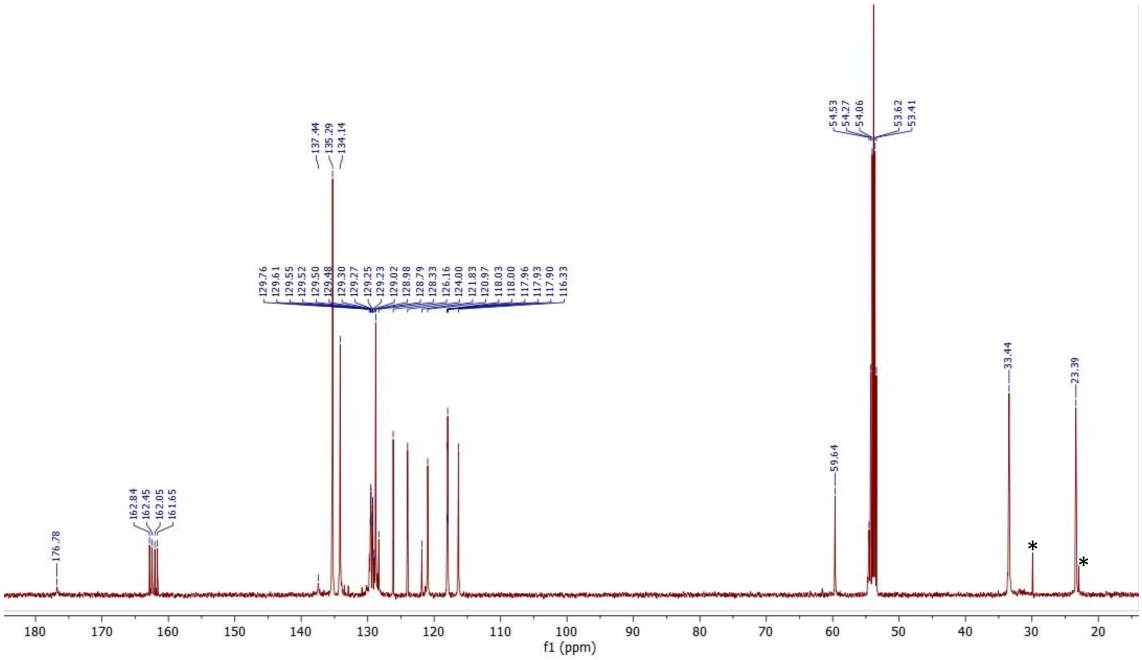

Figure S18. $^{13}C\{^{1}H\}$ NMR (126 MHz) of complex **2.2d** in CD_2Cl_2 at 298 K. The sample contains a small amount of imidazolium salt (~5 %).

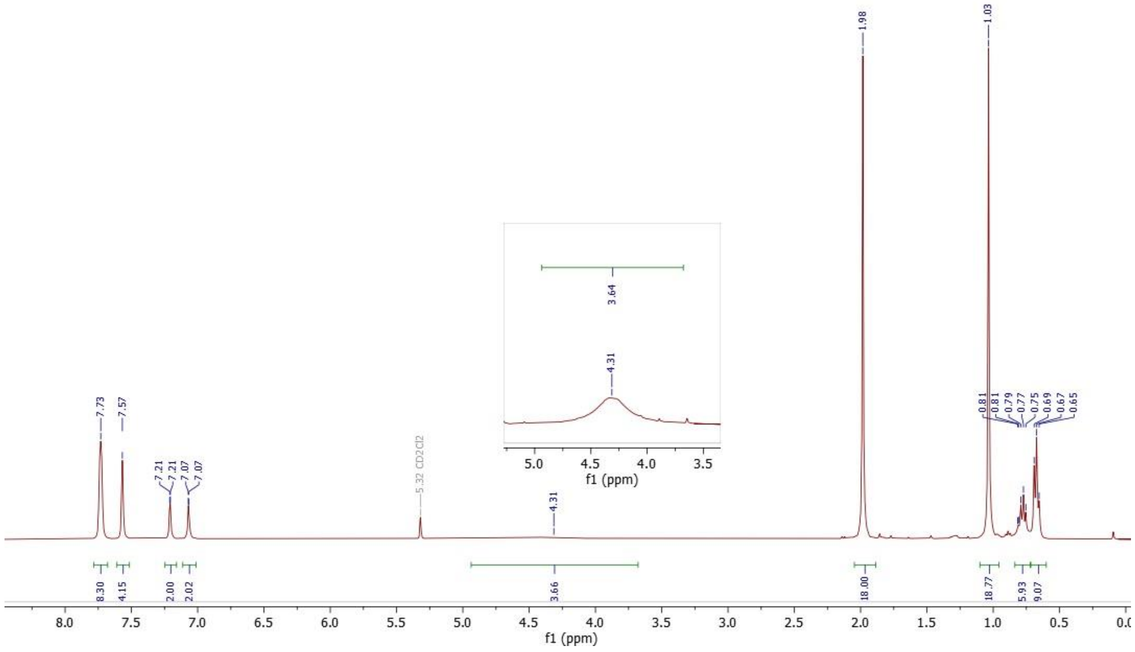

Figure S19. ^{1}H NMR (400 MHz) of **2.3a** in CD_2Cl_2 at 298 K.

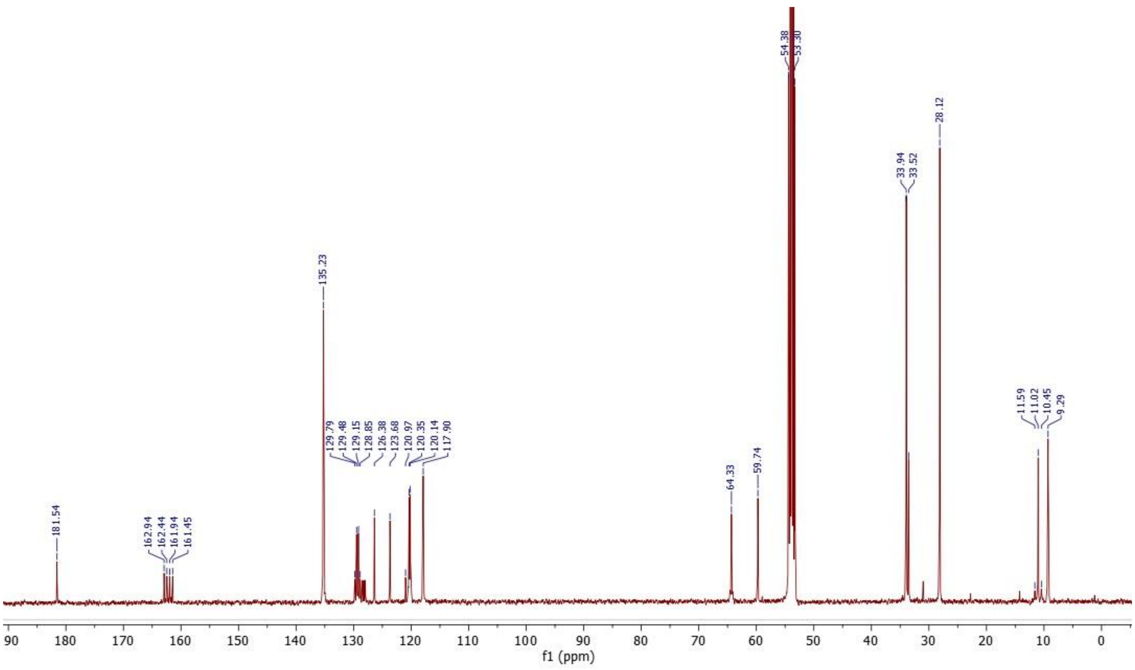

Figure S20. $^{13}C\{^1H\}$ NMR of **2.3a** in CD_2Cl_2 at 298 K.

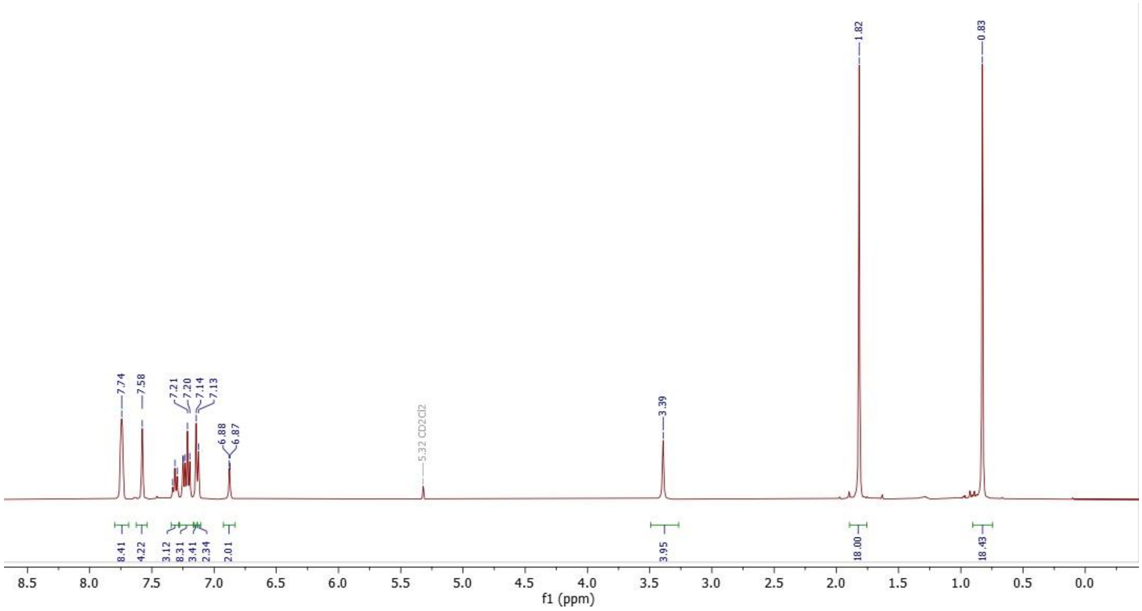

Figure S21. ^{1}H NMR (400 MHz) of **2.3b** in CD_2Cl_2 at 298 K.

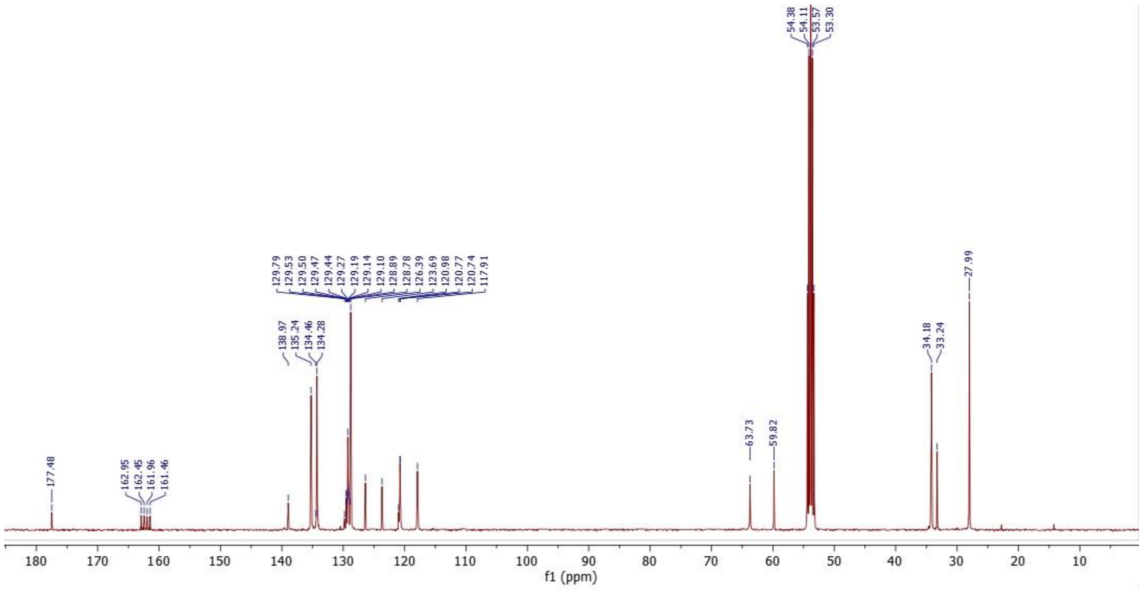

Figure S22. $^{13}C\{^{1}H\}$ NMR of **2.3b** in CD_2Cl_2 at 298 K.

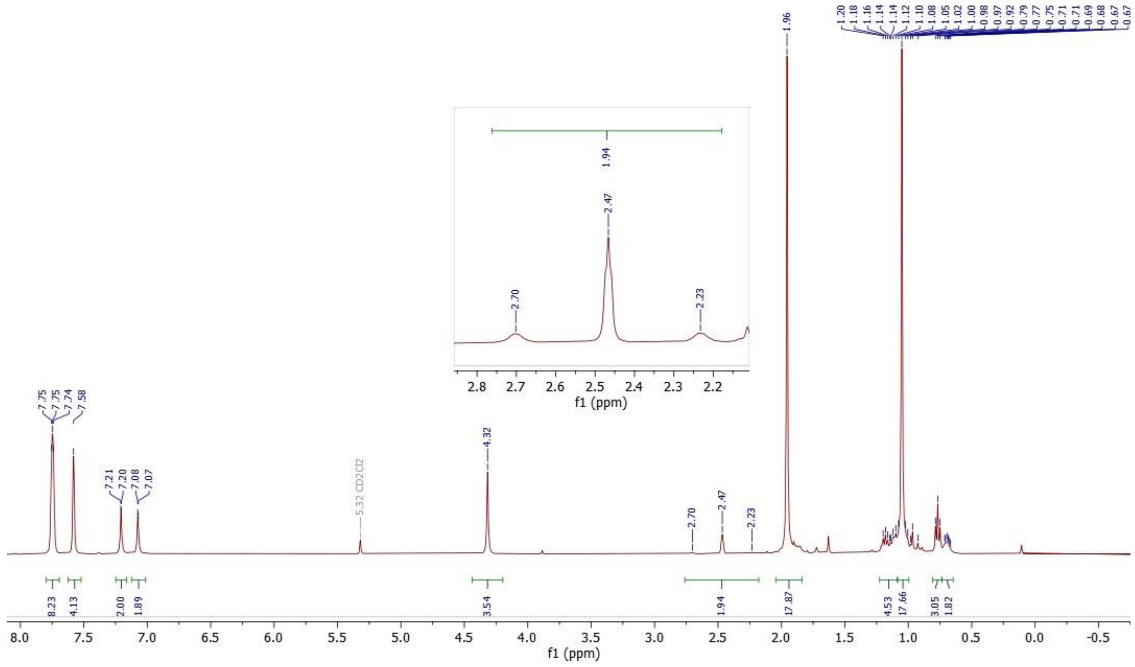

Figure S23. ^{1}H NMR (400 MHz) of complex **2.3c** in CD_2Cl_2 at 298 K.

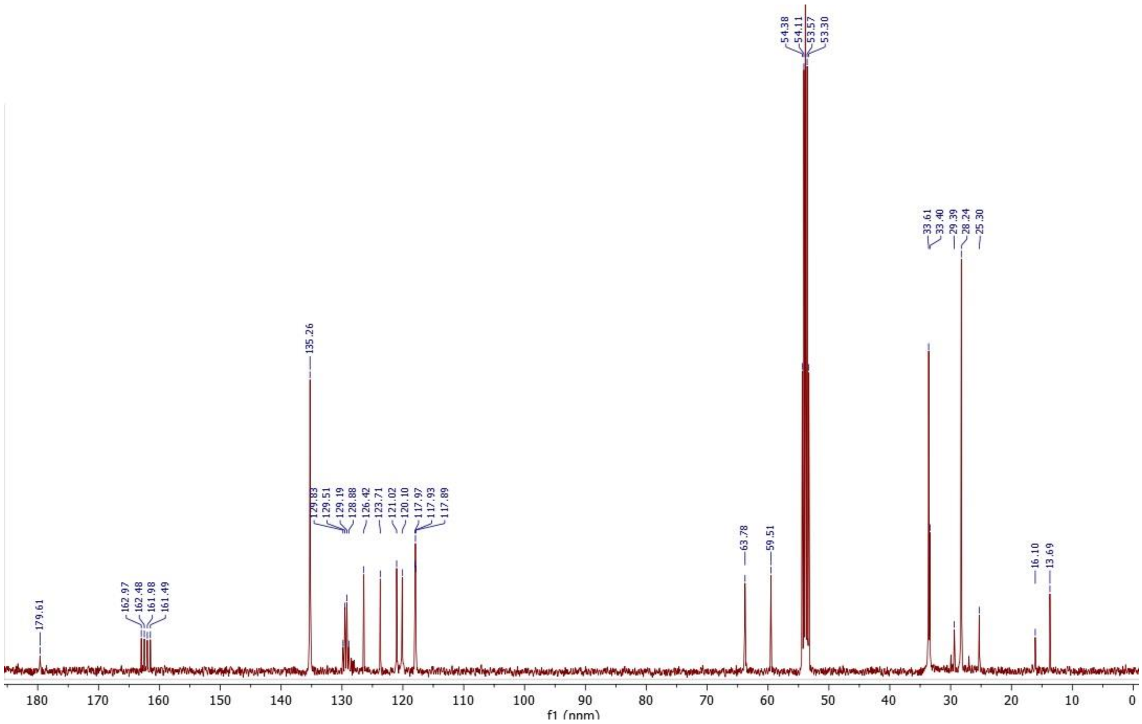

Figure S24. $^{13}C\{^{1}H\}$ NMR (100 MHz) of complex **2.3c** in CD_2Cl_2 at 298 K.

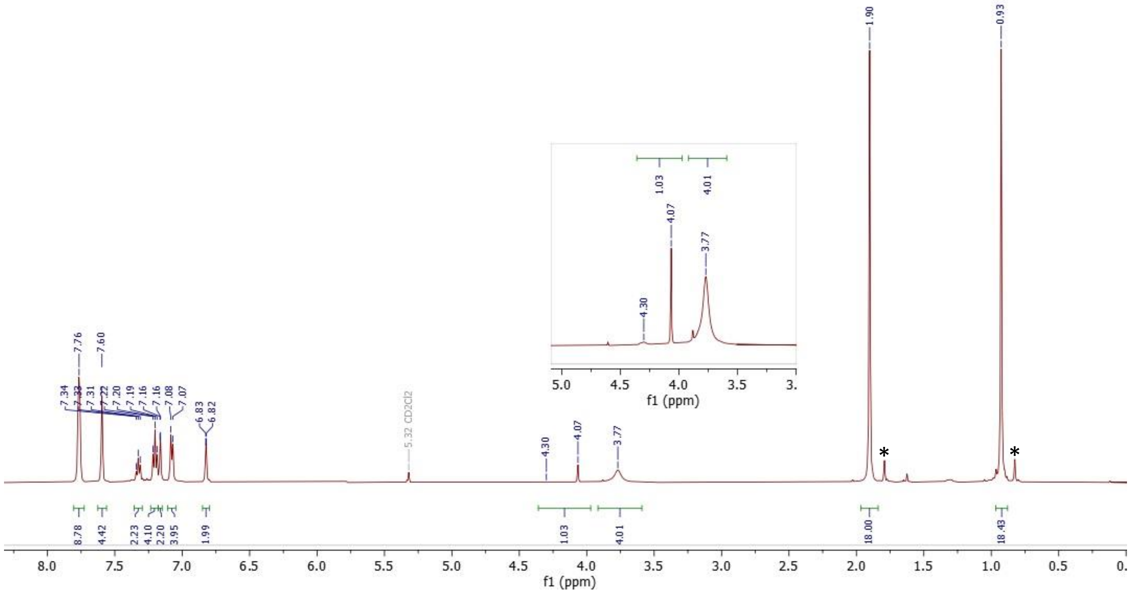

Figure S25. ^{1}H NMR (500 MHz) of complex **2.3d** in CD_2Cl_2 at 298 K. The sample contains a small amount of imidazolium salt (~2%).

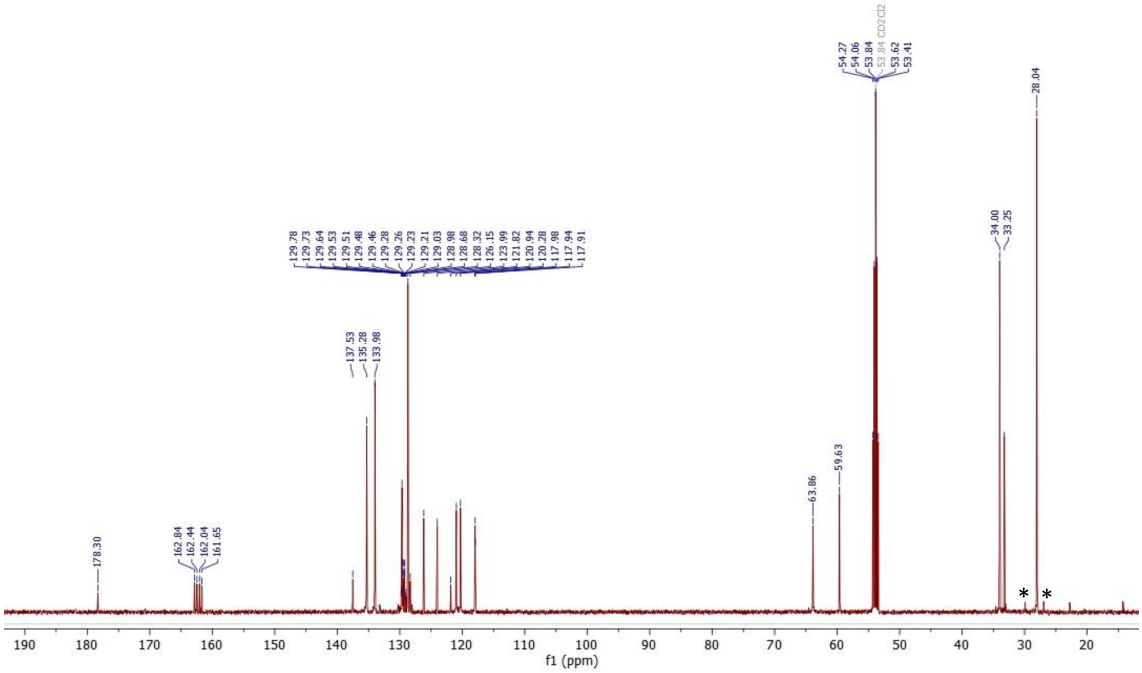

Figure S26. $^{13}C\{^{1}H\}$ NMR (126 MHz) of complex **2.3d** in CD_2Cl_2 at 298 K. The sample contains a small amount of imidazolium salt (~2%)

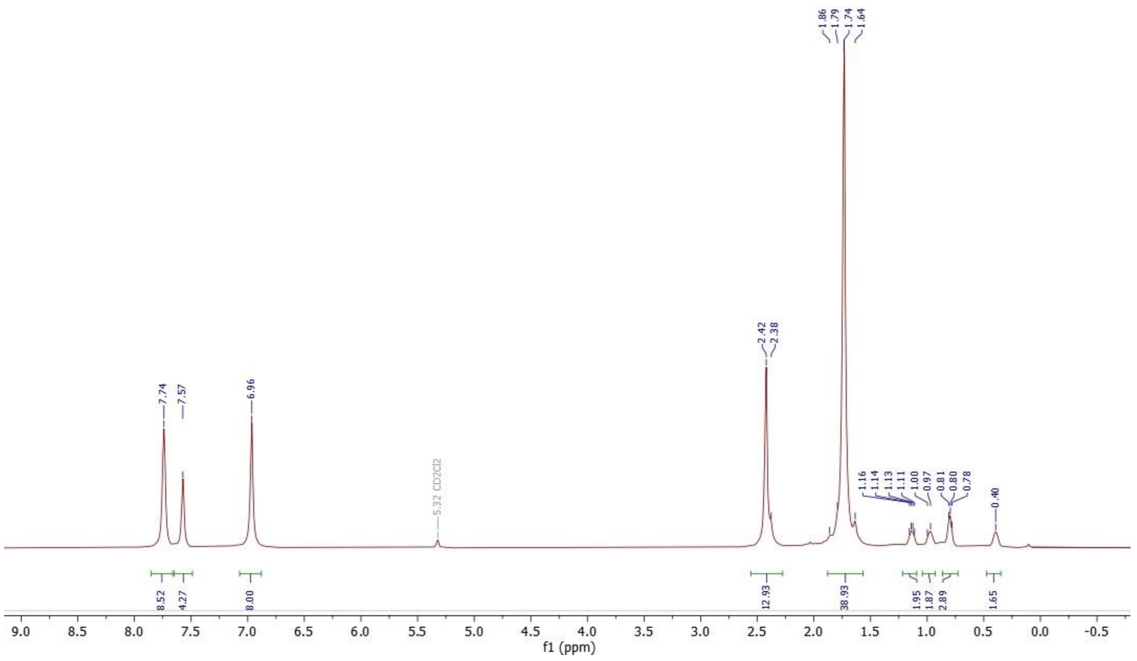

Figure S27. ^{1}H NMR (500 MHz) of complex **2.4c** in CD_2Cl_2 at 298 K.

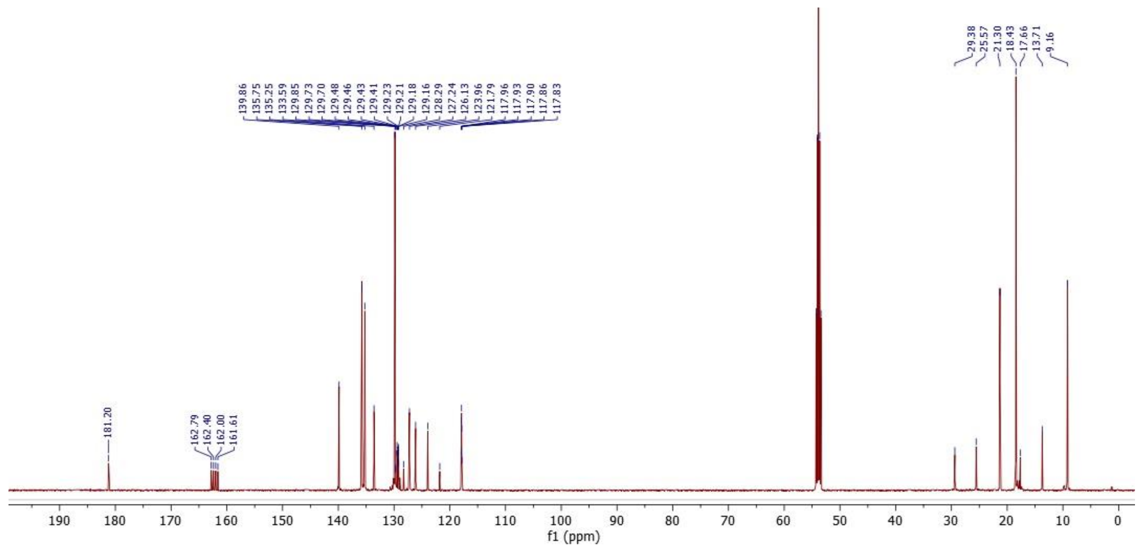

Figure S28. $^{13}C\{^{1}H\}$ NMR (126 MHz) of complex **2.4c** in CD_2Cl_2 at 298 K.

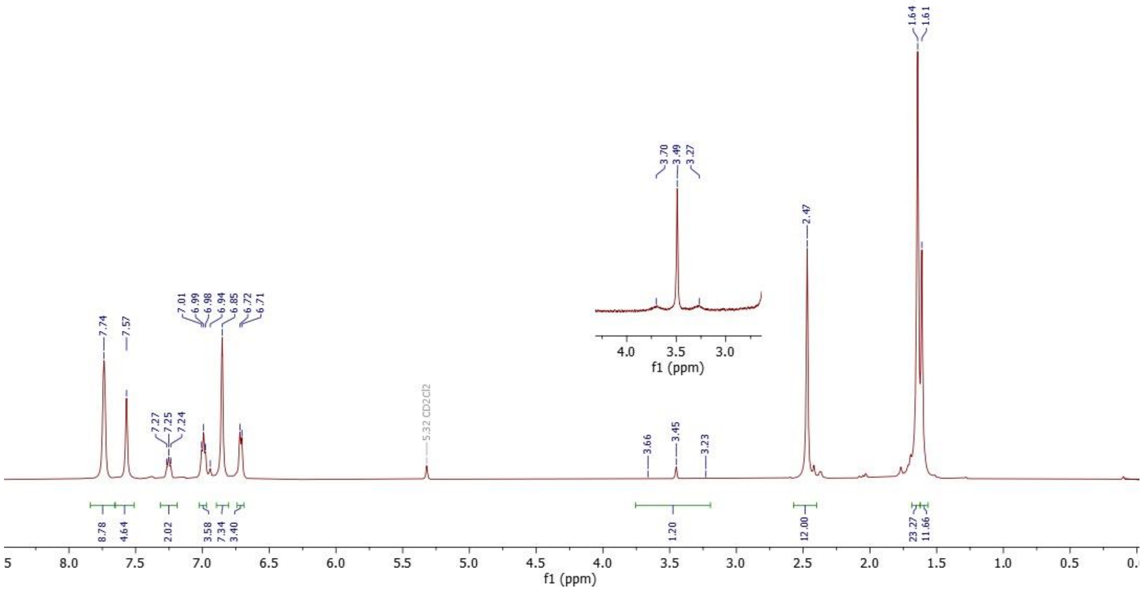

Figure S29. ^{1}H NMR (500 MHz) of complex **2.4d** in CD_2Cl_2 at 298 K.

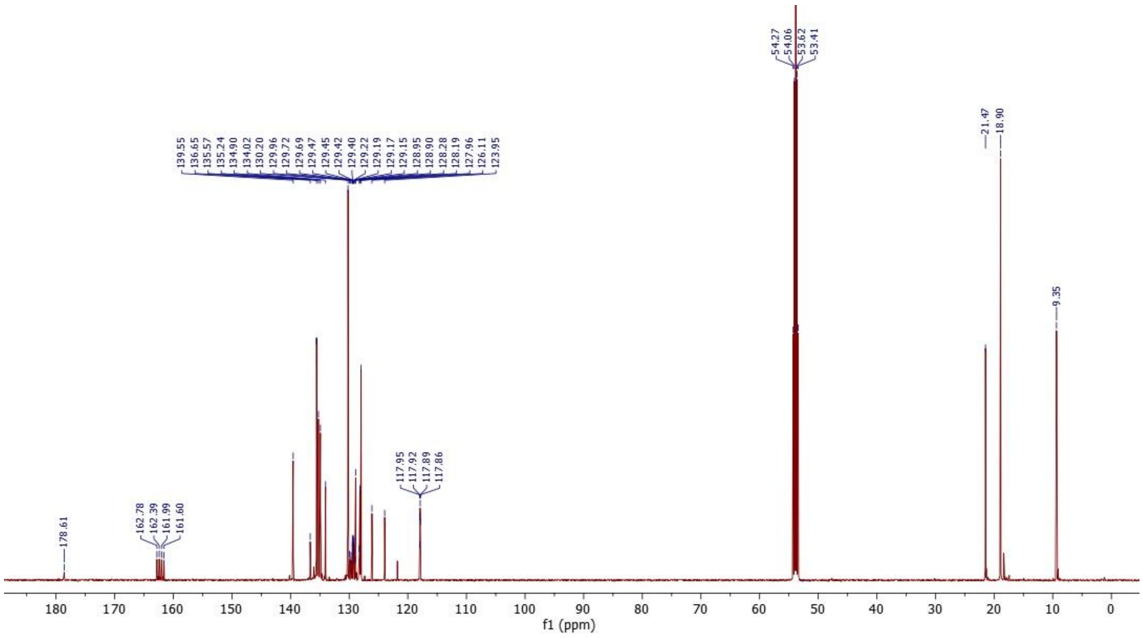

Figure S30. $^{13}C\{^{1}H\}$ NMR (126 MHz) of complex **2.4d** in CD_2Cl_2 at 298 K.

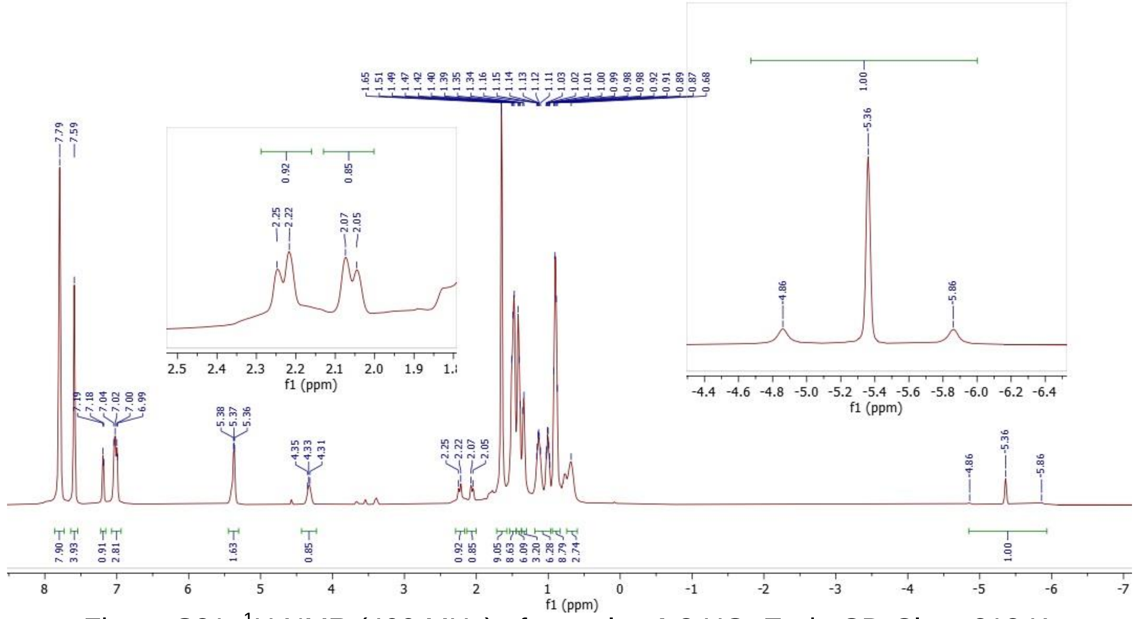

Figure S31. ^{1}H NMR (400 MHz) of complex **1.2**·$HGeEt_3$ in CD_2Cl_2 at 213 K.

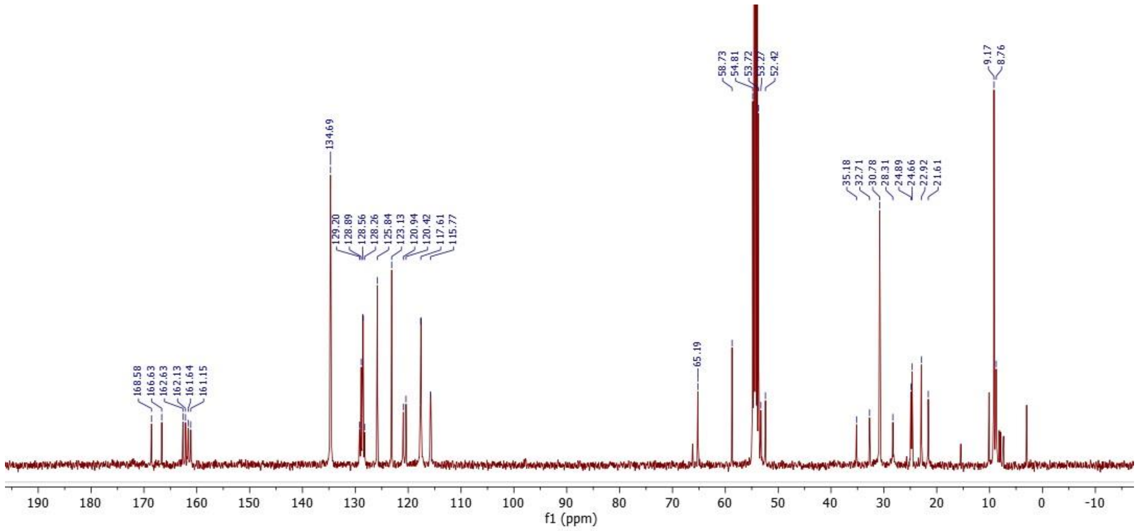

Figure S32. $^{13}C\{^{1}H\}$ NMR (100 MHz) of complex **1.2**·$HGeEt_3$ in CD_2Cl_2 at 213 K.

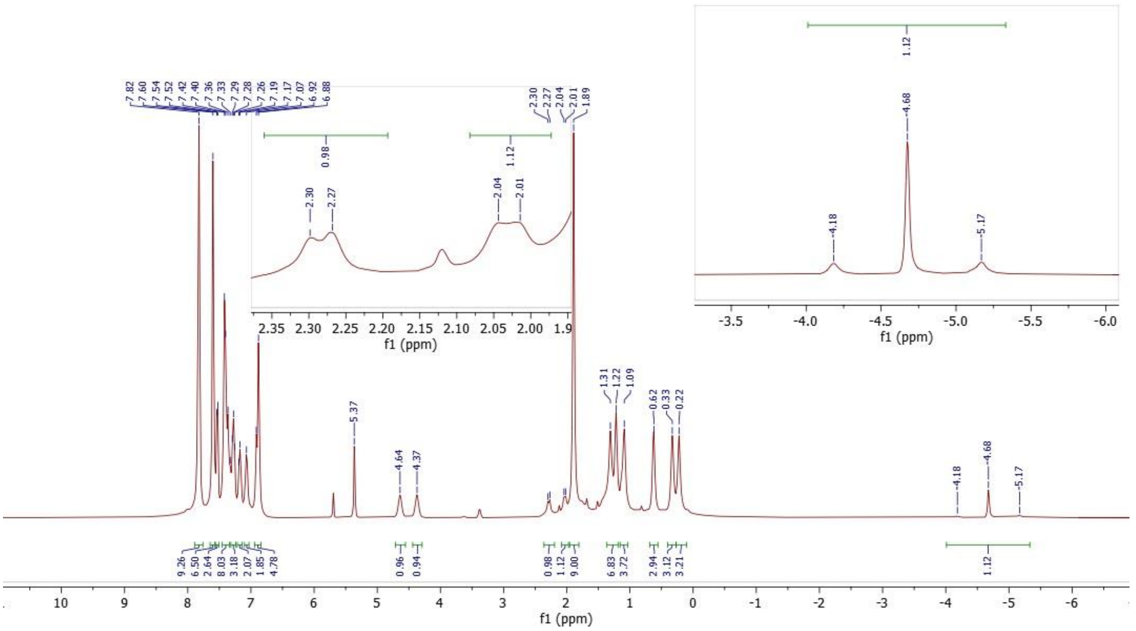

Figure S33. ^{1}H NMR (400 MHz) of complex **1.2**·$HGePh_3$ in CD_2Cl_2 at 198 K.

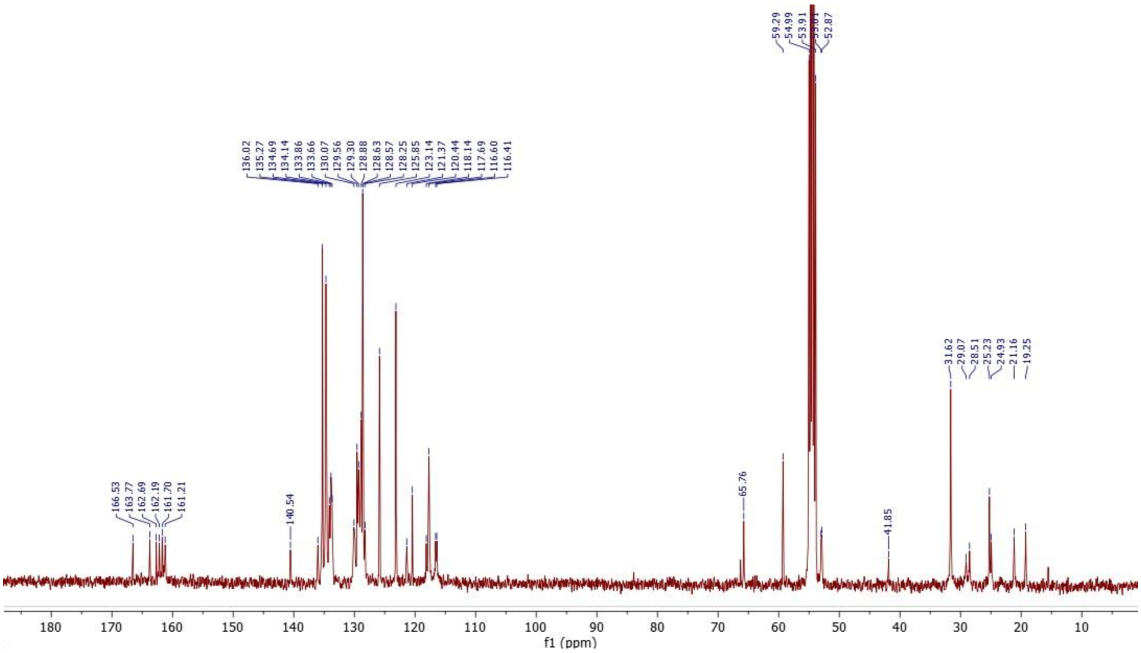

Figure S34. $^{13}C\{^{1}H\}$ NMR (100 MHz) of complex **1.2**·$HGePh_3$ in CD_2Cl_2 at 198 K.

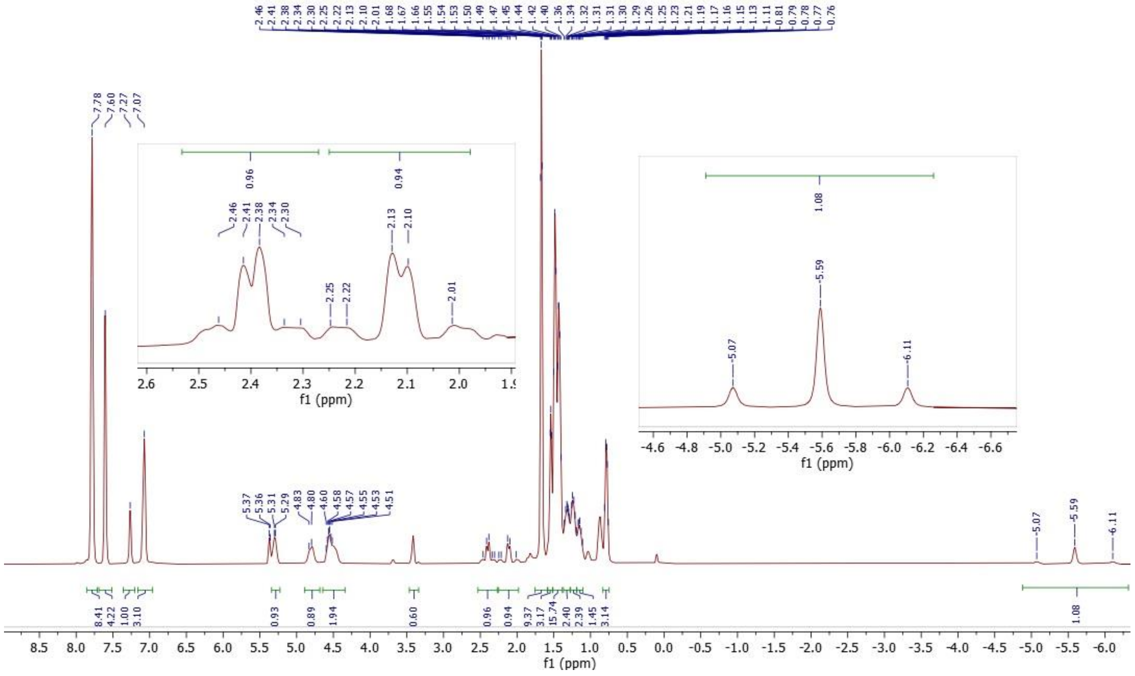

Figure S35. ^{1}H NMR (400 MHz) of complex **1.2**·$HGeH_2{}^{n}Bu$ in CD_2Cl_2 at 233 K.

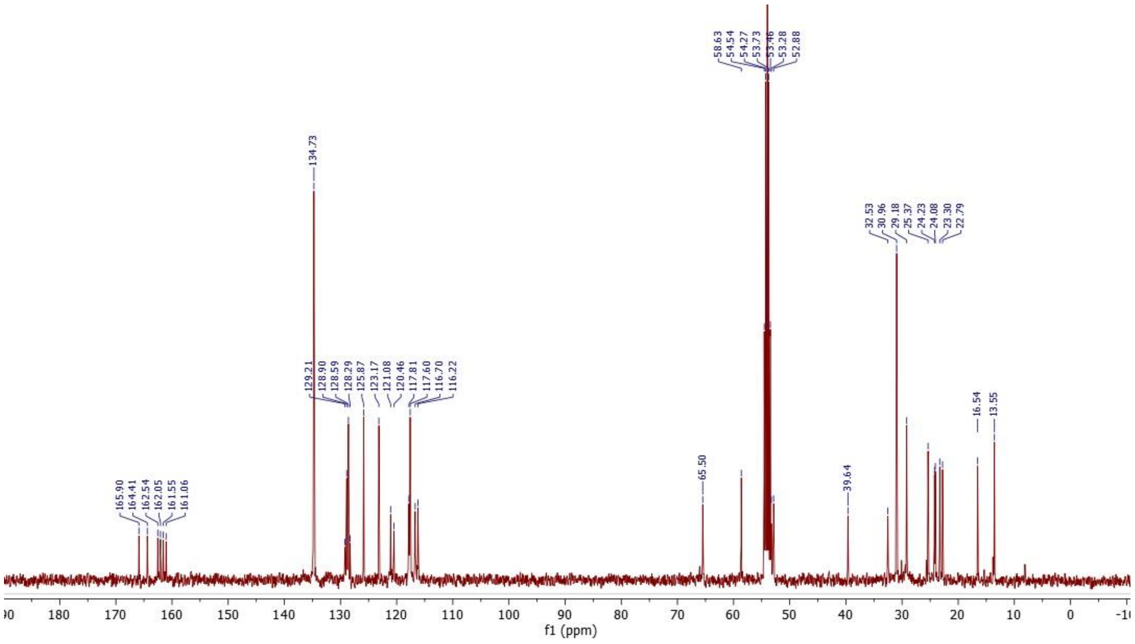

Figure S36. $^{13}C\{^{1}H\}$ NMR (100 MHz) of complex **1.2**·$HGeH_2{}^{n}Bu$ in CD_2Cl_2 at 233 K.

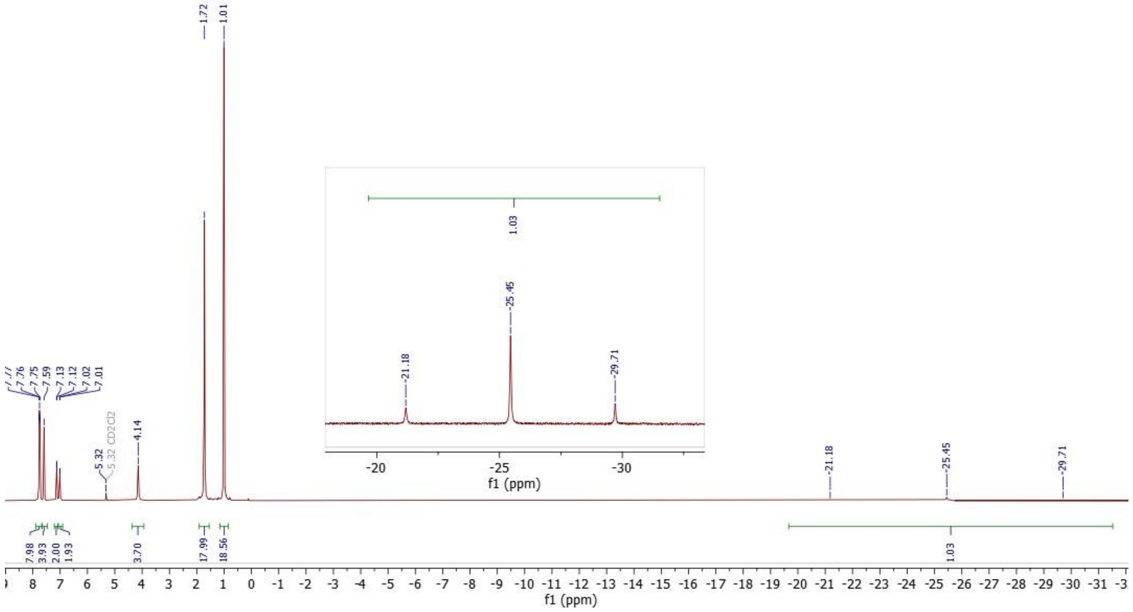

Figure S37. ^{1}H NMR (300 MHz) of complex **4** in CD_2Cl_2 at 298 K.

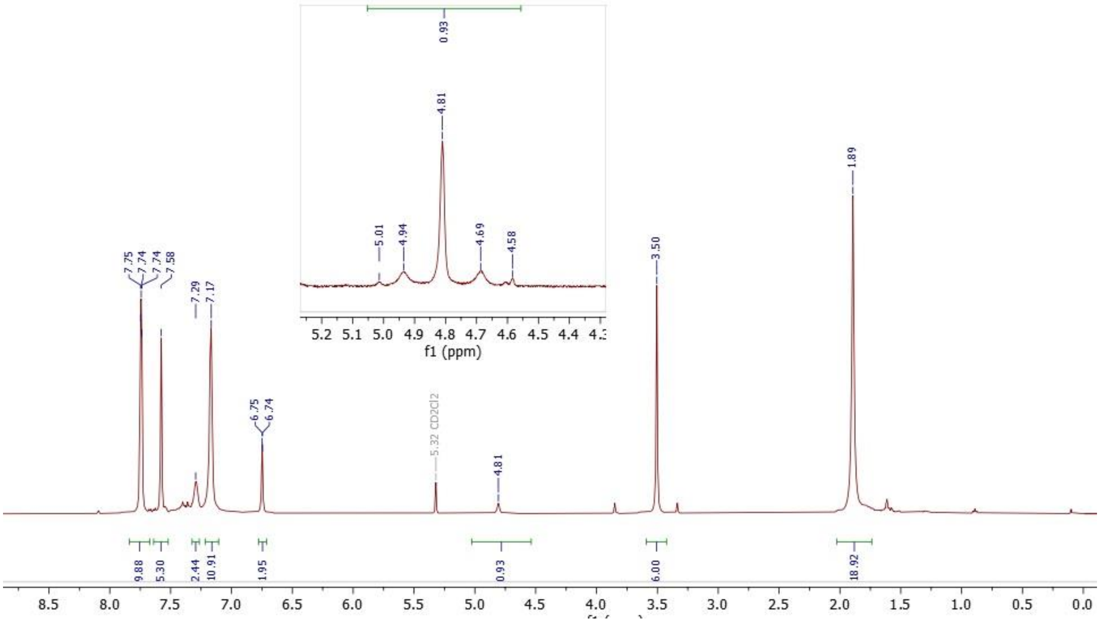

Figure S38. ^{1}H NMR (500 MHz) of complex **5** in CD_2Cl_2 at 298 K.

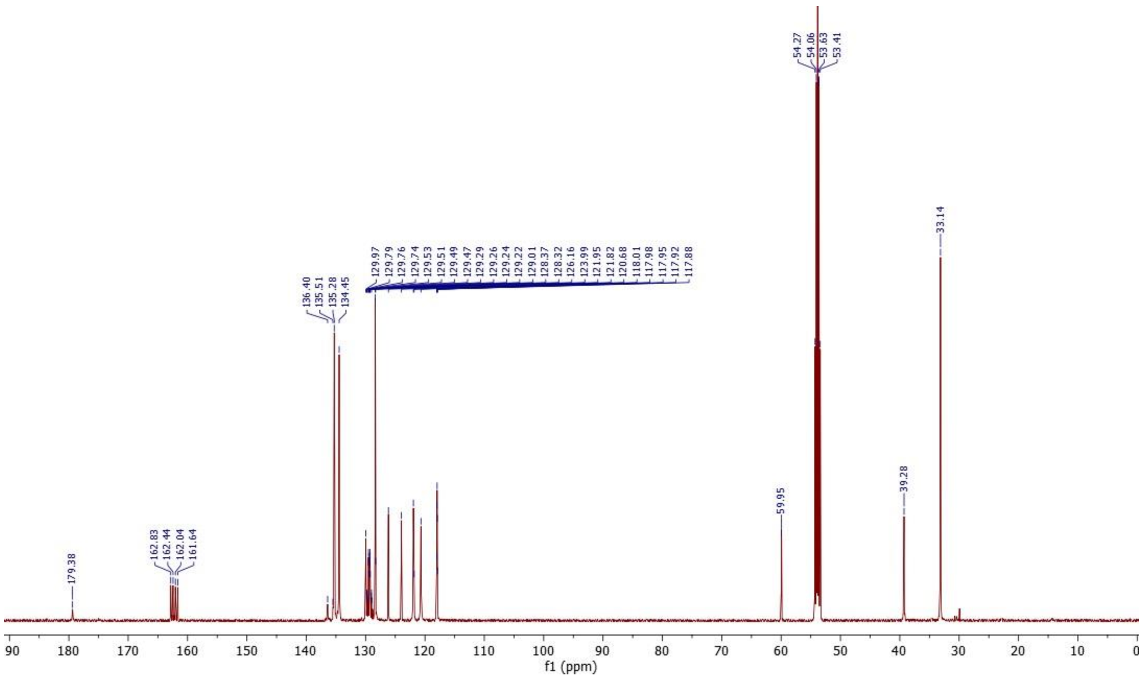

Figure S39. $^{13}C\{^{1}H\}$ NMR (126 MHz) of complex **5** in CD_2Cl_2 at 298 K.

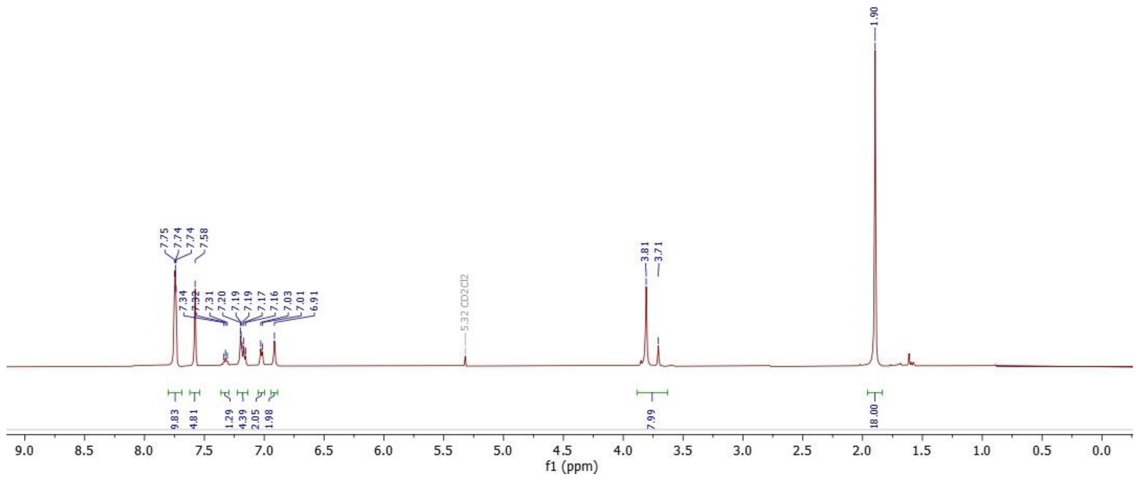

Figure S40. ^{1}H NMR (500 MHz) of complex **6** in CD_2Cl_2 at 298 K.

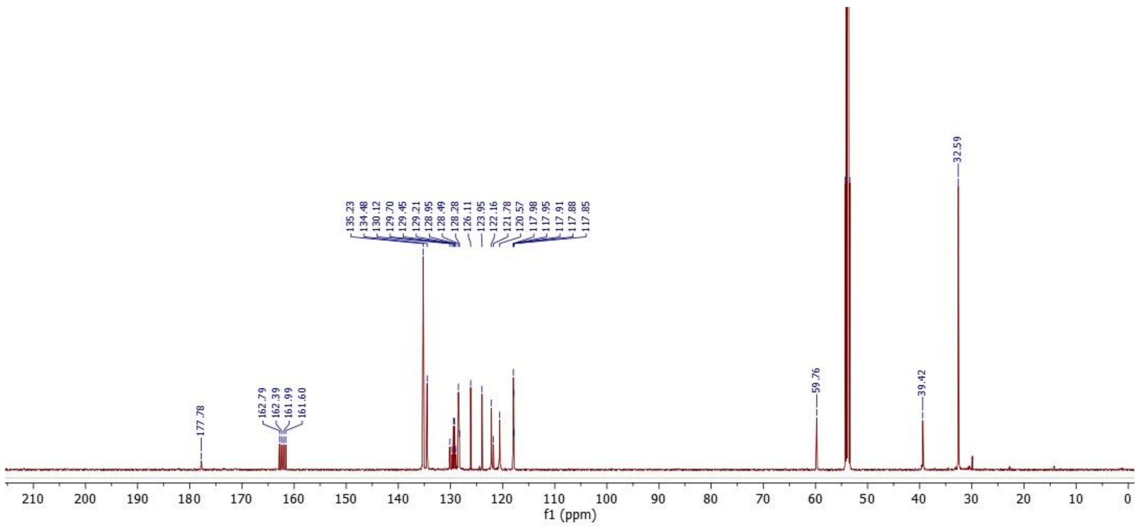

Figure S41. $^{13}C\{^{1}H\}$ NMR (126 MHz) of complex **6** in CD_2Cl_2 at 298 K.

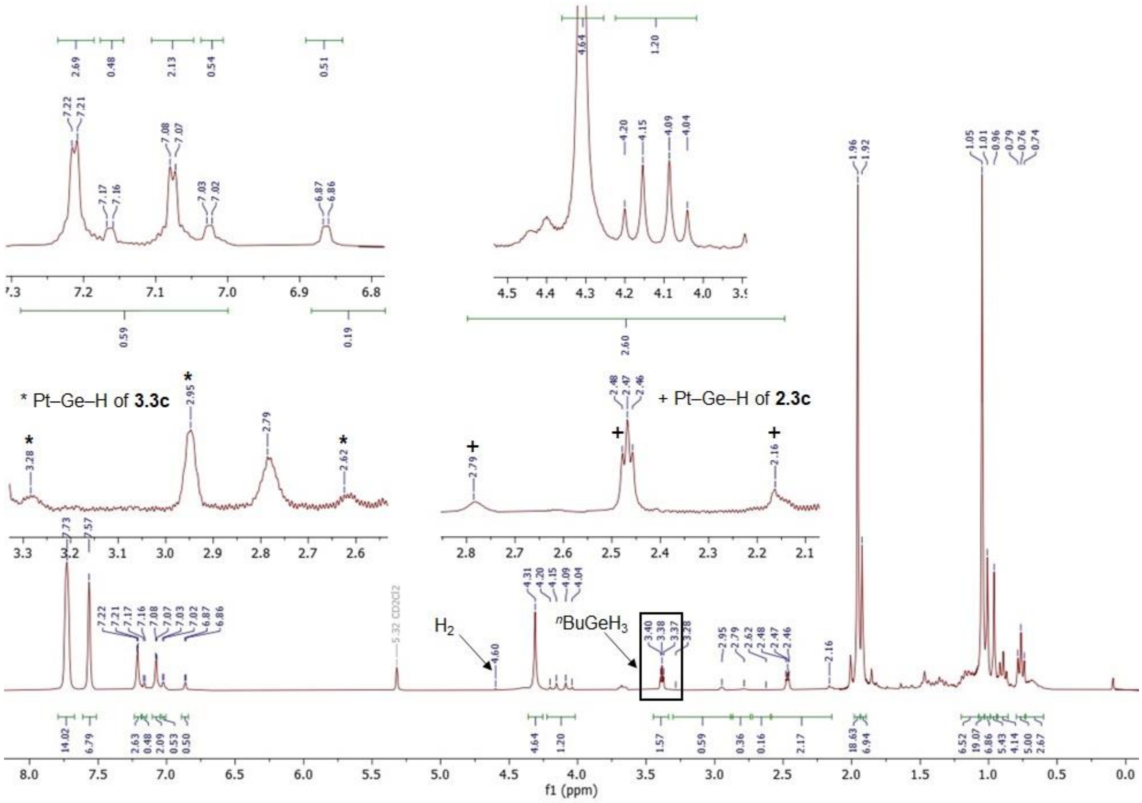

Figure S42. ^{1}H NMR (300 MHz) of the crude reaction mixture of complex **1.3** and ^{n}BuGeH$_3$ in CD_2Cl_2, containing amixture of complexes **2.3c** and **3.3c**.

14. Computational Details

Gibbs energy profiles

Calculations were performed using the M06 functional,[6] as implemented in Gaussian 09.[7] Geometry optimizations were performed in solution (solvent = dichloromethane, DCM, ε = 8.93) using the continuum SMD model[8] and basis set 1 (BS1). BS1 uses the double-ζ 6-31G(d,p)[8] basis set for the H, C, N and Ge atoms and the scalar relativistic Stuttgart−Dresden SDD pseudopotential[10] and its associated double-ζ basis set, complemented with a set of polarization functions, for the Pt atom.[11] The nature of the stationary points was confirmed by frequency analysis. Connections between the transition states and the minima were checked by perturbing the transition state geometry along the TS coordinate and optimizing until the corresponding minima. All energies in solution were corrected by single-point calculations with the larger basis set 2 (BS2) including triple-ζ def2TZVP basis set for the H, C, N, and Ge atoms and quadruple-ζ def2QZVP basis set for the Pt atom.[12] The scalar relativistic

Stuttgart−Dresden SDD pseudopotential and its associated basis set for Pt was used in the energy profile calculations. Gibbs energies in DCM were calculated at 298.15 K. Gibbs energy corrections were obtained based on vibrational frequencies of the BS1-optimised structures using the quasi-harmonic approximation. Thermal contributions to the Gibbs energies were corrected by employing the approximation described by Grimme, where entropic terms for frequencies below a cut-off of 100 cm^{-1} were calculated using the free-rotor approximation.[13] The GoodVibes program developed by Paton and Funes-Ardoiz was employed to introduce these corrections.[14] All reported energies in the main text correspond to M06/BS2 Gibbs energies in DCM solvent (1 M) at 298.15 K in kcal mol^{-1}. Structure visualization was performed with Chemcraft software.[15]

Energy Decomposition Analysis-Natural Orbital for Chemical Valence (EDA-NOCV) calculations

Geometry optimizations of the complexes were performed without symmetry constraints using the Gaussian09[7] optimizer together with Turbomole 7.1[16] energies and gradients at the BP86[17]/def2-TZVPP[12] level of theory using the D3 dispersion correction suggested by Grimme et al.[18] and the resolution-ofidentity (RI) approximation.[19] This level is denoted as RI-BP86-D3/def2-TZVPP and was chosen due to its good performance to understand the bonding situation of different transition metal complexes.[20] Vibrational analysis was performed to ensure that the optimized geometry corresponds to an energy minimum. Natural Bond Order (NBO) calculations were performed with the NBO6.0 program[201 at the same BP86-D3/def2-TZVPP level. The interaction ΔE_{int} between the selected fragments is analyzed with the help of the Energy Decomposition Analysis (EDA) method.[22] Within this approach, ΔE_{int} can be decomposed into the following physically meaningful terms:

$$\Delta E_{int} = \Delta E_{elstat} + \Delta E_{Pauli} + \Delta E_{orb} + \Delta E_{disp}$$

The term ΔE_{elstat} corresponds to the classical electrostatic interaction between the unperturbed charge distributions of the deformed reactants and is usually attractive. The Pauli repulsion ΔE_{Pauli} comprises the destabilizing interactions between occupied orbitals and is responsible for any steric repulsion. The orbital interaction ΔE_{orb} accounts for electronpair bonding, charge transfer (interaction between occupied orbitals on one moiety with unoccupied orbitals on the other, including HOMO-LUMO interactions), and polarization (emptyoccupied orbital mixing on one fragment due to the presence of another fragment). Finally, the ΔE_{disp} term takes into account the

interactions which are due to dispersion forces. Moreover, the NOCV (Natural Orbital for Chemical Valence)[23] extension of the EDA method has been also used to further partition the ΔE_{orb} term. The EDA-NOCV approach provides pairwise energy contributions for each pair of interacting orbitals to the total bond energy. The program package AMS 2020.10[24] was used for the EDA-NOCV calculations at the same BP86-D3 level, in conjunction with a triple-ζ-quality basis set using uncontracted Slater-type orbitals (STOs) augmented by two sets of polarization functions with a frozen-core approximation for the core electrons.[25] Auxiliary sets of s, p, d, f, and g STOs were used to fit the molecular densities and to represent the Coulomb and exchange potentials accurately in each SCF cycle.[26] Scalar relativistic effects were incorporated by applying the zerothorder regular approximation (ZORA).[27] This level of theory is denoted as ZORA-BP86-D3/TZ2P//RI-BP86-D3/def2-TZVPP.

15. Calculated energy profiles

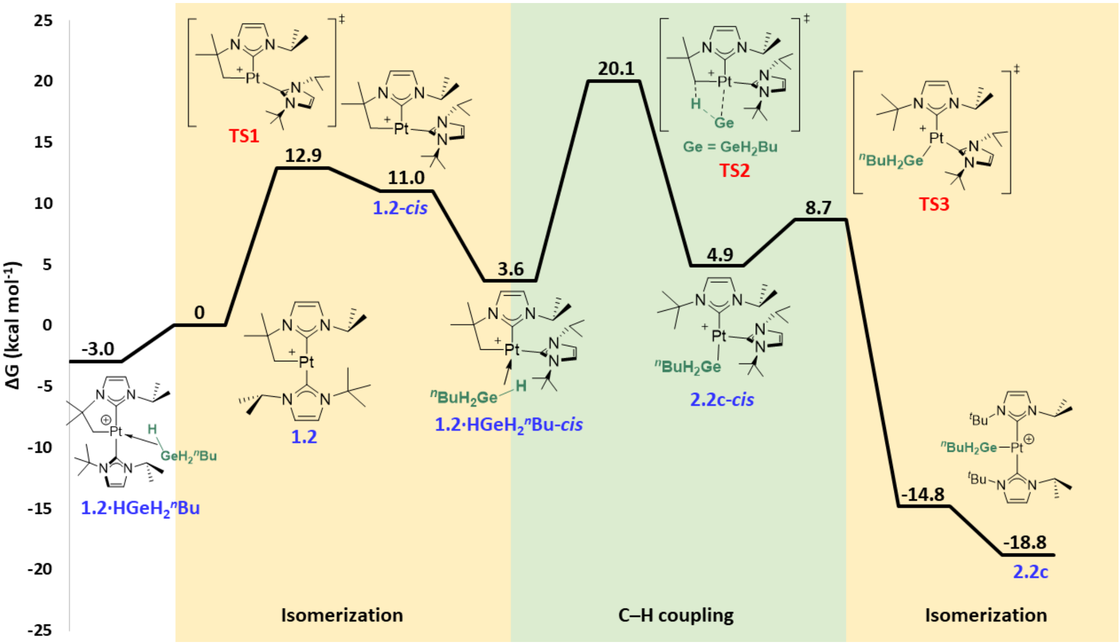

Figure S43. Gibbs energy profile in dichloromethane for the C–H coupling pathway (NHCs in *cis*) upon reaction between **1.2** and **^{n}BuGeH$_3$**. Gibbs energies at 298 K in kcal mol^{-1}.

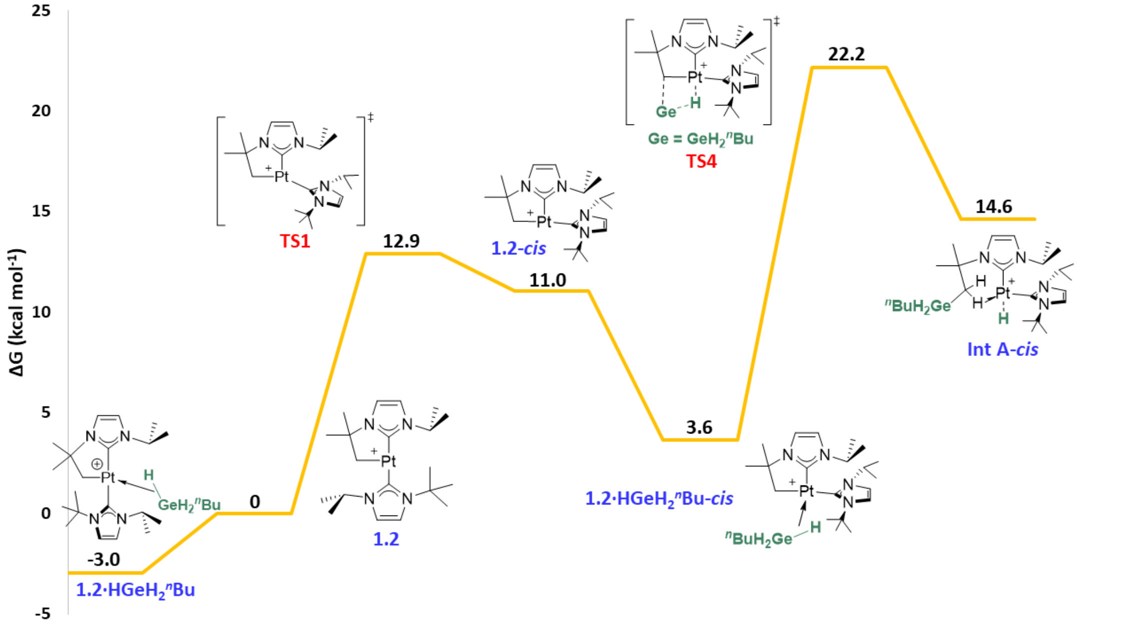

Figure S44. Gibbs energy profile in dichloromethane for the C–Ge coupling pathway (NHCs in *cis*) upon reaction between **1.2** and ^{n}**BuGeH$_3$**. Gibbs energies at 298 K in kcal mol^{-1}.

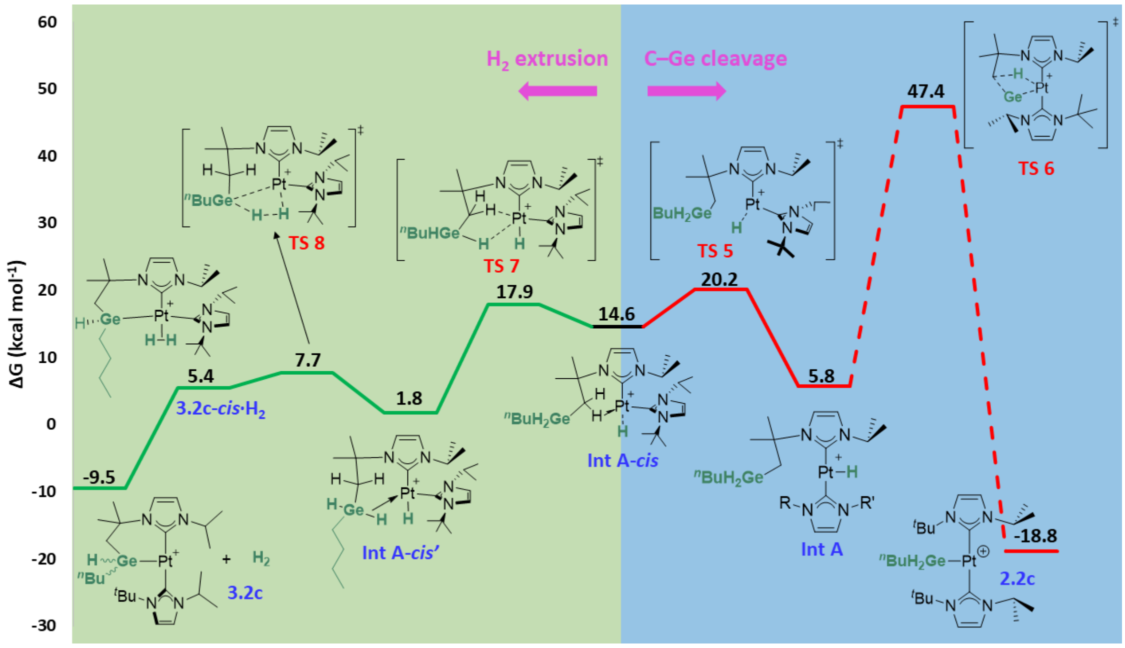

Figure S45. Gibbs energy profile in dichloromethane for the processes that might take place after C–Ge bond formation (NHCs in *cis*), namely H_2 extrusion (left) or C–Ge bond cleavage (right). Gibbs energies at 298 K in kcal mol^{-1}. The Gibbs energy of **1.2** + ^{n}BuGeH$_3$ has been taken as zero-energy.

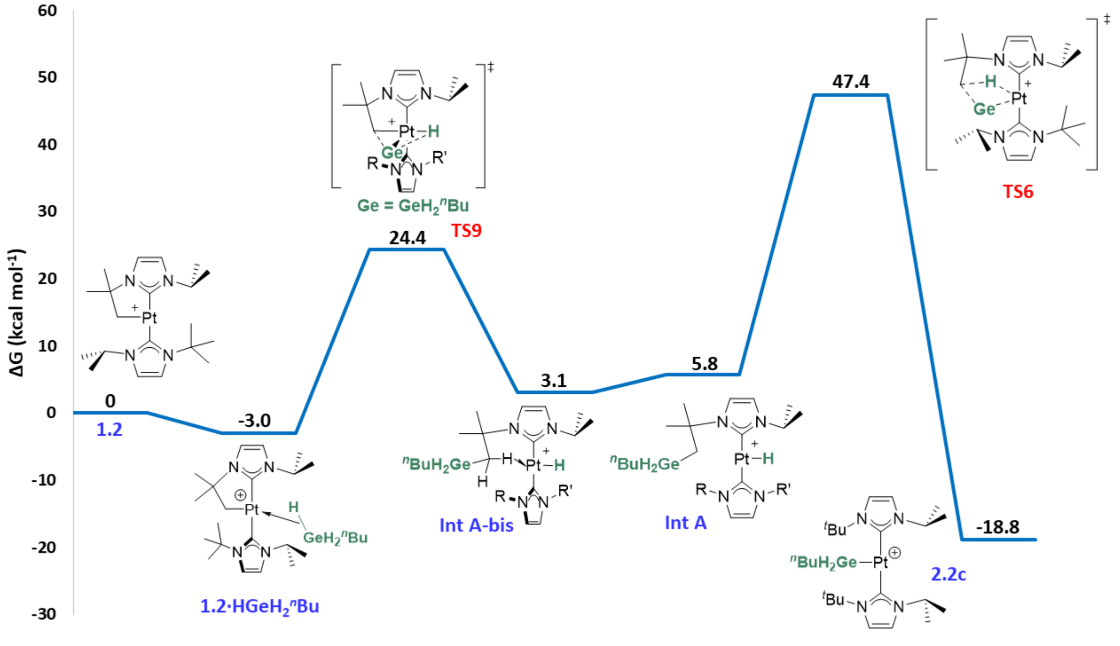

Figure S46. Gibbs energy profile in dichloromethane for the C–Ge coupling and cleavage pathway (NHCs in *trans*) upon reaction between **1.2** and **^{n}BuGeH$_3$**. Gibbs energies at 298 K in kcal mol^{-1}.

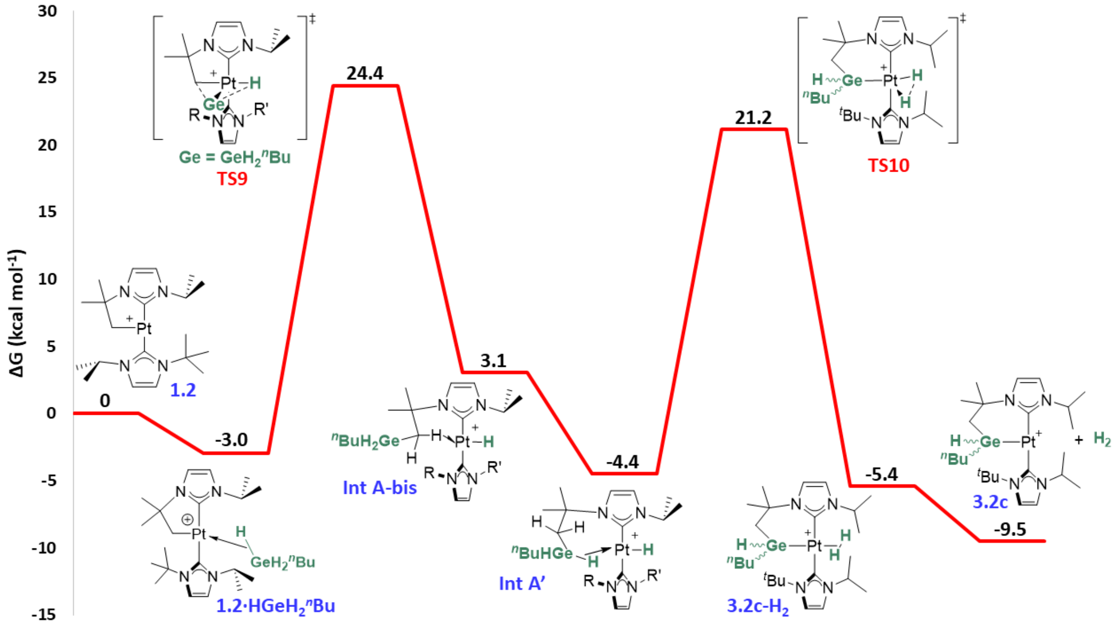

Figure S47. Gibbs energy profile in dichloromethane for the C–Ge coupling and H_2 extrusion pathway (NHCs in *trans*) upon reaction between **1.2** and **^{n}BuGeH$_3$**. Gibbs energies at 298 K in kcal mol^{-1}.

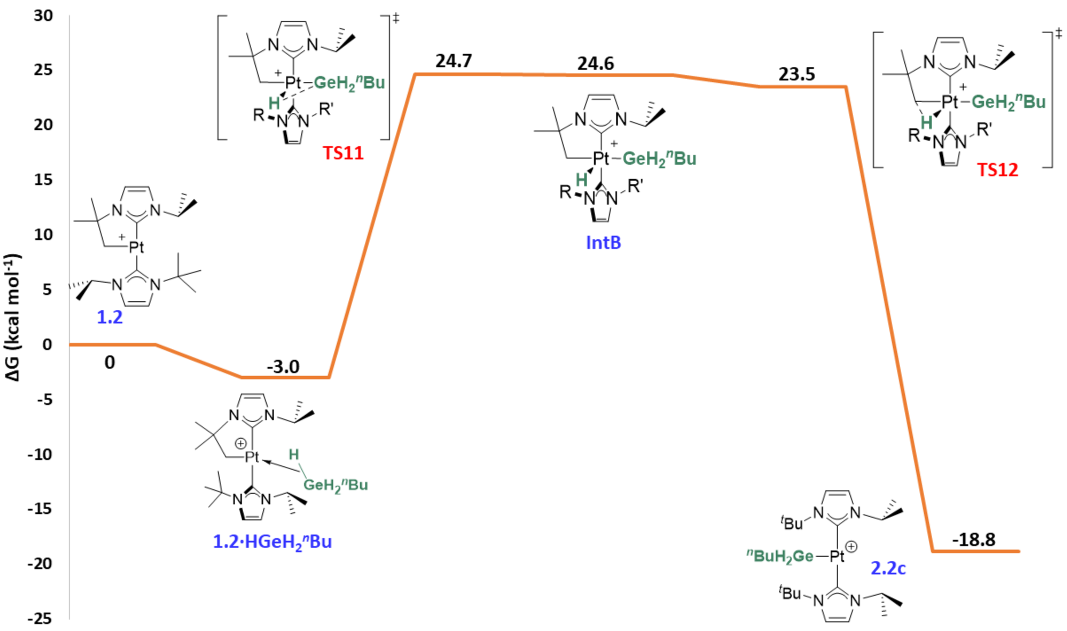

Figure S48. Gibbs energy profile in dichloromethane for the C–H coupling pathway (NHCs in *trans*) upon reaction between **1.2** and **^{n}BuGeH$_3$**. Gibbs energies at 298 K in kcal mol^{-1}.

16. Cartesian coordinates of the optimized structures

1.2

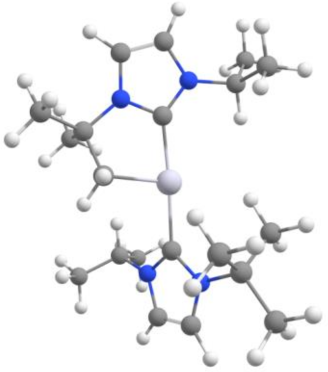

C	2.101646000	-0.086306000	0.032435000
C	4.342066000	0.009676000	0.034211000
H	5.319190000	0.421976000	0.244879000
C	3.987518000	-1.127287000	-0.618974000
H	4.593972000	-1.896935000	-1.076122000
C	1.664054000	-2.182734000	-1.100285000
C	2.127188000	-2.731488000	-2.441411000
H	2.294788000	-1.923457000	-3.162971000
H	1.359376000	-3.402188000	-2.844239000
H	3.052682000	-3.311606000	-2.338067000
C	0.326089000	-1.447695000	-1.263836000
H	-0.509537000	-2.156468000	-1.207805000
H	0.287278000	-0.925900000	-2.232141000
C	3.073820000	1.930702000	1.128224000
H	2.042168000	1.963200000	1.506253000

C	4.035923000	1.986352000	2.298348000
H	3.903756000	1.129471000	2.967797000
H	3.858558000	2.902474000	2.871412000
H	5.078762000	2.007937000	1.958473000
C	3.291073000	3.063401000	0.142121000
H	4.302346000	3.015698000	-0.281793000
H	3.179428000	4.029602000	0.646464000
H	2.567981000	3.016486000	-0.681220000
C	-1.987027000	0.074205000	0.110576000
C	-4.133345000	-0.550419000	0.396427000
H	-4.950522000	-1.201428000	0.674579000
C	-4.139059000	0.709381000	-0.098715000
H	-4.971799000	1.352467000	-0.336951000
C	-2.362288000	2.411179000	-0.800460000
C	-1.690816000	2.198245000	-2.151469000
H	-0.796670000	1.568354000	-2.058577000
H	-1.386912000	3.166983000	-2.565794000
H	-2.384552000	1.724946000	-2.857111000
C	-1.407100000	3.049997000	0.202318000
H	-1.878347000	3.147387000	1.187644000
H	-1.127745000	4.050603000	-0.148615000
H	-0.471666000	2.476928000	0.312109000
C	-3.567498000	3.325387000	-0.973290000
H	-4.279240000	2.933726000	-1.709435000
H	-3.213262000	4.294171000	-1.340824000
H	-4.089984000	3.496988000	-0.024488000
C	-2.375192000	-2.202610000	1.110264000
H	-1.287192000	-2.214071000	0.972942000
C	-3.000128000	-3.369625000	0.370737000
H	-4.085622000	-3.406096000	0.527365000
H	-2.581056000	-4.309576000	0.746701000
H	-2.808609000	-3.311821000	-0.706982000
C	-2.689047000	-2.217725000	2.594661000
H	-2.212012000	-1.373353000	3.106205000
H	-2.319365000	-3.146511000	3.043161000
H	-3.770847000	-2.166128000	2.771437000
C	1.573262000	-3.297073000	-0.066079000
H	2.551043000	-3.774000000	0.075803000
H	0.863753000	-4.062654000	-0.404178000
H	1.232964000	-2.907021000	0.900614000
N	-2.818312000	1.086777000	-0.268418000
N	-2.810799000	-0.922861000	0.518707000
N	3.170015000	0.633893000	0.433900000
N	2.608332000	-1.159940000	-0.614400000
Pt	0.084172000	0.051940000	0.096668000

1.2·HGeH$_2$^{n}Bu

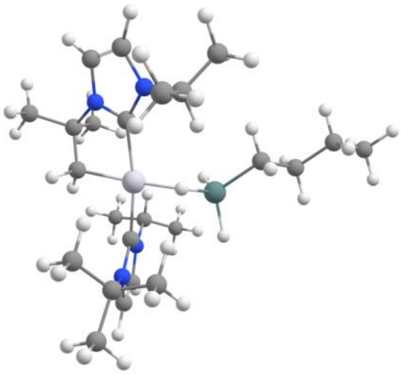

C	0.422007000	2.197207000	0.017160000
C	1.598226000	4.107784000	0.120571000
H	2.420100000	4.755284000	0.392202000
C	0.460751000	4.370245000	-0.571552000
H	0.106068000	5.284376000	-1.027267000
C	-1.560655000	2.889486000	-1.227920000
C	-2.487834000	4.084467000	-1.065843000
H	-2.560829000	4.388311000	-0.014671000
H	-3.489940000	3.806113000	-1.412712000
H	-2.159815000	4.946383000	-1.660664000
C	-2.088512000	1.668223000	-0.461334000
H	-2.853185000	1.159806000	-1.063880000
H	-2.571774000	2.003349000	0.469388000
C	2.537886000	2.112119000	1.362848000
H	2.366333000	1.039278000	1.224853000
C	3.959708000	2.442305000	0.955984000
H	4.128895000	2.246739000	-0.109319000
H	4.652732000	1.820711000	1.534066000
H	4.208710000	3.490542000	1.163488000
C	2.241741000	2.479414000	2.805869000
H	2.338606000	3.561978000	2.958810000
H	2.949590000	1.977670000	3.475059000
H	1.225450000	2.178043000	3.088938000
C	-1.813743000	-1.255016000	0.092593000
C	-3.066981000	-2.946533000	-0.719097000
H	-3.495965000	-3.591303000	-1.473115000
C	-3.205616000	-2.945972000	0.624610000
H	-3.792851000	-3.595607000	1.254003000
C	-2.303230000	-1.588896000	2.583986000
C	-2.605333000	-0.115541000	2.822920000
H	-1.868627000	0.535402000	2.336438000
H	-2.580092000	0.083392000	3.900839000
H	-3.603455000	0.143644000	2.447371000
C	-0.894443000	-1.946637000	3.039535000
H	-0.671579000	-2.998860000	2.822071000
H	-0.812568000	-1.795704000	4.122630000
H	-0.141208000	-1.318153000	2.552377000
C	-3.305406000	-2.423913000	3.370419000
H	-4.336727000	-2.237710000	3.048002000
H	-3.229337000	-2.140022000	4.425158000
H	-3.095668000	-3.497858000	3.304897000
C	-1.820768000	-1.567861000	-2.409465000
H	-1.090828000	-0.756033000	-2.295064000
C	-3.018045000	-1.067395000	-3.196565000

H	-3.763455000	-1.860930000	-3.335459000
H	-2.694765000	-0.737040000	-4.190370000
H	-3.505273000	-0.223988000	-2.693875000
C	-1.156221000	-2.758635000	-3.074204000
H	-0.323417000	-3.140388000	-2.472255000
H	-0.766779000	-2.462409000	-4.054516000
H	-1.872297000	-3.574920000	-3.233140000
C	-1.344361000	2.585572000	-2.705469000
H	-0.888087000	3.443367000	-3.216139000
H	-2.306578000	2.374661000	-3.188403000
H	-0.689098000	1.715580000	-2.838325000
N	-2.437322000	-1.901703000	1.119701000
N	-2.215260000	-1.905634000	-1.029011000
N	1.562732000	2.766949000	0.471976000
N	-0.249181000	3.189794000	-0.611309000
Pt	-0.539676000	0.395999000	0.093382000
Ge	1.475644000	-1.468524000	-0.296024000
H	0.825632000	-0.463149000	0.841448000
C	3.378391000	-1.249839000	0.079503000
H	3.656481000	-0.235795000	-0.242546000
H	3.531684000	-1.298877000	1.166338000
C	4.226939000	-2.291166000	-0.639303000
H	4.044923000	-2.239924000	-1.725269000
H	3.922658000	-3.304236000	-0.330143000
C	5.715725000	-2.111429000	-0.371612000
H	6.014341000	-1.096833000	-0.677129000
H	5.891970000	-2.164870000	0.713472000
C	6.567454000	-3.143837000	-1.088678000
H	6.424937000	-3.087529000	-2.175752000
H	7.635401000	-3.003442000	-0.886791000
H	6.301437000	-4.161984000	-0.775909000
H	0.945939000	-2.861805000	0.093887000
H	1.193575000	-1.191181000	-1.783313000

TS1

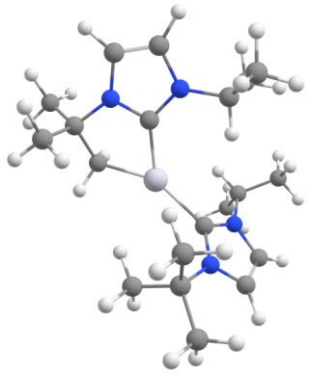

Pt	-0.210735000	-0.372348000	-0.492550000
N	-2.067064000	1.400743000	1.075212000
N	-3.067800000	-0.205382000	0.032032000
N	2.805712000	-0.837855000	0.468169000
N	2.596151000	1.017914000	-0.611355000
C	-1.857501000	0.348022000	0.252493000
C	-3.420247000	1.494219000	1.362416000
C	-4.048538000	0.478219000	0.715157000

C	1.888632000	-0.050108000	-0.163344000
C	3.922788000	0.908338000	-0.274086000
C	4.055171000	-0.260385000	0.405633000
H	-3.816514000	2.270278000	2.002462000
H	-5.091349000	0.194234000	0.685131000
H	4.658697000	1.654749000	-0.539286000
H	4.938543000	-0.706873000	0.835594000
C	2.028375000	2.141414000	-1.383791000
C	2.377640000	3.462039000	-0.725520000
H	3.455500000	3.662158000	-0.774159000
H	1.866370000	4.278436000	-1.247380000
H	2.070126000	3.477694000	0.327167000
C	2.488589000	2.062727000	-2.826567000
H	2.023834000	2.865682000	-3.409091000
H	3.577363000	2.178599000	-2.901417000
H	2.208331000	1.102737000	-3.275995000
C	-1.026830000	2.337940000	1.542531000
C	-3.079993000	-1.420004000	-0.797050000
C	-1.729049000	-1.333565000	-1.518808000
H	-1.830726000	-0.778393000	-2.463585000
H	-1.331392000	-2.336936000	-1.731421000
C	-1.154426000	2.577121000	3.033884000
H	-2.067464000	3.134519000	3.277480000
H	-1.159798000	1.633864000	3.591404000
H	-0.303573000	3.175193000	3.377002000
C	-1.094238000	3.621525000	0.735840000
H	-0.959540000	3.426303000	-0.335244000
H	-2.063869000	4.116074000	0.877964000
H	-0.310837000	4.314184000	1.063880000
C	-4.252528000	-1.385908000	-1.762729000
H	-5.211581000	-1.468882000	-1.235872000
H	-4.251720000	-0.460167000	-2.350277000
H	-4.177661000	-2.233508000	-2.453793000
C	-3.154345000	-2.639067000	0.111781000
H	-3.172221000	-3.552512000	-0.494349000
H	-2.282981000	-2.681688000	0.778426000
H	-4.064347000	-2.617410000	0.724485000
C	2.488825000	-2.133100000	1.160137000
C	3.776545000	-2.751446000	1.683918000
H	4.489415000	-2.960276000	0.877269000
H	4.260926000	-2.119433000	2.437434000
H	3.527886000	-3.703869000	2.163682000
C	1.852752000	-3.090168000	0.159251000
H	1.677675000	-4.056814000	0.646205000
H	0.884454000	-2.716601000	-0.203887000
H	2.513416000	-3.251005000	-0.701279000
C	1.553181000	-1.846898000	2.328746000
H	2.011742000	-1.136300000	3.027716000
H	1.351567000	-2.779362000	2.869192000
H	-0.078555000	1.820327000	1.338146000
H	0.595618000	-1.434856000	1.984762000
H	0.942152000	1.979603000	-1.337191000

1.2-*cis*

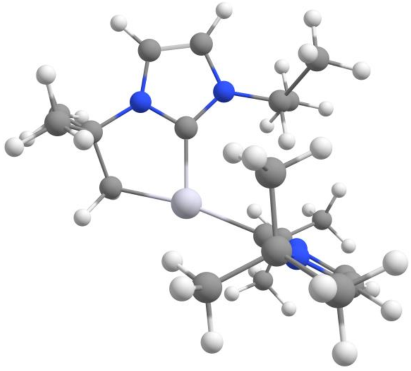

Pt	-0.352786000	-0.394314000	-0.811701000
N	-1.362240000	1.391537000	1.421477000
N	-2.881093000	0.287571000	0.344886000
N	2.473256000	-1.074515000	0.474072000
N	2.541818000	0.748186000	-0.675783000
C	-1.550491000	0.520334000	0.403070000
C	-2.588255000	1.694851000	1.994191000
C	-3.538899000	0.994898000	1.325214000
C	1.687947000	-0.210891000	-0.233563000
C	3.832933000	0.490836000	-0.270966000
C	3.789675000	-0.652511000	0.455396000
H	-2.673749000	2.385055000	2.821605000
H	-4.610485000	0.946025000	1.460673000
H	4.668655000	1.125831000	-0.530114000
H	4.589216000	-1.183127000	0.948811000
C	2.151188000	1.893325000	-1.520082000
C	2.792824000	3.171586000	-1.015509000
H	3.874767000	3.180076000	-1.196223000
H	2.363805000	4.027905000	-1.546960000
H	2.618523000	3.310423000	0.058283000
C	2.490432000	1.608462000	-2.971270000
H	2.183474000	2.450892000	-3.601003000
H	3.571953000	1.467997000	-3.096136000
H	1.979560000	0.706373000	-3.328531000
C	-0.087088000	2.014349000	1.827474000
C	-3.366362000	-0.696186000	-0.646183000
C	-2.218130000	-0.760167000	-1.659537000
H	-2.378387000	-0.009243000	-2.449568000
H	-2.187689000	-1.753228000	-2.134682000
C	0.105277000	1.895134000	3.327031000
H	-0.602708000	2.528796000	3.875690000
H	-0.019776000	0.859799000	3.664544000
H	1.115423000	2.225925000	3.590902000
C	-0.050441000	3.453834000	1.347180000
H	-0.187573000	3.514903000	0.260471000
H	-0.841720000	4.043703000	1.827637000
H	0.911317000	3.912500000	1.603859000
C	-4.668226000	-0.206670000	-1.258566000
H	-5.489247000	-0.192436000	-0.530220000
H	-4.551053000	0.799808000	-1.678163000
H	-4.956987000	-0.884351000	-2.070464000
C	-3.572452000	-2.029013000	0.061810000
H	-3.958494000	-2.769245000	-0.649077000
H	-2.626978000	-2.405260000	0.474409000

H	-4.297516000	-1.930883000	0.879879000
C	1.974491000	-2.290036000	1.198675000
C	3.144525000	-2.994076000	1.869900000
H	3.891136000	-3.334908000	1.142930000
H	3.633693000	-2.358392000	2.617266000
H	2.758021000	-3.877510000	2.388918000
C	1.336316000	-3.243976000	0.195660000
H	1.018390000	-4.156140000	0.714481000
H	0.449052000	-2.803336000	-0.281434000
H	2.053572000	-3.523983000	-0.585547000
C	0.980069000	-1.848935000	2.266309000
H	1.453427000	-1.144333000	2.962733000
H	0.646984000	-2.723716000	2.837505000
H	0.686610000	1.430254000	1.312839000
H	0.097696000	-1.370261000	1.824401000
H	1.060183000	1.960267000	-1.407006000

1.2·HGeH$_2$^{n}Bu-*cis*

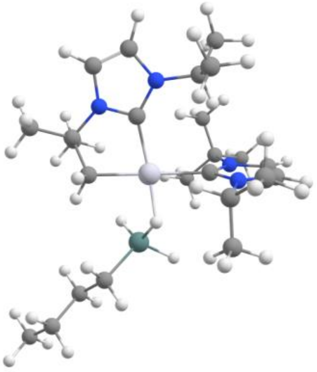

C	-2.535532000	-3.174011000	-0.599941000
C	-2.701927000	-3.021808000	0.732266000
H	-2.959605000	-3.896292000	-1.282993000
H	-3.309944000	-3.592222000	1.416593000
N	-1.647939000	-2.195535000	-1.000507000
N	-1.900510000	-1.960584000	1.131233000
C	-1.254011000	-1.429983000	0.051607000
Pt	0.082319000	0.208543000	-0.192596000
C	-1.624219000	3.981079000	-0.147656000
C	-2.809091000	3.341808000	-0.320097000
H	-1.394456000	5.034881000	-0.071994000
H	-3.807668000	3.737858000	-0.439093000
C	-1.213731000	1.770374000	-0.190010000
N	-2.544631000	1.982677000	-0.336563000
N	-0.661587000	3.001265000	-0.084682000
C	0.801520000	3.121005000	0.115277000
C	1.309438000	4.373937000	-0.580894000
C	1.072544000	3.199022000	1.613405000
C	1.380094000	1.842081000	-0.499454000
H	1.023932000	4.380390000	-1.639806000
H	0.938767000	5.293456000	-0.109591000
H	2.403877000	4.391161000	-0.521424000
H	0.719898000	2.292786000	2.123027000
H	2.149862000	3.300049000	1.792894000
H	0.569540000	4.067510000	2.058127000
H	2.395627000	1.698435000	-0.099605000

H	1.484248000	1.981754000	-1.588332000
C	-3.564414000	0.951473000	-0.612684000
C	-4.851931000	1.249440000	0.132184000
C	-3.782539000	0.835520000	-2.109725000
H	-4.667753000	1.459992000	1.191786000
H	-5.386996000	2.100472000	-0.306098000
H	-5.513817000	0.379093000	0.064850000
H	-2.854471000	0.563899000	-2.628014000
H	-4.535317000	0.065811000	-2.317363000
H	-4.146153000	1.786124000	-2.521304000
C	-1.102067000	-2.094485000	-2.369528000
C	0.033765000	-3.088965000	-2.543918000
C	-2.187093000	-2.301207000	-3.408943000
H	-0.338671000	-4.115515000	-2.431534000
H	0.466617000	-2.992429000	-3.546030000
H	0.831614000	-2.931893000	-1.807126000
H	-1.788052000	-2.054860000	-4.398699000
H	-2.520315000	-3.345912000	-3.443168000
H	-3.058714000	-1.664616000	-3.221832000
C	-1.846669000	-1.462563000	2.548199000
C	-0.402154000	-1.219475000	2.957645000
C	-2.667119000	-0.184683000	2.655922000
C	-2.436799000	-2.518261000	3.475581000
H	0.198665000	-2.130077000	2.837772000
H	0.053273000	-0.411065000	2.374905000
H	-0.377513000	-0.928052000	4.014157000
H	-3.700126000	-0.359198000	2.327755000
H	-2.691454000	0.147516000	3.700618000
H	-2.235260000	0.622583000	2.052802000
H	-2.310557000	-2.172241000	4.506842000
H	-3.510292000	-2.668185000	3.313720000
H	-1.919929000	-3.480527000	3.378584000
C	3.953115000	-0.847063000	-0.625566000
H	3.736971000	-0.047709000	-1.347531000
H	4.082659000	-1.773765000	-1.200568000
C	5.203151000	-0.522622000	0.182693000
H	5.052636000	0.422509000	0.730246000
H	5.368505000	-1.292323000	0.954432000
Ge	2.378536000	-1.071744000	0.504899000
H	1.197229000	-0.983440000	-0.687868000
H	2.420213000	-0.137072000	1.725379000
H	2.142313000	-2.525898000	0.956279000
C	6.448854000	-0.409221000	-0.685402000
H	6.276342000	0.351549000	-1.462059000
H	6.600170000	-1.359254000	-1.219920000
C	7.689083000	-0.061314000	0.118934000
H	8.581238000	0.006739000	-0.513765000
H	7.570251000	0.902601000	0.630847000
H	7.884935000	-0.819268000	0.888641000
H	-3.134301000	0.017672000	-0.233659000
H	-0.713027000	-1.068834000	-2.440881000

TS2

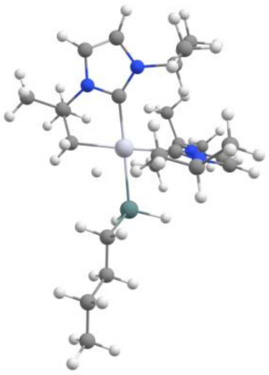

C	0.015955000	-3.699778000	0.588193000
C	0.454500000	-3.719057000	-0.685149000
H	-0.236107000	-4.516866000	1.247315000
H	0.671066000	-4.562253000	-1.320901000
N	-0.085275000	-2.371919000	0.968329000
N	0.614424000	-2.402502000	-1.086585000
C	0.259715000	-1.559402000	-0.069858000
Pt	0.018361000	0.512679000	-0.195733000
C	3.454080000	2.783357000	0.603613000
C	4.019006000	1.649333000	1.084383000
H	3.849565000	3.785367000	0.513349000
H	4.998016000	1.474646000	1.508605000
C	1.935024000	1.125657000	0.419439000
N	3.076876000	0.640462000	0.961466000
N	2.176434000	2.444225000	0.213518000
C	1.176141000	3.300656000	-0.455497000
C	1.232599000	4.705814000	0.128767000
C	1.482311000	3.329850000	-1.947758000
C	-0.187768000	2.674550000	-0.170558000
H	1.114224000	4.684502000	1.218531000
H	2.173289000	5.215061000	-0.113788000
H	0.417513000	5.302686000	-0.295729000
H	1.414823000	2.324176000	-2.381836000
H	0.765432000	3.979490000	-2.463690000
H	2.490860000	3.722737000	-2.127031000
H	-0.927831000	3.209484000	-0.780441000
H	-0.482546000	2.833786000	0.877259000
C	3.324644000	-0.745068000	1.403745000
C	4.595555000	-1.281193000	0.769009000
C	3.390690000	-0.807865000	2.919416000
H	4.609124000	-1.115487000	-0.313485000
H	5.485199000	-0.804669000	1.199498000
H	4.674121000	-2.357169000	0.958260000
H	2.473608000	-0.426993000	3.384148000
H	3.538081000	-1.845116000	3.242024000
H	4.237053000	-0.219904000	3.296719000
C	-0.612015000	-2.053693000	2.328187000
C	-0.560010000	-0.591897000	2.715427000
C	0.150897000	-2.868803000	3.363730000
H	0.471565000	-0.220464000	2.740321000
H	-1.151924000	0.056758000	2.062387000
H	-0.971411000	-0.503990000	3.727502000
H	-0.226447000	-2.622814000	4.361689000
H	0.044529000	-3.948935000	3.232771000

H	1.219445000	-2.618083000	3.332537000
C	1.104489000	-2.020005000	-2.461036000
C	0.037523000	-1.220638000	-3.198212000
C	2.400812000	-1.235393000	-2.323959000
C	1.386955000	-3.284209000	-3.263985000
H	-0.911402000	-1.771402000	-3.228958000
H	-0.133970000	-0.240576000	-2.738147000
H	0.369648000	-1.055362000	-4.229967000
H	3.168378000	-1.852346000	-1.840017000
H	2.763661000	-0.957949000	-3.320849000
H	2.264003000	-0.318461000	-1.740108000
H	1.755719000	-2.978390000	-4.248651000
H	2.161468000	-3.907846000	-2.802826000
H	0.483641000	-3.885005000	-3.421503000
C	-3.648748000	0.578037000	0.549292000
H	-3.447415000	1.656382000	0.637700000
H	-3.443021000	0.135242000	1.534723000
C	-5.095338000	0.325770000	0.145167000
H	-5.287472000	0.769861000	-0.845149000
H	-5.265867000	-0.757327000	0.024196000
Ge	-2.372140000	-0.172320000	-0.741188000
H	-1.067482000	1.432167000	-0.931803000
H	-2.879281000	0.206015000	-2.159709000
H	-2.452625000	-1.721348000	-0.679244000
C	-6.100952000	0.880092000	1.145869000
H	-5.925567000	1.959922000	1.268962000
H	-5.912785000	0.428749000	2.132230000
C	-7.539334000	0.631939000	0.726184000
H	-8.254531000	1.035834000	1.451897000
H	-7.752291000	1.098233000	-0.244741000
H	-7.741111000	-0.442435000	0.623764000
H	2.459502000	-1.316178000	1.042519000
H	-1.663683000	-2.378600000	2.311294000

2.2c-cis

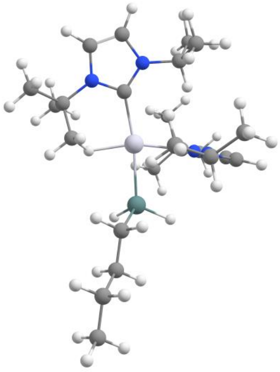

C	-0.058925000	-3.665794000	0.841424000
C	0.361053000	-3.780937000	-0.431689000
H	-0.301127000	-4.431221000	1.562593000
H	0.567734000	-4.672342000	-1.001056000
N	-0.161223000	-2.312784000	1.126977000
N	0.509572000	-2.499289000	-0.941402000
C	0.166366000	-1.588319000	0.020404000
Pt	0.037791000	0.373771000	-0.204542000
C	3.450818000	2.864263000	0.515338000

C	4.045967000	1.728716000	0.945921000
H	3.832128000	3.873286000	0.451399000
H	5.036505000	1.560378000	1.345205000
C	1.954545000	1.182166000	0.298060000
N	3.118804000	0.710215000	0.804925000
N	2.166759000	2.517594000	0.133144000
C	1.197551000	3.454829000	-0.489737000
C	1.247464000	4.792044000	0.237534000
C	1.561133000	3.617195000	-1.960123000
C	-0.202555000	2.874836000	-0.349161000
H	1.070154000	4.664163000	1.312034000
H	2.206180000	5.301901000	0.095114000
H	0.468425000	5.448111000	-0.166069000
H	1.512755000	2.651628000	-2.480772000
H	0.863566000	4.310026000	-2.445006000
H	2.575161000	4.019781000	-2.067071000
H	-0.946883000	3.581137000	-0.736321000
H	-0.475533000	2.674314000	0.695631000
C	3.389958000	-0.680865000	1.212787000
C	4.671896000	-1.181527000	0.571562000
C	3.447740000	-0.779470000	2.726575000
H	4.686907000	-0.991556000	-0.507078000
H	5.552146000	-0.702223000	1.017787000
H	4.766734000	-2.260443000	0.736468000
H	2.529435000	-0.403232000	3.192247000
H	3.589041000	-1.823925000	3.027600000
H	4.292407000	-0.200852000	3.122099000
C	-0.685090000	-1.901191000	2.466017000
C	-0.643908000	-0.416998000	2.764753000
C	0.084549000	-2.644783000	3.549785000
H	0.384928000	-0.038278000	2.794209000
H	-1.219786000	0.189701000	2.059621000
H	-1.079449000	-0.275534000	3.760885000
H	-0.283202000	-2.325241000	4.530235000
H	-0.026663000	-3.731381000	3.501051000
H	1.153614000	-2.400671000	3.490675000
C	1.006190000	-2.225071000	-2.341707000
C	-0.023093000	-1.425139000	-3.129050000
C	2.336817000	-1.491875000	-2.257706000
C	1.226248000	-3.549889000	-3.062058000
H	-0.991973000	-1.939845000	-3.145624000
H	-0.162341000	-0.417762000	-2.719623000
H	0.328856000	-1.326139000	-4.162636000
H	3.069337000	-2.094280000	-1.705784000
H	2.723637000	-1.327563000	-3.270367000
H	2.232207000	-0.517049000	-1.767319000
H	1.571509000	-3.323678000	-4.076171000
H	1.997979000	-4.163542000	-2.583343000
H	0.301686000	-4.132303000	-3.150671000
C	-3.617980000	0.579553000	0.538057000
H	-3.344941000	1.632959000	0.705837000
H	-3.496816000	0.065296000	1.503191000
C	-5.057196000	0.465742000	0.055069000
H	-5.167292000	0.979143000	-0.914317000
H	-5.300471000	-0.592228000	-0.140927000

Ge	-2.320323000	-0.198770000	-0.730800000
H	-0.375279000	2.022627000	-1.080525000
H	-2.736738000	0.344874000	-2.135873000
H	-2.652143000	-1.723267000	-0.800840000
C	-6.070347000	1.035239000	1.040165000
H	-5.828782000	2.092097000	1.232661000
H	-5.959856000	0.519858000	2.006777000
C	-7.501277000	0.911269000	0.546183000
H	-8.221125000	1.323541000	1.262671000
H	-7.638152000	1.442296000	-0.405072000
H	-7.769122000	-0.139512000	0.373858000
H	2.537350000	-1.258981000	0.834293000
H	-1.735012000	-2.231653000	2.472252000

TS3

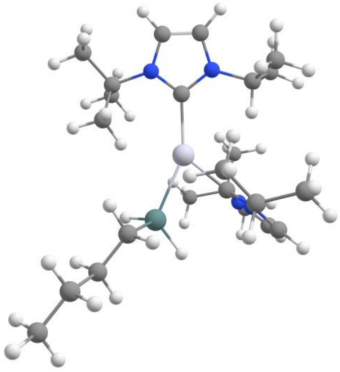

C	-0.933885000	-3.756541000	0.680698000
C	-0.518938000	-3.894607000	-0.591722000
H	-1.349185000	-4.486550000	1.359287000
H	-0.497425000	-4.778930000	-1.207053000
N	-0.747077000	-2.430462000	1.041717000
N	-0.087559000	-2.651207000	-1.030205000
C	-0.251846000	-1.734386000	-0.024139000
Pt	0.192668000	0.179844000	-0.102507000
C	3.688238000	2.748544000	0.462166000
C	4.168045000	1.594726000	0.978831000
H	4.163429000	3.712037000	0.353820000
H	5.130636000	1.363679000	1.413975000
C	2.048601000	1.207151000	0.309666000
N	3.152513000	0.662763000	0.877330000
N	2.384249000	2.501896000	0.064719000
C	1.540023000	3.491821000	-0.668767000
C	1.890254000	4.897313000	-0.196492000
C	1.819659000	3.341847000	-2.159129000
C	0.073704000	3.229964000	-0.369549000
H	1.795059000	4.987072000	0.892308000
H	2.901798000	5.197516000	-0.491014000
H	1.195536000	5.606393000	-0.659347000
H	1.550553000	2.336525000	-2.508518000
H	1.231519000	4.073691000	-2.725501000
H	2.881198000	3.513177000	-2.375301000
H	-0.537362000	4.002208000	-0.851785000
H	-0.133358000	3.243106000	0.707219000
C	3.278557000	-0.731414000	1.342323000
C	4.431138000	-1.420676000	0.634644000

C	3.423376000	-0.771734000	2.852392000
H	4.347055000	-1.323846000	-0.453942000
H	5.397204000	-1.003791000	0.946041000
H	4.434061000	-2.486700000	0.888100000
H	2.579628000	-0.278401000	3.349913000
H	3.468682000	-1.811986000	3.193728000
H	4.349083000	-0.277598000	3.173871000
C	-1.103820000	-2.013035000	2.431790000
C	-0.756060000	-0.587300000	2.803138000
C	-0.415581000	-2.949292000	3.416470000
H	0.330586000	-0.433204000	2.811252000
H	-1.207278000	0.165634000	2.152149000
H	-1.125771000	-0.418490000	3.821333000
H	-0.684301000	-2.657384000	4.437038000
H	-0.692882000	-3.999285000	3.292375000
H	0.674359000	-2.864042000	3.313097000
C	0.593423000	-2.440130000	-2.359613000
C	-0.108388000	-1.355124000	-3.164914000
C	2.052859000	-2.086818000	-2.100093000
C	0.535013000	-3.735599000	-3.159541000
H	-1.170159000	-1.593112000	-3.305495000
H	-0.021329000	-0.370644000	-2.691065000
H	0.360528000	-1.294033000	-4.154009000
H	2.542323000	-2.877147000	-1.515868000
H	2.580790000	-1.988952000	-3.056192000
H	2.144216000	-1.136809000	-1.559448000
H	1.019934000	-3.556012000	-4.124650000
H	1.074167000	-4.554279000	-2.669621000
H	-0.496594000	-4.048866000	-3.359320000
C	-3.326033000	0.928713000	0.620507000
H	-2.831875000	1.856054000	0.944314000
H	-3.394370000	0.273734000	1.500711000
C	-4.713859000	1.214547000	0.059131000
H	-4.634239000	1.882168000	-0.814082000
H	-5.164861000	0.281253000	-0.317797000
Ge	-2.193896000	0.042573000	-0.718596000
H	-0.272399000	2.273112000	-0.806067000
H	-2.294972000	0.851250000	-2.043841000
H	-2.805330000	-1.354675000	-1.009991000
C	-5.650809000	1.839728000	1.085268000
H	-5.200196000	2.770213000	1.463273000
H	-5.730367000	1.167082000	1.952876000
C	-7.031142000	2.121980000	0.518202000
H	-7.698832000	2.564615000	1.266173000
H	-6.975808000	2.816892000	-0.329874000
H	-7.504452000	1.200138000	0.155329000
H	2.331439000	-1.207000000	1.050061000
H	-2.193931000	-2.144979000	2.499646000

2.2c

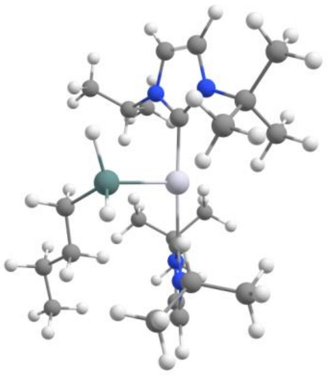

C	2.331304000	-0.449900000	0.105972000
C	4.095140000	-1.833971000	-0.135101000
H	4.611728000	-2.778352000	-0.236027000
C	4.573152000	-0.568365000	-0.090685000
H	5.591650000	-0.217017000	-0.147333000
C	3.565456000	1.771621000	0.142017000
C	5.025505000	2.200146000	0.117177000
H	5.591591000	1.779128000	0.956579000
H	5.063184000	3.291078000	0.203847000
H	5.518302000	1.924896000	-0.822711000
C	2.940383000	2.227256000	1.455722000
H	3.041354000	3.315354000	1.549010000
H	3.443245000	1.758642000	2.310574000
C	1.816797000	-2.904884000	0.021264000
H	0.816124000	-2.468755000	-0.089599000
C	2.085625000	-3.843072000	-1.139267000
H	2.058909000	-3.310097000	-2.097082000
H	1.318254000	-4.624755000	-1.159102000
H	3.059306000	-4.339412000	-1.041469000
C	1.907376000	-3.594935000	1.369502000
H	2.912714000	-4.003466000	1.534945000
H	1.194095000	-4.426058000	1.413442000
H	1.677688000	-2.899039000	2.186254000
C	-1.518760000	0.856209000	0.704049000
C	-3.428415000	2.053728000	0.626426000
H	-4.087644000	2.856152000	0.326062000
C	-3.631735000	1.020361000	1.477166000
H	-4.511191000	0.762278000	2.046696000
C	-2.228586000	-0.911914000	2.388553000
C	-0.984098000	-0.680927000	3.238878000
H	-0.076111000	-0.596955000	2.618964000
H	-0.839289000	-1.530783000	3.916617000
H	-1.083419000	0.231113000	3.839668000
C	-2.074843000	-2.143599000	1.506060000
H	-2.974308000	-2.300101000	0.895826000
H	-1.930289000	-3.028528000	2.137906000
H	-1.207935000	-2.046841000	0.842126000
C	-3.424707000	-1.105955000	3.309249000
H	-3.595968000	-0.234385000	3.951967000
H	-3.221005000	-1.965513000	3.956448000
H	-4.341673000	-1.326363000	2.749356000
C	-1.468022000	2.966783000	-0.662016000
H	-0.543614000	2.487966000	-1.003686000
C	-1.114010000	4.170197000	0.192124000

H	-2.016685000	4.643392000	0.600058000
H	-0.586083000	4.915417000	-0.413878000
H	-0.464660000	3.882099000	1.028135000
C	-2.321527000	3.331006000	-1.859819000
H	-2.624486000	2.439730000	-2.422097000
H	-1.746188000	3.979762000	-2.529397000
H	-3.224023000	3.881015000	-1.564717000
C	2.845273000	2.378608000	-1.056110000
H	3.273637000	2.007343000	-1.995874000
H	2.955052000	3.469616000	-1.032503000
H	1.775252000	2.138788000	-1.038446000
N	-2.453154000	0.295417000	1.528657000
N	-2.132250000	1.943126000	0.168196000
N	2.723934000	-1.743706000	-0.018168000
N	3.485198000	0.276581000	0.056294000
Pt	0.401977000	0.201459000	0.356223000
Ge	-0.003028000	-0.280250000	-1.987678000
H	1.868105000	1.984766000	1.501196000
C	-1.452749000	-1.577713000	-2.255128000
H	-1.415201000	-1.854599000	-3.319366000
H	-1.219274000	-2.488316000	-1.684657000
C	-2.826605000	-1.038375000	-1.887343000
H	-3.049142000	-0.137335000	-2.483838000
H	-2.829303000	-0.710290000	-0.836295000
C	-3.946973000	-2.052187000	-2.072849000
H	-4.007777000	-2.344287000	-3.132027000
H	-3.692978000	-2.970421000	-1.519395000
C	-5.284426000	-1.513902000	-1.594682000
H	-5.558219000	-0.598743000	-2.136414000
H	-6.095248000	-2.237982000	-1.733057000
H	-5.244035000	-1.258610000	-0.525675000
H	-0.396271000	1.048733000	-2.684823000
H	1.305119000	-0.783319000	-2.647744000

TS4

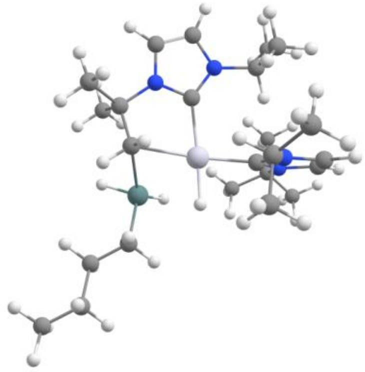

C	3.094852000	-2.577327000	1.200181000
C	3.170235000	-2.794935000	-0.128786000
H	3.696381000	-2.962221000	2.010981000
H	3.869707000	-3.396082000	-0.688630000
N	2.032902000	-1.719087000	1.403961000
N	2.136648000	-2.086675000	-0.730880000
C	1.445963000	-1.394442000	0.219784000
Pt	-0.089526000	-0.070684000	0.146676000
C	1.092762000	3.996617000	-0.107054000

C	2.356890000	3.526163000	-0.210540000
H	0.719155000	5.010765000	-0.103342000
H	3.297076000	4.052579000	-0.297950000
C	0.988329000	1.740377000	-0.057416000
N	2.277951000	2.143755000	-0.183403000
N	0.270937000	2.890443000	-0.003320000
C	-1.202738000	2.932378000	0.121330000
C	-1.568127000	3.913979000	1.233521000
C	-1.771066000	3.416776000	-1.205641000
C	-1.683539000	1.537377000	0.540672000
H	-1.096899000	3.626396000	2.180999000
H	-1.264071000	4.938292000	0.989563000
H	-2.654955000	3.917254000	1.373432000
H	-1.486586000	2.753556000	-2.031936000
H	-2.864145000	3.472033000	-1.158304000
H	-1.392858000	4.421412000	-1.428921000
H	-2.757653000	1.616842000	0.766299000
H	-1.289180000	1.294642000	1.546182000
C	3.466701000	1.274085000	-0.101308000
C	4.507789000	1.673690000	-1.128825000
C	4.018122000	1.302688000	1.312220000
H	4.080699000	1.733129000	-2.136426000
H	4.965021000	2.640650000	-0.886499000
H	5.309818000	0.927405000	-1.137930000
H	3.241934000	1.041162000	2.040847000
H	4.844251000	0.588158000	1.409970000
H	4.399921000	2.301291000	1.560814000
C	1.509791000	-1.370058000	2.745049000
C	0.484987000	-2.405110000	3.176902000
C	2.632403000	-1.251867000	3.757582000
H	0.957107000	-3.392126000	3.267949000
H	0.071483000	-2.135419000	4.155358000
H	-0.340522000	-2.477244000	2.460240000
H	2.226225000	-0.844262000	4.689191000
H	3.066861000	-2.230763000	3.994932000
H	3.433671000	-0.588536000	3.414558000
C	1.972202000	-2.096707000	-2.228819000
C	0.538461000	-1.800890000	-2.622560000
C	2.910930000	-1.057970000	-2.827740000
C	2.331612000	-3.488345000	-2.744284000
H	-0.161273000	-2.515230000	-2.171795000
H	0.238035000	-0.788987000	-2.336074000
H	0.459147000	-1.882802000	-3.712757000
H	3.948077000	-1.231908000	-2.513614000
H	2.873167000	-1.120041000	-3.921749000
H	2.617876000	-0.041300000	-2.537776000
H	2.081541000	-3.537655000	-3.809601000
H	3.399158000	-3.714942000	-2.657210000
H	1.758884000	-4.264991000	-2.223637000
C	-3.706742000	-1.046167000	0.138516000
H	-3.478023000	-0.860023000	1.197779000
H	-3.403865000	-2.081403000	-0.071037000
C	-5.188262000	-0.837664000	-0.140302000
H	-5.460991000	0.210904000	0.065315000
H	-5.398059000	-0.995436000	-1.210840000

Ge	-2.547592000	0.119546000	-0.918173000
H	-0.973020000	-1.403416000	0.393680000
H	-3.420905000	1.250658000	-1.509880000
H	-1.888053000	-0.541525000	-2.145705000
C	-6.076397000	-1.758935000	0.685020000
H	-5.866775000	-1.595125000	1.753283000
H	-5.798339000	-2.803888000	0.479220000
C	-7.554573000	-1.550605000	0.406166000
H	-8.183146000	-2.212955000	1.012260000
H	-7.855855000	-0.517319000	0.623275000
H	-7.788399000	-1.744890000	-0.648821000
H	3.099924000	0.267557000	-0.329928000
H	1.021828000	-0.394009000	2.618582000

Int A-*cis*

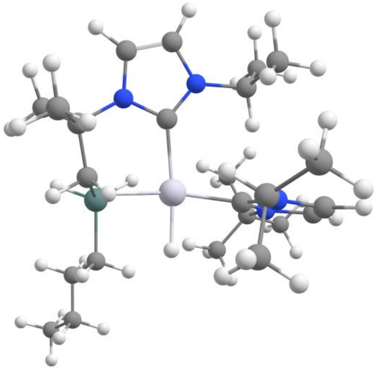

C	3.395287000	-2.548286000	0.748395000
C	2.805190000	-3.093248000	-0.337154000
H	4.313212000	-2.811139000	1.254208000
H	3.131877000	-3.918621000	-0.948899000
N	2.581547000	-1.514965000	1.171594000
N	1.624439000	-2.398769000	-0.567775000
C	1.494413000	-1.415018000	0.366596000
Pt	0.099528000	-0.023159000	0.621132000
C	0.933071000	3.775779000	-1.116719000
C	2.015733000	3.170171000	-1.656385000
H	0.593208000	4.798962000	-1.189946000
H	2.796433000	3.561718000	-2.293907000
C	0.904228000	1.613287000	-0.462212000
N	1.979417000	1.846354000	-1.252127000
N	0.256126000	2.807305000	-0.397284000
C	-0.919015000	3.070980000	0.467287000
C	-0.420767000	3.742932000	1.745237000
C	-1.883371000	3.994872000	-0.266099000
C	-1.600318000	1.743251000	0.819377000
H	0.253210000	3.077494000	2.299340000
H	0.117684000	4.670498000	1.516151000
H	-1.270495000	3.993624000	2.391072000
H	-2.114307000	3.625749000	-1.272120000
H	-2.818877000	4.069808000	0.299921000
H	-1.473874000	5.006811000	-0.354834000
H	-2.412187000	1.976516000	1.526235000
H	-0.974221000	1.186708000	1.597437000
C	3.035516000	0.869529000	-1.574981000
C	3.336970000	0.872030000	-3.061424000

C	4.266051000	1.147494000	-0.732725000
H	2.423803000	0.755552000	-3.656247000
H	3.838379000	1.797215000	-3.370678000
H	4.010980000	0.040586000	-3.296266000
H	4.008880000	1.176240000	0.332797000
H	5.013510000	0.360092000	-0.887909000
H	4.719878000	2.109045000	-1.004903000
C	2.789400000	-0.759012000	2.427433000
C	2.110631000	-1.487232000	3.574570000
C	4.265938000	-0.549712000	2.699570000
H	2.564953000	-2.476629000	3.717100000
H	2.234147000	-0.919991000	4.503879000
H	1.038975000	-1.618369000	3.388161000
H	4.375647000	0.137777000	3.544613000
H	4.762098000	-1.487968000	2.976740000
H	4.789506000	-0.120231000	1.838601000
C	0.665946000	-2.717350000	-1.686747000
C	-0.700125000	-3.034618000	-1.095910000
C	0.602123000	-1.536711000	-2.645284000
C	1.164894000	-3.939229000	-2.445837000
H	-0.629754000	-3.854363000	-0.369990000
H	-1.132654000	-2.163919000	-0.594836000
H	-1.377326000	-3.341667000	-1.901641000
H	1.579070000	-1.373245000	-3.117777000
H	-0.126613000	-1.753082000	-3.435858000
H	0.291345000	-0.616049000	-2.137769000
H	0.446586000	-4.151410000	-3.244772000
H	2.139993000	-3.768620000	-2.917084000
H	1.222248000	-4.827768000	-1.806162000
C	-3.620348000	-0.718828000	0.351842000
H	-3.581355000	-0.512944000	1.431921000
H	-3.055258000	-1.645774000	0.189581000
C	-5.062610000	-0.877893000	-0.112510000
H	-5.617388000	0.057147000	0.066580000
H	-5.093852000	-1.037582000	-1.202774000
Ge	-2.691695000	0.756092000	-0.537423000
H	-0.609934000	-1.163633000	1.524893000
H	-3.764771000	1.785914000	-0.967141000
H	-1.903675000	0.357979000	-1.801644000
C	-5.780083000	-2.027026000	0.582321000
H	-5.733708000	-1.871097000	1.671106000
H	-5.233435000	-2.962468000	0.387722000
C	-7.225575000	-2.167388000	0.138230000
H	-7.730461000	-2.999287000	0.642485000
H	-7.795309000	-1.254039000	0.354293000
H	-7.291385000	-2.345705000	-0.943111000
H	2.615765000	-0.102005000	-1.293404000
H	2.300781000	0.211481000	2.261974000

TS5

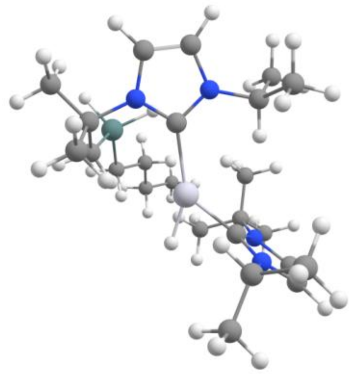

C	4.282699000	1.723768000	-0.226437000
C	3.456696000	2.676168000	0.258075000
H	5.357964000	1.711908000	-0.334323000
H	3.690922000	3.649160000	0.659740000
N	3.490450000	0.655624000	-0.601408000
N	2.157152000	2.191323000	0.172534000
C	2.189869000	0.932074000	-0.345944000
Pt	0.724205000	-0.349336000	-0.585054000
C	-1.677892000	-3.430247000	1.356073000
C	-0.940133000	-3.025549000	2.416742000
H	-2.456278000	-4.175502000	1.298501000
H	-0.952220000	-3.347576000	3.448699000
C	-0.260528000	-1.839191000	0.628131000
N	-0.081220000	-2.048763000	1.951274000
N	-1.247529000	-2.698104000	0.261996000
C	-1.820634000	-2.774411000	-1.118622000
C	-0.756258000	-3.348830000	-2.047435000
C	-3.034058000	-3.692208000	-1.115773000
C	-2.231348000	-1.370607000	-1.565013000
H	0.136627000	-2.708270000	-2.074029000
H	-0.455609000	-4.352616000	-1.722051000
H	-1.154800000	-3.418743000	-3.066419000
H	-3.820481000	-3.343126000	-0.435741000
H	-3.451778000	-3.711550000	-2.127827000
H	-2.765376000	-4.721206000	-0.849990000
H	-2.771828000	-1.453938000	-2.517863000
H	-1.337774000	-0.755859000	-1.790008000
C	0.915431000	-1.358868000	2.790968000
C	0.244923000	-0.734269000	3.998916000
C	2.027718000	-2.319368000	3.165903000
H	-0.592625000	-0.091462000	3.701306000
H	-0.133136000	-1.500249000	4.687165000
H	0.971071000	-0.126676000	4.550104000
H	2.485575000	-2.757270000	2.270926000
H	2.804376000	-1.792832000	3.731624000
H	1.646931000	-3.134759000	3.794039000
C	4.001420000	-0.570920000	-1.248266000
C	4.429081000	-0.257119000	-2.669248000
C	5.116207000	-1.180317000	-0.421054000
H	5.265825000	0.453062000	-2.677944000
H	4.759367000	-1.173709000	-3.170082000
H	3.602922000	0.173951000	-3.246833000
H	5.406429000	-2.142817000	-0.855478000
H	6.007667000	-0.540764000	-0.410778000

H	4.798637000	-1.350668000	0.614055000
C	0.936067000	2.959910000	0.603687000
C	-0.023447000	3.077311000	-0.573066000
C	0.293459000	2.249350000	1.787663000
C	1.356054000	4.358523000	1.033003000
H	0.476010000	3.530208000	-1.438792000
H	-0.425281000	2.100880000	-0.868784000
H	-0.864280000	3.720102000	-0.284624000
H	1.006492000	2.168611000	2.618282000
H	-0.571199000	2.830188000	2.132677000
H	-0.052866000	1.243203000	1.516352000
H	0.452782000	4.909157000	1.317189000
H	2.021209000	4.342902000	1.903978000
H	1.840707000	4.909876000	0.218695000
C	-3.479228000	1.519519000	-1.038268000
H	-4.346545000	1.558743000	-1.711126000
H	-2.591388000	1.708665000	-1.660607000
C	-3.591629000	2.576922000	0.051825000
H	-4.438597000	2.350189000	0.719930000
H	-2.691752000	2.539442000	0.690382000
Ge	-3.329369000	-0.300241000	-0.332506000
H	1.024797000	0.464383000	-1.892085000
H	-4.724867000	-0.943500000	-0.157277000
H	-2.669364000	-0.250625000	1.068906000
C	-3.749166000	3.987274000	-0.498582000
H	-4.684317000	4.047768000	-1.075611000
H	-2.938610000	4.181650000	-1.219586000
C	-3.736826000	5.044122000	0.591827000
H	-3.857894000	6.055034000	0.186257000
H	-4.545833000	4.877220000	1.315103000
H	-2.791418000	5.023268000	1.153487000
H	1.317405000	-0.564965000	2.146464000
H	3.141356000	-1.253738000	-1.263035000

Int A

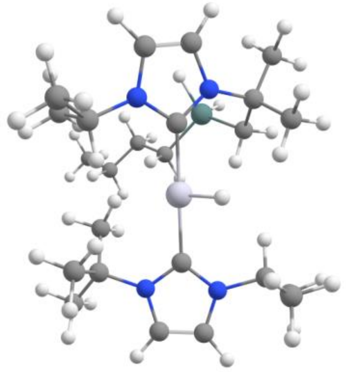

C	1.284556000	-0.740893000	1.274262000
C	3.042731000	-0.447564000	2.659224000
H	3.601948000	-0.016549000	3.478010000
C	3.384231000	-1.399757000	1.761117000
H	4.305417000	-1.951805000	1.658611000
C	1.861131000	-1.726520000	-1.520008000
H	-0.876660000	-1.924809000	0.790122000
H	0.796377000	-1.461388000	-1.436416000
C	-2.505484000	-0.202266000	-0.240957000

C	-4.534372000	-0.532170000	-1.159110000
H	-5.314083000	-1.065085000	-1.684651000
C	-4.523257000	0.717985000	-0.639911000
H	-5.292436000	1.475611000	-0.638246000
N	1.755710000	-0.060118000	2.350603000
N	2.304803000	-1.572237000	0.910614000
N	-3.287896000	-1.077624000	-0.917671000
N	-3.270170000	0.913933000	-0.087094000
Pt	-0.606309000	-0.452888000	0.483219000
C	1.025431000	1.010104000	3.053419000
H	-0.004090000	0.931137000	2.680278000
C	1.025772000	0.771857000	4.550620000
H	0.383364000	1.514232000	5.036687000
H	0.644295000	-0.225987000	4.793813000
H	2.030815000	0.875709000	4.977782000
C	1.599766000	2.363606000	2.676368000
H	1.617967000	2.503410000	1.586962000
H	0.992912000	3.163408000	3.117125000
H	2.625657000	2.471452000	3.051450000
C	2.311874000	-2.495230000	-0.276284000
C	3.723723000	-3.024698000	-0.499363000
H	3.711334000	-3.647248000	-1.400226000
H	4.455472000	-2.223074000	-0.656207000
H	4.064458000	-3.655509000	0.329683000
C	1.394248000	-3.679859000	0.008664000
H	0.351178000	-3.368911000	0.132671000
H	1.441107000	-4.384736000	-0.829993000
H	1.712119000	-4.206707000	0.917196000
C	-2.886868000	-2.444059000	-1.308727000
H	-1.791721000	-2.440079000	-1.246225000
C	-3.449463000	-3.451714000	-0.324096000
H	-3.114200000	-3.240396000	0.698245000
H	-4.546997000	-3.434734000	-0.338416000
H	-3.122906000	-4.462590000	-0.593351000
C	-3.300668000	-2.739762000	-2.737401000
H	-2.869396000	-3.698187000	-3.045561000
H	-4.389671000	-2.825589000	-2.836574000
H	-2.946189000	-1.966078000	-3.427507000
C	-2.823825000	2.138277000	0.640861000
C	-1.401334000	2.477336000	0.211268000
H	-1.100565000	3.429937000	0.665077000
H	-0.650831000	1.744577000	0.572720000
H	-1.319828000	2.563610000	-0.878438000
C	-3.727997000	3.305401000	0.271546000
H	-3.737381000	3.483191000	-0.810473000
H	-4.756019000	3.154631000	0.619265000
H	-3.348707000	4.208331000	0.762000000
C	-2.902454000	1.868532000	2.137954000
H	-3.939057000	1.676040000	2.440218000
H	-2.294064000	0.997033000	2.410389000
H	-2.535947000	2.740080000	2.695271000
H	1.964944000	-2.390539000	-2.387079000
Ge	2.823555000	-0.043980000	-1.878750000
H	3.641161000	-0.156154000	-3.186536000
C	1.572697000	1.453487000	-1.991401000

H	1.031691000	1.469100000	-1.031540000
H	0.825741000	1.240672000	-2.770477000
C	2.263280000	2.787459000	-2.239345000
H	3.058667000	2.940333000	-1.490271000
H	2.775311000	2.763531000	-3.215100000
C	1.314816000	3.978055000	-2.198603000
H	0.497476000	3.813630000	-2.918224000
H	0.841190000	4.021816000	-1.204462000
C	2.013533000	5.293723000	-2.493944000
H	1.323218000	6.143915000	-2.451856000
H	2.817969000	5.482705000	-1.771042000
H	2.467516000	5.283847000	-3.493358000
H	3.820256000	0.271189000	-0.734528000

TS6

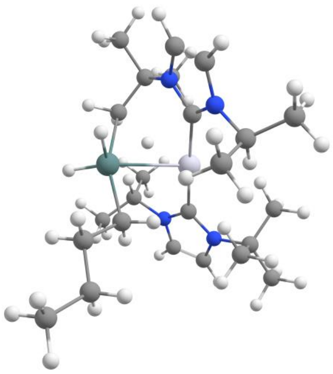

C	-2.071123000	0.161824000	-0.772823000
C	-4.069593000	1.204910000	-0.769292000
H	-4.762942000	2.027125000	-0.877455000
C	-4.285492000	-0.087166000	-0.424312000
H	-5.208036000	-0.596500000	-0.189748000
C	-1.690483000	-2.292082000	0.926917000
H	-0.697390000	-1.405909000	0.278145000
H	-1.057562000	-3.171993000	0.750299000
C	1.993358000	-0.558868000	-0.741360000
C	4.038457000	-1.462700000	-0.401282000
H	4.761041000	-2.176648000	-0.031648000
C	4.207509000	-0.350235000	-1.150462000
H	5.115126000	0.079931000	-1.543912000
N	-2.711035000	1.336503000	-0.987678000
N	-3.052332000	-0.715524000	-0.427123000
N	2.684768000	-1.571077000	-0.152165000
N	2.952733000	0.198959000	-1.356194000
Pt	-0.054692000	-0.210215000	-0.726551000
C	-2.023412000	2.605262000	-1.282537000
H	-1.016198000	2.300251000	-1.598810000
C	-2.707056000	3.341096000	-2.417915000
H	-2.114392000	4.220703000	-2.691612000
H	-2.806911000	2.703498000	-3.303165000
H	-3.704303000	3.695286000	-2.128486000
C	-1.925092000	3.440356000	-0.018697000
H	-1.446276000	2.876050000	0.793204000
H	-1.335666000	4.344809000	-0.208625000
H	-2.922344000	3.749254000	0.320663000
C	-2.796504000	-2.159813000	-0.133570000

C	-4.068777000	-2.792334000	0.410401000
H	-3.849830000	-3.830134000	0.683614000
H	-4.424967000	-2.273913000	1.309214000
H	-4.868656000	-2.808918000	-0.339885000
C	-2.403112000	-2.854010000	-1.434394000
H	-1.461763000	-2.455326000	-1.831380000
H	-2.277613000	-3.928599000	-1.255166000
H	-3.188212000	-2.718426000	-2.188588000
C	2.089293000	-2.690018000	0.596853000
H	1.073915000	-2.359064000	0.833364000
C	2.011126000	-3.924579000	-0.281349000
H	1.428592000	-3.723412000	-1.189211000
H	3.014440000	-4.254845000	-0.579949000
H	1.532054000	-4.746052000	0.263889000
C	2.833667000	-2.935506000	1.895204000
H	2.283872000	-3.671789000	2.492014000
H	3.838918000	-3.338858000	1.721276000
H	2.925662000	-2.015970000	2.485173000
C	2.695641000	1.473146000	-2.105233000
C	2.108279000	2.498577000	-1.141597000
H	1.945557000	3.447735000	-1.667096000
H	1.145795000	2.157263000	-0.737132000
H	2.791962000	2.678319000	-0.301478000
C	4.006555000	2.006542000	-2.665090000
H	4.721113000	2.260256000	-1.873423000
H	4.474479000	1.298111000	-3.359000000
H	3.791027000	2.924552000	-3.222140000
C	1.745403000	1.193564000	-3.264131000
H	2.166498000	0.439964000	-3.940813000
H	0.765859000	0.834990000	-2.914888000
H	1.584647000	2.118059000	-3.831948000
H	-2.132268000	-2.414073000	1.924707000
Ge	-0.737293000	-0.477956000	2.042700000
H	-0.343897000	-1.517191000	3.128868000
C	0.610709000	0.948844000	2.224109000
H	0.448941000	1.706740000	1.449087000
H	1.608829000	0.515299000	2.075931000
C	0.455935000	1.541234000	3.619745000
H	-0.559124000	1.954584000	3.744335000
H	0.564654000	0.757264000	4.387306000
C	1.477060000	2.644398000	3.883303000
H	2.488731000	2.229763000	3.759313000
H	1.368559000	3.420704000	3.110496000
C	1.328600000	3.257500000	5.264367000
H	2.069503000	4.045655000	5.440104000
H	0.333268000	3.701596000	5.395299000
H	1.456365000	2.500132000	6.048602000
H	-2.146334000	0.109415000	2.276184000

TS7

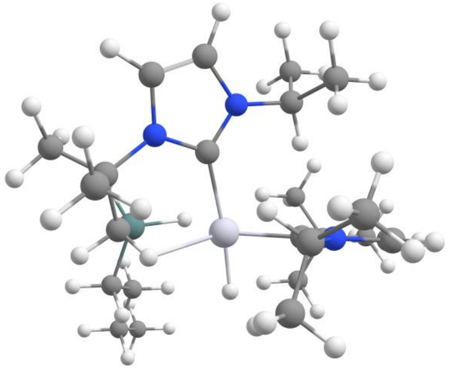

C	3.574110000	1.975110000	-1.295633000
C	3.078706000	2.927676000	-0.477209000
H	4.518551000	1.924646000	-1.817539000
H	3.528326000	3.855676000	-0.163178000
N	2.620681000	0.978561000	-1.373654000
N	1.817885000	2.517475000	-0.061263000
C	1.539130000	1.304481000	-0.620304000
Pt	-0.031109000	0.118747000	-0.426311000
C	0.955995000	-3.617997000	1.422043000
C	1.907691000	-2.896383000	2.055582000
H	0.716513000	-4.669572000	1.485930000
H	2.647774000	-3.192908000	2.785712000
C	0.780307000	-1.482729000	0.706548000
N	1.784672000	-1.592144000	1.607933000
N	0.262298000	-2.739654000	0.606301000
C	-0.769541000	-3.177009000	-0.374859000
C	-0.041202000	-3.779876000	-1.574138000
C	-1.665890000	-4.225796000	0.274240000
C	-1.605533000	-1.984745000	-0.846966000
H	0.602368000	-3.029725000	-2.052522000
H	0.580817000	-4.631227000	-1.272402000
H	-0.769594000	-4.133896000	-2.313080000
H	-2.083178000	-3.873439000	1.224084000
H	-2.495267000	-4.454753000	-0.404357000
H	-1.130718000	-5.163408000	0.458682000
H	-2.417229000	-2.375226000	-1.477834000
H	-1.030570000	-1.387555000	-1.594660000
C	2.665096000	-0.497673000	2.053928000
C	2.590888000	-0.339066000	3.560489000
C	4.080486000	-0.732395000	1.560579000
H	1.556841000	-0.192298000	3.894377000
H	3.001352000	-1.216533000	4.075569000
H	3.181224000	0.531008000	3.869327000
H	4.099840000	-0.873927000	0.472619000
H	4.708711000	0.130621000	1.808418000
H	4.525166000	-1.618336000	2.031648000
C	2.724729000	-0.223175000	-2.231606000
C	1.985375000	0.004296000	-3.538920000
C	4.173302000	-0.591625000	-2.480194000
H	2.446406000	0.831511000	-4.094637000
H	2.041778000	-0.897184000	-4.160189000
H	0.929026000	0.241818000	-3.370237000
H	4.207200000	-1.561811000	-2.986549000
H	4.665291000	0.135983000	-3.137432000

H	4.750230000	-0.670970000	-1.551897000
C	0.938312000	3.339751000	0.851474000
C	-0.313368000	3.770143000	0.098334000
C	0.592122000	2.538050000	2.098713000
C	1.699085000	4.587666000	1.281724000
H	-0.046573000	4.289932000	-0.830420000
H	-0.955275000	2.919522000	-0.149187000
H	-0.885842000	4.462401000	0.726920000
H	1.500259000	2.278753000	2.657734000
H	-0.041116000	3.154067000	2.748102000
H	0.043847000	1.618858000	1.861195000
H	1.052842000	5.156774000	1.957992000
H	2.618504000	4.345376000	1.827814000
H	1.942528000	5.238582000	0.434095000
C	-4.165065000	-0.134608000	-0.498749000
H	-4.915351000	-0.924401000	-0.640233000
H	-3.792978000	0.137931000	-1.497900000
C	-4.766147000	1.077718000	0.198196000
H	-5.089395000	0.806850000	1.216771000
H	-3.989215000	1.850686000	0.326069000
Ge	-2.644830000	-0.888480000	0.458218000
H	-0.744448000	1.215658000	-1.374189000
H	-2.997784000	-1.776821000	1.664004000
H	-1.786873000	0.296068000	1.011420000
C	-5.945163000	1.674011000	-0.558857000
H	-6.717520000	0.900466000	-0.688687000
H	-5.617260000	1.947484000	-1.573478000
C	-6.535205000	2.884974000	0.141812000
H	-7.380686000	3.307819000	-0.412567000
H	-6.894128000	2.623776000	1.145989000
H	-5.784840000	3.678006000	0.258604000
H	2.259837000	0.401833000	1.578018000
H	2.233589000	-1.023469000	-1.661237000

Int A-*cis’*

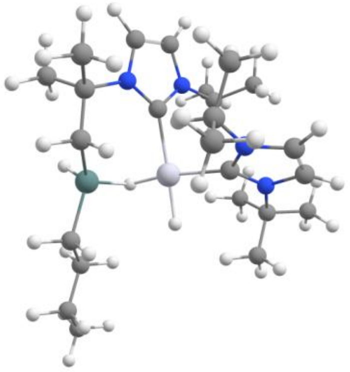

C	-3.206665000	1.104211000	2.195645000
C	-3.234288000	2.254832000	1.490965000
H	-3.867122000	0.726231000	2.963420000
H	-3.938343000	3.070320000	1.536982000
N	-2.108045000	0.396817000	1.752295000
N	-2.153345000	2.241123000	0.620467000
C	-1.443287000	1.086075000	0.791137000
Pt	0.253697000	0.402772000	-0.165653000
C	-1.223147000	-3.626807000	-1.050133000

C	-2.118119000	-2.873939000	-1.725414000
H	-1.158827000	-4.700241000	-0.945339000
H	-2.981813000	-3.154855000	-2.312277000
C	-0.620046000	-1.467182000	-0.782650000
N	-1.731394000	-1.559561000	-1.554380000
N	-0.304378000	-2.757376000	-0.474576000
C	0.848759000	-3.305239000	0.315528000
C	0.283583000	-4.128122000	1.471826000
C	1.668148000	-4.194068000	-0.617014000
C	1.733209000	-2.215533000	0.914390000
H	-0.298867000	-3.492848000	2.150710000
H	-0.353638000	-4.950479000	1.129363000
H	1.112318000	-4.565285000	2.040459000
H	1.992742000	-3.645244000	-1.508649000
H	2.558528000	-4.555066000	-0.089578000
H	1.098192000	-5.070064000	-0.944208000
H	2.580316000	-2.729058000	1.390766000
H	1.212002000	-1.676556000	1.716899000
C	-2.445981000	-0.416002000	-2.143909000
C	-2.534992000	-0.561278000	-3.650840000
C	-3.806236000	-0.240634000	-1.494168000
H	-1.543600000	-0.706027000	-4.094750000
H	-3.172512000	-1.407211000	-3.937779000
H	-2.975532000	0.346319000	-4.079681000
H	-3.723163000	-0.158626000	-0.402893000
H	-4.281964000	0.673235000	-1.871264000
H	-4.467926000	-1.085007000	-1.728268000
C	-1.735761000	-0.920547000	2.298984000
C	-1.276483000	-0.782068000	3.737752000
C	-2.874812000	-1.907186000	2.122438000
H	-2.088366000	-0.434524000	4.389239000
H	-0.938559000	-1.754433000	4.115297000
H	-0.441937000	-0.074137000	3.814012000
H	-2.540036000	-2.910665000	2.410621000
H	-3.735962000	-1.650261000	2.752364000
H	-3.208951000	-1.945020000	1.077205000
C	-1.806658000	3.445575000	-0.221575000
C	-0.730384000	4.239802000	0.508124000
C	-1.355194000	3.033922000	-1.615454000
C	-3.051045000	4.313493000	-0.381523000
H	-1.072600000	4.512548000	1.514351000
H	0.200576000	3.670382000	0.596419000
H	-0.518529000	5.163409000	-0.043641000
H	-2.172872000	2.547025000	-2.163420000
H	-1.075791000	3.938895000	-2.167368000
H	-0.485950000	2.365962000	-1.600251000
H	-2.814208000	5.116918000	-1.087109000
H	-3.893683000	3.743027000	-0.792216000
H	-3.359976000	4.789454000	0.555596000
C	3.878331000	0.229761000	0.640926000
H	4.702442000	-0.439987000	0.926177000
H	3.431801000	0.609559000	1.570345000
C	4.387776000	1.369486000	-0.230280000
H	4.796829000	0.967856000	-1.172239000
H	3.547112000	2.021264000	-0.520076000

Ge	2.540828000	-0.865421000	-0.249349000
H	0.948808000	1.772731000	0.312466000
H	3.103276000	-1.489799000	-1.544842000
H	1.576022000	0.222563000	-1.201101000
C	5.453813000	2.210845000	0.459100000
H	6.298842000	1.563669000	0.739458000
H	5.045176000	2.602171000	1.403338000
C	5.941671000	3.356189000	-0.410384000
H	6.710262000	3.953613000	0.092899000
H	6.372659000	2.983938000	-1.349124000
H	5.116203000	4.030485000	-0.673787000
H	-1.813117000	0.444403000	-1.905216000
H	-0.888143000	-1.243855000	1.689309000

TS8

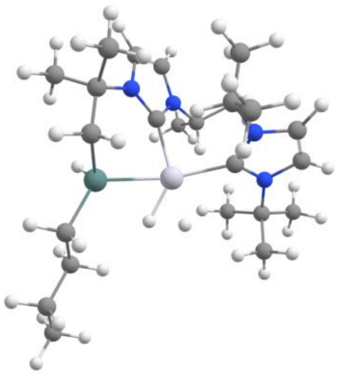

C	3.805461000	0.403608000	-1.711159000
C	3.896111000	1.609999000	-1.111141000
H	4.528003000	-0.155987000	-2.288583000
H	4.727664000	2.296036000	-1.073287000
N	2.532552000	-0.066129000	-1.456802000
N	2.678821000	1.861140000	-0.492515000
C	1.824898000	0.818702000	-0.711641000
Pt	-0.206875000	0.472512000	-0.134456000
C	0.569127000	-3.539889000	1.356202000
C	1.439913000	-2.817208000	2.091881000
H	0.404989000	-4.606851000	1.326027000
H	2.168486000	-3.124769000	2.828988000
C	0.274591000	-1.374441000	0.802187000
N	1.244337000	-1.496110000	1.744875000
N	-0.155469000	-2.650036000	0.576659000
C	-1.265606000	-3.171434000	-0.301832000
C	-0.638325000	-4.069470000	-1.364570000
C	-2.213472000	-3.981663000	0.582171000
C	-2.056372000	-2.052543000	-0.964452000
H	0.066074000	-3.508650000	-1.991579000
H	-0.107624000	-4.923005000	-0.924870000
H	-1.428907000	-4.465037000	-2.012972000
H	-2.652026000	-3.356432000	1.369044000
H	-3.026876000	-4.373603000	-0.038382000
H	-1.722345000	-4.837330000	1.056014000
H	-2.986607000	-2.490959000	-1.348164000
H	-1.517775000	-1.648687000	-1.833798000
C	1.957200000	-0.380376000	2.387765000
C	1.604660000	-0.307201000	3.861121000

C	3.450980000	-0.481131000	2.144379000
H	0.519551000	-0.241454000	4.003201000
H	1.976305000	-1.182420000	4.408749000
H	2.064804000	0.583569000	4.304700000
H	3.680960000	-0.569880000	1.075434000
H	3.946811000	0.418983000	2.528232000
H	3.885483000	-1.345280000	2.663167000
C	2.019949000	-1.330815000	-2.014524000
C	1.855447000	-1.209991000	-3.517830000
C	2.900737000	-2.495803000	-1.605372000
H	2.821545000	-1.057604000	-4.015694000
H	1.415286000	-2.130762000	-3.918561000
H	1.195713000	-0.372007000	-3.773840000
H	2.425324000	-3.436319000	-1.907313000
H	3.884244000	-2.449695000	-2.090054000
H	3.049771000	-2.523837000	-0.518533000
C	2.364836000	3.199653000	0.126300000
C	1.623486000	4.033067000	-0.912653000
C	1.547332000	3.045705000	1.401124000
C	3.668877000	3.899290000	0.495730000
H	2.213477000	4.111344000	-1.834220000
H	0.648208000	3.600172000	-1.161212000
H	1.459408000	5.044885000	-0.523457000
H	2.130646000	2.545409000	2.185214000
H	1.291102000	4.047154000	1.766273000
H	0.613050000	2.494226000	1.244401000
H	3.419467000	4.804801000	1.059163000
H	4.300580000	3.269141000	1.134271000
H	4.244289000	4.214070000	-0.381631000
C	-4.034264000	0.444801000	-0.608056000
H	-4.872457000	-0.267248000	-0.620327000
H	-3.793492000	0.671270000	-1.657576000
C	-4.421297000	1.711197000	0.141008000
H	-4.639229000	1.471279000	1.195082000
H	-3.567287000	2.410225000	0.164921000
Ge	-2.491569000	-0.490972000	0.154376000
H	-0.542661000	1.888555000	-0.874333000
H	-2.805719000	-0.884345000	1.620354000
H	-1.501535000	1.432461000	-0.293967000
C	-5.621677000	2.425524000	-0.466965000
H	-6.479084000	1.735673000	-0.485073000
H	-5.401519000	2.662237000	-1.519341000
C	-5.989605000	3.692479000	0.285193000
H	-6.852670000	4.198939000	-0.161776000
H	-6.239100000	3.472416000	1.331557000
H	-5.153794000	4.404569000	0.291023000
H	1.566308000	0.509448000	1.888545000
H	1.030025000	-1.453911000	-1.563029000

3.2c-*cis*·H_2

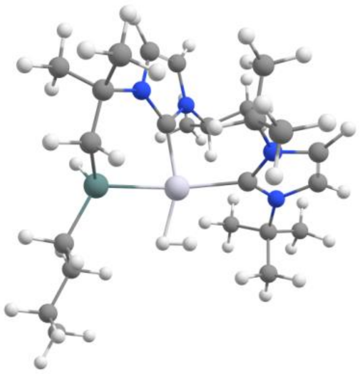

C	3.915085000	0.469810000	-1.482811000
C	3.902540000	1.701830000	-0.929853000
H	4.714168000	-0.080607000	-1.959888000
H	4.701114000	2.421668000	-0.839052000
N	2.638160000	-0.037491000	-1.341499000
N	2.619582000	1.928431000	-0.450320000
C	1.824780000	0.848181000	-0.712274000
Pt	-0.239413000	0.376555000	-0.205206000
C	0.560723000	-3.477502000	1.420199000
C	1.376689000	-2.705122000	2.168323000
H	0.436892000	-4.550039000	1.412039000
H	2.083761000	-2.962276000	2.944872000
C	0.229404000	-1.346615000	0.782268000
N	1.159850000	-1.402371000	1.769764000
N	-0.160456000	-2.639215000	0.581033000
C	-1.228394000	-3.227980000	-0.318041000
C	-0.525951000	-4.083477000	-1.367025000
C	-2.132488000	-4.095866000	0.556588000
C	-2.087971000	-2.164592000	-0.991882000
H	0.132800000	-3.471159000	-1.996419000
H	0.066882000	-4.888418000	-0.914935000
H	-1.278994000	-4.544583000	-2.017163000
H	-2.618219000	-3.497599000	1.336610000
H	-2.912604000	-4.536702000	-0.073582000
H	-1.597244000	-4.921402000	1.035973000
H	-3.007531000	-2.656413000	-1.332495000
H	-1.588034000	-1.773452000	-1.889380000
C	1.786655000	-0.239691000	2.420640000
C	1.280966000	-0.102333000	3.843997000
C	3.297756000	-0.313515000	2.329910000
H	0.186068000	-0.040838000	3.865583000
H	1.595365000	-0.948328000	4.468547000
H	1.686279000	0.812838000	4.291488000
H	3.632675000	-0.429468000	1.291471000
H	3.730445000	0.614078000	2.724928000
H	3.697535000	-1.146417000	2.921880000
C	2.233980000	-1.350138000	-1.881761000
C	2.332965000	-1.345272000	-3.395875000
C	3.041037000	-2.466704000	-1.245416000
H	3.373146000	-1.250922000	-3.732044000
H	1.940036000	-2.288091000	-3.793950000
H	1.752143000	-0.520731000	-3.825570000
H	2.673688000	-3.435726000	-1.603316000
H	4.103264000	-2.396545000	-1.511837000

H	2.954800000	-2.455651000	-0.151550000
C	2.194095000	3.271898000	0.079806000
C	1.528196000	4.036448000	-1.058410000
C	1.260787000	3.131051000	1.274977000
C	3.425977000	4.039413000	0.549671000
H	2.205046000	4.109190000	-1.918642000
H	0.600455000	3.555657000	-1.388661000
H	1.283628000	5.052978000	-0.728274000
H	1.781728000	2.679732000	2.129416000
H	0.935085000	4.133596000	1.575838000
H	0.364883000	2.539172000	1.054198000
H	3.086991000	4.950376000	1.054315000
H	4.015342000	3.456407000	1.268340000
H	4.072964000	4.353206000	-0.276788000
C	-3.983957000	0.448345000	-0.676092000
H	-4.872291000	-0.199664000	-0.689677000
H	-3.730582000	0.668463000	-1.724100000
C	-4.271996000	1.732527000	0.087608000
H	-4.496749000	1.497397000	1.141721000
H	-3.365548000	2.363051000	0.111784000
Ge	-2.490562000	-0.566888000	0.097002000
H	-0.538047000	1.836649000	-1.141560000
H	-2.899710000	-0.996031000	1.536484000
H	-1.283699000	1.671276000	-0.761895000
C	-5.421576000	2.544517000	-0.495369000
H	-6.328425000	1.920870000	-0.514068000
H	-5.196324000	2.781849000	-1.546676000
C	-5.686082000	3.822346000	0.281452000
H	-6.515150000	4.398289000	-0.145231000
H	-5.937884000	3.603107000	1.327433000
H	-4.800254000	4.471522000	0.287955000
H	1.432820000	0.614418000	1.838976000
H	1.180226000	-1.456413000	-1.597745000

3.2c

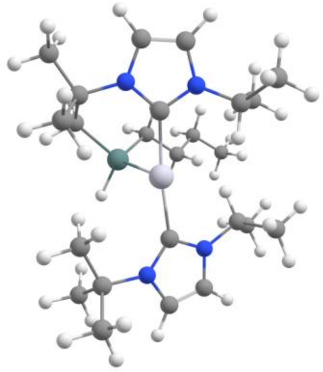

C	1.839415000	-0.579633000	-0.833645000
C	3.981875000	-0.030790000	-1.270912000
H	4.797301000	0.565504000	-1.656709000
C	3.988313000	-1.223368000	-0.628198000
H	4.819382000	-1.856697000	-0.358034000
C	2.198282000	-2.792435000	0.340851000
C	1.265134000	-3.550059000	-0.595971000
H	0.381743000	-2.954739000	-0.850352000
H	0.934672000	-4.476966000	-0.112077000

H	1.788314000	-3.811068000	-1.524139000
C	1.509203000	-2.412208000	1.657243000
H	2.275956000	-2.221372000	2.423308000
H	0.917858000	-3.268748000	2.003155000
C	2.201926000	1.615049000	-1.993304000
H	1.107869000	1.513670000	-2.044739000
C	2.551786000	2.785209000	-1.092019000
H	2.145084000	2.649242000	-0.081490000
H	2.136989000	3.711954000	-1.506829000
H	3.639489000	2.908901000	-1.012958000
C	2.761489000	1.773971000	-3.393271000
H	3.851304000	1.898789000	-3.375517000
H	2.333499000	2.668826000	-3.858648000
H	2.520161000	0.908758000	-4.020506000
C	-2.152187000	0.248732000	-0.357039000
C	-3.845499000	1.748099000	-0.252620000
H	-4.316336000	2.720980000	-0.277775000
C	-4.374342000	0.518530000	-0.064853000
H	-5.399148000	0.226208000	0.113092000
C	-3.621603000	-1.860350000	0.015102000
C	-2.375756000	-2.704750000	-0.172355000
H	-1.910699000	-2.536064000	-1.150897000
H	-2.673293000	-3.758706000	-0.113096000
H	-1.630255000	-2.520828000	0.608213000
C	-4.637907000	-2.251553000	-1.054327000
H	-5.589533000	-1.720875000	-0.946728000
H	-4.847803000	-3.324341000	-0.974610000
H	-4.239118000	-2.053901000	-2.056915000
C	-4.182952000	-2.087928000	1.414714000
H	-3.464929000	-1.761218000	2.177110000
H	-4.376789000	-3.157040000	1.559961000
H	-5.126187000	-1.553168000	1.575357000
C	-1.553659000	2.668937000	-0.731840000
H	-0.557726000	2.236852000	-0.553797000
C	-1.766200000	3.840406000	0.205707000
H	-2.713311000	4.357149000	0.005116000
H	-0.958562000	4.568565000	0.062646000
H	-1.759263000	3.518980000	1.253975000
C	-1.670495000	3.063222000	-2.192736000
H	-1.487652000	2.205783000	-2.852131000
H	-0.936785000	3.843531000	-2.428706000
H	-2.669713000	3.460884000	-2.412817000
C	3.396561000	-3.677992000	0.652018000
H	3.913716000	-4.005695000	-0.258302000
H	3.032657000	-4.572546000	1.169105000
H	4.114268000	-3.183110000	1.316708000
N	-3.335114000	-0.395618000	-0.135301000
N	-2.490610000	1.570188000	-0.423679000
N	2.659823000	0.347306000	-1.391719000
N	2.669884000	-1.553051000	-0.364695000
Pt	-0.171517000	-0.301017000	-0.610191000
Ge	0.393060000	-0.785411000	1.681165000
C	1.466349000	0.669442000	2.435540000
H	1.725160000	0.366797000	3.462128000
H	2.408046000	0.684078000	1.863494000

C	0.807585000	2.038506000	2.426980000
H	-0.153337000	2.001491000	2.968718000
H	0.551546000	2.316872000	1.390286000
C	1.688039000	3.126610000	3.027744000
H	1.890236000	2.887154000	4.082744000
H	2.667142000	3.110139000	2.523010000
C	1.074696000	4.510789000	2.912059000
H	0.101271000	4.558811000	3.418152000
H	1.715916000	5.281273000	3.355057000
H	0.911945000	4.780778000	1.858880000
H	-0.898878000	-1.002764000	2.513686000

TS9

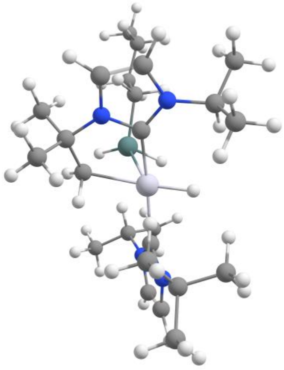

C	1.029825000	-1.462506000	0.306809000
C	2.566956000	-3.074807000	0.545607000
H	3.166082000	-3.928031000	0.259653000
C	2.535138000	-2.370574000	1.705074000
H	3.100155000	-2.491672000	2.619027000
C	1.181133000	-0.353696000	2.539663000
C	0.424279000	-1.082609000	3.646069000
H	-0.442864000	-1.614383000	3.233625000
H	0.067871000	-0.361413000	4.390544000
H	1.070242000	-1.808784000	4.155370000
C	0.262106000	0.664821000	1.844530000
H	0.494500000	1.682176000	2.175476000
H	-0.783510000	0.514587000	2.160274000
C	1.324364000	-2.978690000	-1.666650000
H	0.847637000	-2.121492000	-2.158392000
C	2.590948000	-3.352214000	-2.410965000
H	3.322701000	-2.536652000	-2.399152000
H	2.343147000	-3.576143000	-3.453709000
H	3.060082000	-4.248250000	-1.985843000
C	0.340162000	-4.131005000	-1.584908000
H	0.784689000	-4.979370000	-1.048318000
H	0.072049000	-4.467837000	-2.592449000
H	-0.578427000	-3.833427000	-1.064062000
C	-2.410526000	0.733070000	-0.259653000
C	-4.027949000	2.301161000	-0.461466000
H	-4.435791000	3.298572000	-0.548350000
C	-4.633516000	1.096338000	-0.430004000
H	-5.683575000	0.858975000	-0.484085000
C	-3.976361000	-1.333116000	-0.194589000
C	-3.147241000	-1.993943000	0.901153000
H	-2.077981000	-2.029436000	0.663889000

H	-3.491079000	-3.027644000	1.024991000
H	-3.277888000	-1.472347000	1.858479000
C	-3.760743000	-1.985031000	-1.554619000
H	-4.389117000	-1.500959000	-2.312533000
H	-4.044679000	-3.043012000	-1.500225000
H	-2.717048000	-1.925569000	-1.878871000
C	-5.445183000	-1.487944000	0.193332000
H	-5.680126000	-0.959229000	1.125012000
H	-5.640892000	-2.553496000	0.353091000
H	-6.128806000	-1.151711000	-0.593694000
C	-1.694725000	3.154884000	-0.261907000
H	-0.759979000	2.659052000	-0.003001000
C	-2.038089000	4.111874000	0.863222000
H	-2.952096000	4.680522000	0.652669000
H	-1.220716000	4.831988000	0.985207000
H	-2.169278000	3.576598000	1.811316000
C	-1.545463000	3.841501000	-1.603545000
H	-1.311601000	3.119125000	-2.394468000
H	-0.733987000	4.577242000	-1.554941000
H	-2.466186000	4.371593000	-1.878892000
C	2.420091000	0.323421000	3.112944000
H	3.108302000	-0.399650000	3.565461000
H	2.107786000	1.021613000	3.897681000
H	2.967993000	0.893396000	2.352927000
N	-3.643984000	0.135259000	-0.290389000
N	-2.675424000	2.063394000	-0.339724000
N	1.641692000	-2.497475000	-0.306763000
N	1.592817000	-1.376236000	1.536377000
Pt	-0.534325000	-0.234715000	-0.092962000
Ge	1.342272000	1.500730000	-0.180378000
H	-1.016868000	-1.116704000	-1.327833000
C	3.115155000	0.723168000	-0.470462000
H	3.427625000	0.058113000	0.341969000
H	3.016688000	0.110433000	-1.378513000
C	4.130083000	1.841068000	-0.676121000
H	4.232512000	2.421504000	0.255596000
H	3.766697000	2.554745000	-1.435137000
C	5.497806000	1.321059000	-1.099859000
H	5.853464000	0.596794000	-0.351173000
H	5.391230000	0.758705000	-2.039757000
C	6.515279000	2.435345000	-1.270861000
H	6.661989000	2.983225000	-0.330955000
H	7.491858000	2.051964000	-1.587456000
H	6.182834000	3.160780000	-2.024743000
H	0.777606000	1.876414000	-1.579106000
H	1.484567000	2.849473000	0.573254000

Int A-bis

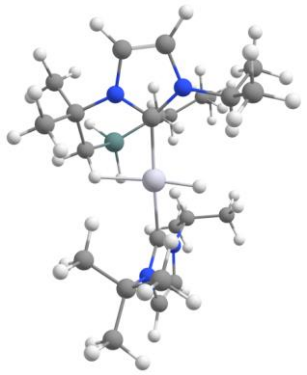

C	-0.979944000	-1.640679000	0.463381000
C	-2.749219000	-2.859464000	1.134829000
H	-3.465371000	-3.269479000	1.833224000
C	-2.618716000	-3.041065000	-0.199652000
H	-3.203192000	-3.640591000	-0.882002000
C	-1.036567000	-2.178518000	-2.000529000
C	-0.030690000	-3.303291000	-2.221264000
H	0.826199000	-3.204536000	-1.543776000
H	0.337149000	-3.281421000	-3.253868000
H	-0.501021000	-4.278530000	-2.044212000
C	-0.380876000	-0.813884000	-2.226477000
H	-0.040233000	-0.791266000	-3.272335000
H	0.615534000	-0.741461000	-1.717529000
C	-1.590097000	-1.486929000	2.903001000
H	-0.892287000	-0.645952000	2.816298000
C	-2.916256000	-0.968860000	3.427142000
H	-3.360327000	-0.245233000	2.732489000
H	-2.757382000	-0.470743000	4.389544000
H	-3.635730000	-1.780086000	3.591576000
C	-0.985645000	-2.562705000	3.784312000
H	-1.645435000	-3.438525000	3.839485000
H	-0.846524000	-2.179763000	4.801308000
H	-0.011017000	-2.886270000	3.399358000
C	2.232314000	0.913809000	0.157061000
C	3.337822000	2.876242000	0.223781000
H	3.461237000	3.948034000	0.283762000
C	4.258359000	1.893112000	0.101477000
H	5.332132000	1.963539000	0.030265000
C	4.231084000	-0.656909000	-0.030654000
C	3.610917000	-1.459761000	-1.167749000
H	2.566598000	-1.723590000	-0.958795000
H	4.171347000	-2.393619000	-1.293717000
H	3.653789000	-0.901804000	-2.111887000
C	4.058409000	-1.368048000	1.305645000
H	4.502631000	-0.778921000	2.117647000
H	4.563616000	-2.340742000	1.271033000
H	2.998696000	-1.536816000	1.532596000
C	5.714519000	-0.470588000	-0.318482000
H	5.882856000	0.072932000	-1.256037000
H	6.169366000	-1.461507000	-0.419189000
H	6.234677000	0.046915000	0.495604000
C	0.822187000	2.978268000	0.422503000
H	0.058179000	2.270951000	0.074609000
C	0.780400000	4.219334000	-0.447260000

H	1.474931000	4.990193000	-0.090230000
H	-0.226589000	4.649794000	-0.415343000
H	1.021155000	3.985845000	-1.491126000
C	0.585662000	3.286631000	1.888467000
H	0.585576000	2.370432000	2.491351000
H	-0.386036000	3.780210000	2.011307000
H	1.360406000	3.960283000	2.277362000
C	-2.209165000	-2.334452000	-2.963305000
H	-2.592511000	-3.360364000	-2.970767000
H	-1.868511000	-2.106286000	-3.979219000
H	-3.037164000	-1.657558000	-2.718608000
N	3.572126000	0.689641000	0.054958000
N	2.102639000	2.261656000	0.247801000
N	-1.746826000	-1.988591000	1.523443000
N	-1.540305000	-2.272903000	-0.603587000
Pt	0.637001000	-0.399740000	0.294822000
Ge	-1.489811000	0.822872000	-2.071404000
H	0.843521000	-0.294475000	1.816433000
C	-2.635737000	1.026182000	-0.494046000
H	-3.333594000	0.175536000	-0.455670000
H	-2.008704000	0.968131000	0.408279000
C	-3.393793000	2.346975000	-0.539517000
H	-4.004947000	2.399729000	-1.455576000
H	-2.677785000	3.184176000	-0.609116000
C	-4.289175000	2.560729000	0.673506000
H	-4.981806000	1.709387000	0.762313000
H	-3.667650000	2.544548000	1.582688000
C	-5.069750000	3.861480000	0.602967000
H	-5.715385000	3.885824000	-0.284583000
H	-5.708508000	4.004064000	1.481897000
H	-4.394056000	4.724626000	0.540232000
H	-0.511415000	2.017501000	-2.168781000
H	-2.388231000	0.870406000	-3.332621000

Int A'

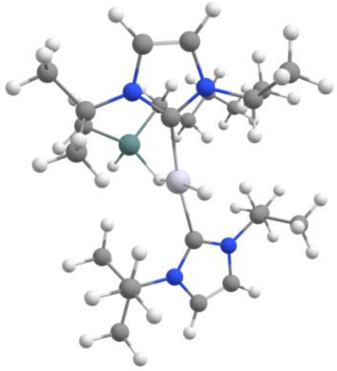

C	-1.473182000	-0.799302000	1.055188000
C	-3.549079000	-0.645429000	1.912023000
H	-4.373036000	-0.199311000	2.450418000
C	-3.464309000	-1.830156000	1.264582000
H	-4.205301000	-2.605688000	1.151726000
C	-1.650282000	-3.057491000	-0.070110000
C	-0.361593000	-3.562185000	0.569688000
H	0.426439000	-2.801366000	0.563364000
H	-0.004217000	-4.439199000	0.017290000

H	-0.545810000	-3.857626000	1.609813000
C	-1.432298000	-2.607317000	-1.520909000
H	-2.375278000	-2.722698000	-2.077098000
H	-0.708437000	-3.277415000	-2.000220000
C	-2.000072000	1.302252000	2.339738000
H	-0.904054000	1.350704000	2.334514000
C	-2.557021000	2.390787000	1.440449000
H	-2.139684000	2.321848000	0.427988000
H	-2.308140000	3.377686000	1.848448000
H	-3.650836000	2.317394000	1.370822000
C	-2.497276000	1.421346000	3.767338000
H	-3.592730000	1.450949000	3.818085000
H	-2.125272000	2.356586000	4.199212000
H	-2.140573000	0.591088000	4.386797000
C	2.317418000	0.559912000	0.146855000
C	3.893035000	2.162779000	-0.009744000
H	4.291362000	3.167701000	0.005524000
C	4.493287000	0.974921000	-0.248245000
H	5.524234000	0.758468000	-0.481103000
C	3.769680000	-1.478376000	-0.315218000
C	2.859438000	-2.028338000	-1.406871000
H	1.802592000	-1.953978000	-1.129260000
H	3.092879000	-3.087175000	-1.571284000
H	3.012525000	-1.490632000	-2.351419000
C	3.519387000	-2.163874000	1.021836000
H	4.200809000	-1.772566000	1.787510000
H	3.697386000	-3.241385000	0.920367000
H	2.488810000	-2.013150000	1.363415000
C	5.217338000	-1.698845000	-0.729715000
H	5.451104000	-1.209074000	-1.682733000
H	5.373727000	-2.774748000	-0.861476000
H	5.923735000	-1.354880000	0.034595000
C	1.549245000	2.915136000	0.549439000
H	0.599715000	2.363132000	0.570749000
C	1.488969000	3.966457000	-0.541016000
H	2.414017000	4.555381000	-0.584936000
H	0.664612000	4.659125000	-0.335820000
H	1.320194000	3.509028000	-1.523643000
C	1.815446000	3.497450000	1.923993000
H	1.855753000	2.707846000	2.684291000
H	1.015167000	4.196777000	2.192824000
H	2.765033000	4.047531000	1.945272000
C	-2.658276000	-4.200000000	-0.062981000
H	-2.839265000	-4.576617000	0.951166000
H	-2.241904000	-5.021250000	-0.656335000
H	-3.613042000	-3.918309000	-0.521390000
N	3.516876000	-0.007330000	-0.152746000
N	2.560128000	1.890185000	0.226688000
N	-2.318377000	-0.030300000	1.785045000
N	-2.182691000	-1.919652000	0.745170000
Pt	0.422937000	-0.216999000	0.481698000
Ge	-0.949831000	-0.763695000	-1.973736000
H	0.805311000	-0.238451000	2.018250000
C	-2.468641000	0.455787000	-2.001834000
H	-3.171523000	0.066710000	-2.752713000

H	-2.983132000	0.410253000	-1.030661000
C	-2.060057000	1.883463000	-2.335671000
H	-1.522064000	1.907730000	-3.297898000
H	-1.335930000	2.248048000	-1.585298000
C	-3.240713000	2.843159000	-2.398350000
H	-3.916797000	2.526127000	-3.206509000
H	-3.822456000	2.759141000	-1.467017000
C	-2.807637000	4.282955000	-2.608226000
H	-2.214494000	4.389024000	-3.526030000
H	-3.663794000	4.962416000	-2.685703000
H	-2.184900000	4.629805000	-1.772002000
H	0.295191000	0.048260000	-1.278837000
H	-0.280813000	-0.799930000	-3.368472000

TS10

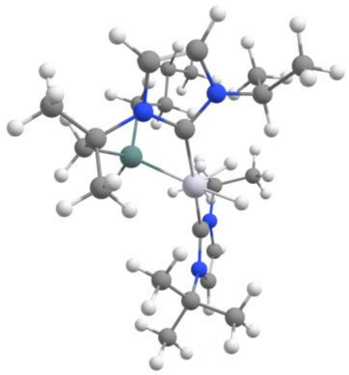

C	-1.484422000	-1.248855000	0.708032000
C	-3.594560000	-1.493885000	1.457080000
H	-4.440397000	-1.344825000	2.112337000
C	-3.474978000	-2.231098000	0.331712000
H	-4.204007000	-2.855918000	-0.158717000
C	-1.650204000	-2.747257000	-1.372981000
C	-0.460694000	-3.604801000	-0.969640000
H	0.364739000	-3.007087000	-0.577636000
H	-0.091841000	-4.154482000	-1.843331000
H	-0.752929000	-4.329412000	-0.200086000
C	-1.288949000	-1.701150000	-2.437890000
H	-2.189625000	-1.459402000	-3.020654000
H	-0.573161000	-2.154610000	-3.134862000
C	-2.133239000	0.025167000	2.807121000
H	-1.048176000	0.034683000	2.957699000
C	-2.626102000	1.412129000	2.432791000
H	-2.134556000	1.786945000	1.524695000
H	-2.427677000	2.116500000	3.248540000
H	-3.708906000	1.397213000	2.250032000
C	-2.784244000	-0.489753000	4.076770000
H	-3.878502000	-0.443371000	4.025923000
H	-2.470441000	0.138862000	4.916638000
H	-2.486326000	-1.522088000	4.290248000
C	2.224089000	0.570729000	0.119551000
C	3.703946000	2.259931000	-0.066191000
H	4.036522000	3.285252000	-0.141065000
C	4.403694000	1.105882000	-0.059032000
H	5.467715000	0.939952000	-0.129293000
C	3.915383000	-1.360413000	0.151113000

C	2.791843000	-2.269480000	-0.313182000
H	1.958355000	-2.264696000	0.407022000
H	3.150410000	-3.304979000	-0.344562000
H	2.439309000	-2.000746000	-1.317578000
C	4.270877000	-1.638268000	1.606113000
H	5.087492000	-0.986316000	1.939402000
H	4.597621000	-2.679070000	1.716575000
H	3.405556000	-1.475719000	2.260503000
C	5.124203000	-1.587958000	-0.749955000
H	4.909792000	-1.291954000	-1.783895000
H	5.366302000	-2.656278000	-0.742829000
H	6.016375000	-1.055362000	-0.404530000
C	1.275262000	2.902255000	0.079372000
H	0.380850000	2.329736000	-0.189035000
C	1.472667000	3.995533000	-0.951228000
H	2.315606000	4.650457000	-0.698107000
H	0.571534000	4.619843000	-0.980931000
H	1.636235000	3.579345000	-1.952270000
C	1.119193000	3.453231000	1.484749000
H	0.968865000	2.648476000	2.215809000
H	0.253058000	4.125390000	1.527065000
H	2.008753000	4.024853000	1.780084000
C	-2.719377000	-3.672861000	-1.939179000
H	-2.994466000	-4.470410000	-1.237968000
H	-2.304924000	-4.143931000	-2.837090000
H	-3.620664000	-3.128754000	-2.242972000
N	3.487222000	0.069255000	0.042001000
N	2.369453000	1.916323000	0.030812000
N	-2.366563000	-0.907132000	1.684075000
N	-2.179120000	-2.077863000	-0.127688000
Pt	0.419952000	-0.417382000	0.475492000
Ge	-0.593373000	0.041051000	-1.790940000
H	0.940454000	-0.795902000	2.005128000
C	-2.218436000	1.172619000	-1.722776000
H	-2.682523000	1.109518000	-2.719305000
H	-2.919297000	0.686452000	-1.023332000
C	-2.009753000	2.625996000	-1.336890000
H	-1.277541000	3.093460000	-2.018022000
H	-1.559127000	2.685664000	-0.330850000
C	-3.286506000	3.454363000	-1.327756000
H	-3.750776000	3.420559000	-2.325010000
H	-4.010122000	2.987790000	-0.640122000
C	-3.031032000	4.893697000	-0.915870000
H	-2.340988000	5.386403000	-1.614084000
H	-3.951866000	5.487277000	-0.887675000
H	-2.575233000	4.940400000	0.083794000
H	0.021967000	0.704325000	1.413273000
H	0.319332000	0.629540000	-2.912699000

3.2c-H_2

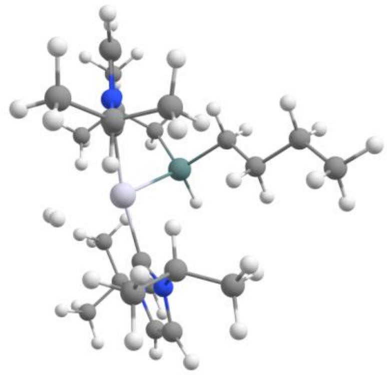

C	1.822768000	-0.663323000	-0.815681000
C	3.989528000	-0.175038000	-1.199484000
H	4.829374000	0.400055000	-1.564541000
C	3.946604000	-1.363247000	-0.551267000
H	4.752332000	-2.016739000	-0.254234000
C	2.088214000	-2.858898000	0.410333000
C	1.139820000	-3.607464000	-0.519228000
H	0.275092000	-2.994976000	-0.797250000
H	0.777424000	-4.513024000	-0.018022000
H	1.663481000	-3.905453000	-1.436056000
C	1.399478000	-2.417626000	1.708836000
H	2.165629000	-2.242451000	2.479060000
H	0.771680000	-3.242841000	2.067081000
C	2.280589000	1.523350000	-1.959594000
H	1.183961000	1.484003000	-1.983521000
C	2.704428000	2.682862000	-1.077015000
H	2.306270000	2.571058000	-0.059780000
H	2.322443000	3.623567000	-1.492399000
H	3.797420000	2.760666000	-1.015795000
C	2.811568000	1.630519000	-3.374890000
H	3.907781000	1.675205000	-3.388748000
H	2.435431000	2.548105000	-3.840860000
H	2.491238000	0.776257000	-3.983094000
C	-2.143837000	0.316047000	-0.314792000
C	-3.763356000	1.890537000	-0.160495000
H	-4.188982000	2.884240000	-0.171339000
C	-4.341168000	0.686909000	0.042390000
H	-5.371900000	0.440459000	0.251040000
C	-3.694322000	-1.726292000	0.078536000
C	-2.486693000	-2.622514000	-0.121475000
H	-2.011847000	-2.459782000	-1.096726000
H	-2.831776000	-3.662805000	-0.081047000
H	-1.736538000	-2.488808000	0.665004000
C	-4.729623000	-2.058611000	-0.992254000
H	-5.653467000	-1.481929000	-0.878607000
H	-4.992634000	-3.120339000	-0.922672000
H	-4.322680000	-1.871600000	-1.993877000
C	-4.258962000	-1.947904000	1.477567000
H	-3.523034000	-1.664346000	2.240238000
H	-4.497834000	-3.009749000	1.608423000
H	-5.177835000	-1.376441000	1.651922000
C	-1.443129000	2.704115000	-0.707892000
H	-0.462978000	2.234077000	-0.536015000
C	-1.589561000	3.901063000	0.210259000

H	-2.512380000	4.459330000	0.008541000
H	-0.750075000	4.586674000	0.042850000
H	-1.586568000	3.601552000	1.264730000
C	-1.564305000	3.083605000	-2.172629000
H	-1.445858000	2.208556000	-2.823503000
H	-0.790336000	3.815530000	-2.434120000
H	-2.542704000	3.536201000	-2.379698000
C	3.250683000	-3.779696000	0.754369000
H	3.767695000	-4.144557000	-0.141860000
H	2.848629000	-4.649774000	1.284799000
H	3.976834000	-3.297025000	1.418815000
N	-3.344643000	-0.274107000	-0.057046000
N	-2.420778000	1.651148000	-0.369302000
N	2.681227000	0.239773000	-1.353426000
N	2.612143000	-1.654765000	-0.319744000
Pt	-0.192056000	-0.326183000	-0.640616000
Ge	0.346109000	-0.741258000	1.672539000
H	-0.605651000	-0.599838000	-2.769616000
C	1.499300000	0.657399000	2.425602000
H	1.742328000	0.356488000	3.456172000
H	2.441999000	0.615108000	1.855699000
C	0.918775000	2.060984000	2.397728000
H	-0.036974000	2.086759000	2.949335000
H	0.666152000	2.334082000	1.358541000
C	1.862431000	3.112105000	2.967696000
H	2.066687000	2.879906000	4.023963000
H	2.831970000	3.035822000	2.450070000
C	1.321196000	4.524770000	2.836241000
H	0.357294000	4.632313000	3.351466000
H	2.007123000	5.267623000	3.259187000
H	1.160442000	4.786688000	1.780621000
H	-0.489925000	0.148948000	-2.867255000
H	-0.928034000	-0.900482000	2.549357000

TS11

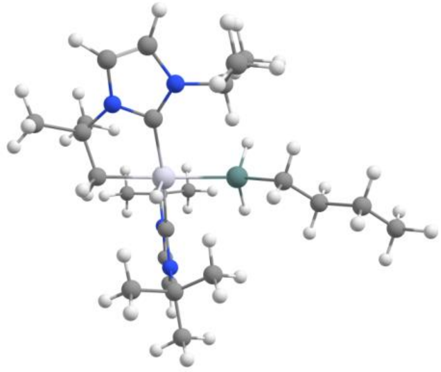

C	1.632639000	1.681485000	-0.285550000
C	2.556187000	3.723590000	-0.309329000
H	2.573432000	4.804258000	-0.276589000
C	3.561852000	2.822499000	-0.451080000
H	4.627919000	2.967423000	-0.557506000
C	3.619376000	0.242493000	-0.531593000
C	4.777905000	0.300294000	-1.516177000
H	4.447102000	0.674040000	-2.492670000
H	5.176130000	-0.711729000	-1.653347000

H	5.599849000	0.931467000	-1.155366000
C	2.529198000	-0.722964000	-1.006517000
H	2.764037000	-1.747498000	-0.687380000
H	2.487665000	-0.720405000	-2.104072000
C	0.046209000	3.615939000	-0.070447000
H	-0.643539000	2.771715000	-0.170326000
C	-0.099629000	4.241387000	1.303414000
H	0.104080000	3.512204000	2.096297000
H	-1.120757000	4.617848000	1.434173000
H	0.588704000	5.088111000	1.422663000
C	-0.215786000	4.599716000	-1.193666000
H	0.447589000	5.471142000	-1.128725000
H	-1.247736000	4.961946000	-1.124200000
H	-0.080233000	4.130995000	-2.174776000
C	-0.092708000	-2.044472000	0.040169000
C	-0.380424000	-3.981644000	1.151139000
H	-0.274561000	-4.687434000	1.963089000
C	-1.022859000	-4.085923000	-0.032075000
H	-1.583442000	-4.913220000	-0.436398000
C	-1.381740000	-2.624724000	-2.094098000
C	-0.211369000	-2.506686000	-3.062594000
H	0.425546000	-1.644434000	-2.838327000
H	-0.593834000	-2.386868000	-4.082989000
H	0.408544000	-3.411292000	-3.031473000
C	-2.234599000	-1.364211000	-2.076343000
H	-3.064694000	-1.471205000	-1.366465000
H	-2.655994000	-1.203819000	-3.075586000
H	-1.658444000	-0.473388000	-1.805389000
C	-2.260279000	-3.792411000	-2.523556000
H	-1.697399000	-4.729036000	-2.608410000
H	-2.665839000	-3.562184000	-3.514312000
H	-3.106964000	-3.939642000	-1.843104000
C	0.956406000	-2.184612000	2.315462000
H	1.210052000	-1.145194000	2.036591000
C	2.242166000	-2.964742000	2.504529000
H	2.028667000	-4.002074000	2.791609000
H	2.837836000	-2.512088000	3.304957000
H	2.842338000	-2.976518000	1.587357000
C	0.096392000	-2.148380000	3.564333000
H	-0.833778000	-1.594202000	3.389686000
H	0.645573000	-1.662002000	4.377536000
H	-0.158731000	-3.163285000	3.893286000
C	4.124447000	-0.118953000	0.860131000
H	4.826672000	0.638322000	1.231200000
H	4.649767000	-1.081125000	0.827284000
H	3.295777000	-0.201425000	1.576203000
N	-0.844036000	-2.887918000	-0.714570000
N	0.189405000	-2.724163000	1.177461000
N	1.378106000	3.006017000	-0.215836000
N	2.972024000	1.578775000	-0.455017000
Pt	0.643793000	-0.120630000	-0.157138000
Ge	-1.534677000	0.547830000	1.026114000
H	0.383241000	-0.032584000	-1.642606000
C	-2.936196000	1.586065000	0.066721000
H	-2.740372000	2.655317000	0.237451000

H	-2.830997000	1.414334000	-1.015158000
C	-4.349417000	1.252383000	0.524507000
H	-4.431948000	1.395985000	1.615150000
H	-4.559158000	0.184270000	0.351450000
C	-5.414246000	2.090302000	-0.172454000
H	-5.211980000	3.156259000	0.014718000
H	-5.322197000	1.952374000	-1.261067000
C	-6.823016000	1.743368000	0.276840000
H	-6.942436000	1.898641000	1.357175000
H	-7.578261000	2.354369000	-0.230892000
H	-7.055235000	0.690212000	0.070584000
H	-2.299185000	-0.727684000	1.523712000
H	-1.310543000	1.339891000	2.361198000

Int B

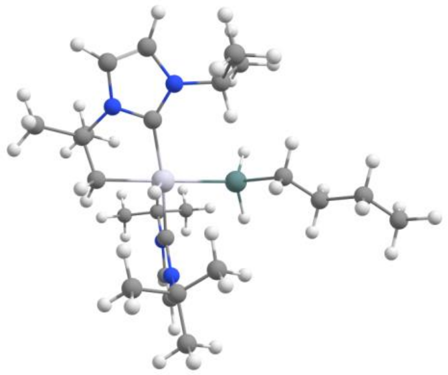

C	1.632951000	1.686214000	-0.270597000
C	2.537672000	3.736195000	-0.326359000
H	2.544824000	4.817352000	-0.311505000
C	3.551913000	2.842441000	-0.451361000
H	4.616858000	2.995688000	-0.557245000
C	3.632099000	0.261993000	-0.477627000
C	4.806307000	0.309538000	-1.443727000
H	4.489530000	0.661892000	-2.432762000
H	5.214003000	-0.701901000	-1.554963000
H	5.617679000	0.953461000	-1.081619000
C	2.558978000	-0.723059000	-0.954029000
H	2.790230000	-1.737401000	-0.601429000
H	2.545399000	-0.750783000	-2.051805000
C	0.028596000	3.608836000	-0.087541000
H	-0.652879000	2.756651000	-0.174202000
C	-0.122698000	4.252721000	1.277185000
H	0.080806000	3.534350000	2.079988000
H	-1.144937000	4.628751000	1.400357000
H	0.563088000	5.102765000	1.386987000
C	-0.243313000	4.572854000	-1.225209000
H	0.409259000	5.453246000	-1.172465000
H	-1.279800000	4.923268000	-1.162464000
H	-0.100897000	4.091585000	-2.199286000
C	-0.093446000	-2.047690000	0.014786000
C	-0.425042000	-3.998528000	1.089173000
H	-0.336620000	-4.720299000	1.889075000
C	-1.061328000	-4.070188000	-0.099847000
H	-1.634619000	-4.880206000	-0.521186000
C	-1.374118000	-2.569291000	-2.140661000

C	-0.192809000	-2.459984000	-3.097116000
H	0.460629000	-1.614982000	-2.855290000
H	-0.563746000	-2.317199000	-4.118844000
H	0.407546000	-3.377870000	-3.074333000
C	-2.206728000	-1.295105000	-2.111692000
H	-3.046659000	-1.401869000	-1.413266000
H	-2.614774000	-1.112916000	-3.112777000
H	-1.621742000	-0.416364000	-1.820186000
C	-2.269460000	-3.714018000	-2.597046000
H	-1.723043000	-4.659237000	-2.693560000
H	-2.662599000	-3.459770000	-3.586935000
H	-3.124249000	-3.857165000	-1.926017000
C	0.943808000	-2.251943000	2.289729000
H	1.233854000	-1.220252000	2.020147000
C	2.200168000	-3.080104000	2.477681000
H	1.949055000	-4.104148000	2.781175000
H	2.822417000	-2.641283000	3.265374000
H	2.788109000	-3.131823000	1.553987000
C	0.079702000	-2.200769000	3.535370000
H	-0.830431000	-1.613453000	3.364329000
H	0.641922000	-1.742399000	4.356008000
H	-0.210903000	-3.210019000	3.852224000
C	4.116101000	-0.064286000	0.930311000
H	4.811096000	0.704283000	1.291604000
H	4.642527000	-1.026075000	0.932658000
H	3.275154000	-0.124607000	1.634681000
N	-0.854784000	-2.864288000	-0.760328000
N	0.168007000	-2.752836000	1.140922000
N	1.365999000	3.009095000	-0.223178000
N	2.973184000	1.593386000	-0.436092000
Pt	0.655006000	-0.122417000	-0.150105000
Ge	-1.526741000	0.530027000	1.040115000
H	0.460301000	-0.036653000	-1.646882000
C	-2.930757000	1.586511000	0.104027000
H	-2.735606000	2.652071000	0.296192000
H	-2.827449000	1.436102000	-0.981242000
C	-4.342570000	1.242423000	0.558414000
H	-4.423236000	1.367465000	1.651468000
H	-4.551188000	0.177145000	0.367605000
C	-5.409781000	2.090464000	-0.122488000
H	-5.207039000	3.153595000	0.079619000
H	-5.321322000	1.968516000	-1.213304000
C	-6.816981000	1.736659000	0.326403000
H	-6.933090000	1.877381000	1.409081000
H	-7.574085000	2.354051000	-0.170734000
H	-7.049328000	0.686217000	0.106842000
H	-2.291293000	-0.754596000	1.513612000
H	-1.299950000	1.296296000	2.389870000

TS12

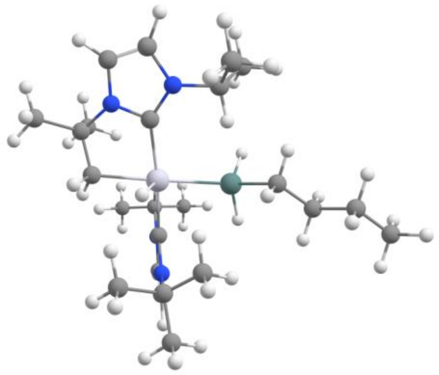

C	1.638844000	1.681566000	-0.270733000
C	2.550378000	3.729066000	-0.327007000
H	2.560261000	4.810262000	-0.323051000
C	3.564752000	2.832156000	-0.422195000
H	4.631705000	2.982389000	-0.509675000
C	3.637928000	0.251867000	-0.419086000
C	4.841678000	0.288441000	-1.348545000
H	4.559276000	0.639392000	-2.348342000
H	5.247026000	-0.725611000	-1.443070000
H	5.644240000	0.928947000	-0.961591000
C	2.578105000	-0.732685000	-0.926730000
H	2.785323000	-1.746818000	-0.560138000
H	2.604708000	-0.767225000	-2.023715000
C	0.036730000	3.609273000	-0.130889000
H	-0.647267000	2.760350000	-0.233602000
C	-0.136390000	4.250173000	1.232963000
H	0.039640000	3.526022000	2.037135000
H	-1.155218000	4.640930000	1.335935000
H	0.559264000	5.089527000	1.361076000
C	-0.209157000	4.580097000	-1.268812000
H	0.443001000	5.459351000	-1.196252000
H	-1.246409000	4.931120000	-1.225575000
H	-0.046914000	4.104796000	-2.242657000
C	-0.112223000	-2.044253000	-0.000445000
C	-0.475888000	-4.000110000	1.053905000
H	-0.401327000	-4.729993000	1.847770000
C	-1.106367000	-4.052814000	-0.139214000
H	-1.689248000	-4.850781000	-0.570301000
C	-1.381054000	-2.534093000	-2.171811000
C	-0.190691000	-2.468166000	-3.121105000
H	0.496764000	-1.652311000	-2.872401000
H	-0.548343000	-2.306221000	-4.144690000
H	0.371324000	-3.409874000	-3.097678000
C	-2.176787000	-1.235623000	-2.152966000
H	-3.023031000	-1.316563000	-1.458597000
H	-2.576129000	-1.049542000	-3.156841000
H	-1.571604000	-0.368656000	-1.865848000
C	-2.310588000	-3.650220000	-2.632693000
H	-1.794098000	-4.611673000	-2.732853000
H	-2.694599000	-3.380767000	-3.622176000
H	-3.169810000	-3.768843000	-1.962533000
C	0.909955000	-2.285327000	2.278896000
H	1.207500000	-1.253312000	2.023330000
C	2.159314000	-3.126309000	2.457594000

H	1.900886000	-4.154135000	2.741688000
H	2.781302000	-2.705590000	3.255382000
H	2.751758000	-3.165847000	1.536177000
C	0.044008000	-2.247616000	3.523895000
H	-0.862582000	-1.652678000	3.360778000
H	0.608005000	-1.803645000	4.351223000
H	-0.252565000	-3.259202000	3.827431000
C	4.080383000	-0.071113000	1.003522000
H	4.770499000	0.694915000	1.379193000
H	4.600418000	-1.036206000	1.024561000
H	3.221210000	-0.122351000	1.685518000
N	-0.880300000	-2.843847000	-0.787600000
N	0.133289000	-2.762863000	1.120593000
N	1.374771000	3.005255000	-0.241345000
N	2.982409000	1.584535000	-0.406114000
Pt	0.656416000	-0.127449000	-0.160368000
Ge	-1.511111000	0.526207000	1.056143000
H	0.577064000	-0.138046000	-1.673473000
C	-2.910292000	1.602615000	0.137796000
H	-2.708458000	2.664122000	0.343625000
H	-2.810715000	1.464502000	-0.949432000
C	-4.322332000	1.259695000	0.592389000
H	-4.398772000	1.369773000	1.687328000
H	-4.537469000	0.198428000	0.386935000
C	-5.386453000	2.123687000	-0.073126000
H	-5.176482000	3.182635000	0.143108000
H	-5.302130000	2.016464000	-1.165815000
C	-6.794362000	1.772275000	0.375323000
H	-6.906077000	1.898171000	1.460276000
H	-7.549026000	2.401660000	-0.110327000
H	-7.034179000	0.726595000	0.141532000
H	-2.281566000	-0.760062000	1.513793000
H	-1.270206000	1.270963000	2.414745000

17. Crystallographic details

Low-temperature diffraction data were collected on a Bruker D8 Quest APEX-III single crystal diffractometer with a Photon III detector and a IµS 3.0 microfocus X-ray source at the Instituto de Investigaciones Químicas, Sevilla. Data were collected by means of ω and φ scans using monochromatic radiation λ(Mo Kα1) = 0.71073 Å. The diffraction images collected were processed and scaled using APEX-III v2018.7-2 software. The structures were solved with SHELXT and was refined against F2 on all data by full-matrix least squares with SHELXL,[28] using Olex2[29] as graphical interface. All non-hydrogen atoms were refined anisotropically. Hydrogen atoms were included in the model at geometrically calculated positions and refined using a riding model, unless otherwise noted. The isotropic displacement parameters of all hydrogen atoms were fixed to 1.2 times the U value of the atoms to which they are linked (1.5 times for methyl groups). An elevated disorder is observed for the fluorine atoms of the CF_3 terminal groups of the BAr^F anion with some of these atoms splitting into two positions.

The unit cell for complex **2.1a** contains one dichloromethane molecule and two molecules of pentane for complex **2.4d** which were treated as a diffuse contribution to the overall scattering without specific atom positions by SQUEEZE.[30]

Crystal Data for $C_{55}H_{57}BCl_2F_{24}GeN_4Pt$ (**2.1a**) (*M* =1579.43 g/mol): triclinic, space group P-1 (no. 2), *a* = 12.7577(13) Å, *b* = 13.4103(13) Å, *c* = 20.2671(18) Å, *α* = 72.474(4)°, *β* = 82.510(4)°, *γ* = 89.255(4)°, *V* = 3277.0(6) Å^3, *Z* = 2, *T* = 193.0 K, μ(MoKα) = 2.778 mm^{-1}, *Dcalc* = 1.601 g/cm^3, 123830 reflections measured (4.088° ≤ 2Θ ≤ 56.574°), 16223 unique (R_{int} = 0.0709, R_{sigma} = 0.0401) which were used in all calculations. The final R_1 was 0.0502 (I > 2σ(I)) and wR_2 was 0.1362 (all data).

Crystal Data for $C_{66}H_{55}BF_{24}GeN_4Pt$ (**2.1b**) (*M* =1638.63 g/mol): triclinic, space group P-1 (no. 2), *a* = 10.1223(4) Å, *b* = 17.2293(9) Å, *c* = 20.3633(11) Å, *α* = 72.674(2)°, *β* = 80.567(2)°, *γ* = 82.436(2)°, *V* = 3331.4(3) Å^3, *Z* = 2, *T* = 193.0 K, μ(MoKα) = 2.659 mm^{-1}, *Dcalc* = 1.634 g/cm^3, 172585 reflections measured (4.228° ≤ 2Θ ≤ 61.012°), 20309 unique (R_{int} = 0.0616, R_{sigma} = 0.0325) which were used in all calculations. The final R_1 was 0.0332 (I > 2σ(I)) and wR_2 was 0.0816 (all data).

Crystal Data for $C_{70}H_{63}BF_{24}GeN_4Pt$ (**2.2b**) (*M* =1694.73 g/mol): monoclinic, space group P2$_1$/c (no. 14), *a* = 19.0248(15) Å, *b* = 20.3499(16) Å, *c* = 19.2765(14) Å, *β* = 108.554(3)°, *V* = 7075.1(9) Å^3, *Z* = 4, *T* = 193.0 K, μ(MoKα) = 2.507 mm^{-1}, *Dcalc* = 1.591 g/cm^3, 152578 reflections measured (4.002° ≤ 2Θ ≤ 56.61°), 17572 unique (R_{int} = 0.0831, R_{sigma} = 0.0429) which were used in all calculations. The final R_1 was 0.0348 (I > 2σ(I)) and wR_2 was 0.0919 (all data).

Crystal Data for $C_{74}H_{71}BF_{24}GeN_4Pt$ (**2.3b**) (*M* =1750.83 g/mol): triclinic, space group P-1 (no. 2), *a* = 13.2831(11) Å, *b* = 20.1756(18) Å, *c* = 29.543(3) Å, *α* = 77.621(4)°, *β* = 87.514(4)°, *γ* = 82.073(4)°, *V* = 7658.5(12) Å^3, *Z* = 4, *T* = 193.0 K, μ(MoKα) = 2.319 mm^{-1}, *Dcalc* = 1.518 g/cm^3, 236735 reflections measured (3.954° ≤ 2Θ ≤ 56.554°), 37937 unique (R_{int} = 0.0862, R_{sigma} = 0.0566) which were used in all calculations. The final R_1 was 0.0387 (I > 2σ(I)) and wR_2 was 0.0826 (all data).

Crystal Data for $C_{186}H_{176}B_2F_{48}Ge_2N_8OPt_2Si_2$ (**2.4d**) (*M* =4064.50 g/mol): triclinic, space group P-1 (no. 2), *a* = 13.6570(8) Å, *b* = 19.7876(12) Å, *c* = 20.0919(11) Å, *α* = 89.810(2)°, *β* = 71.370(2)°, *γ* = 72.219(2)°, *V* = 4872.5(5) Å^3, *Z* = 1, *T* = 193.0 K, μ(MoKα) = 1.845 mm^{-1}, *Dcalc* = 1.385 g/cm^3, 198214 reflections measured (4.302° ≤ 2Θ ≤ 51.388°), 18513 unique (R_{int} = 0.1320, R_{sigma} = 0.0557) which were used in all calculations. The final R_1 was 0.0423 (I > 2σ(I)) and wR_2 was 0.0937 (all data).

Crystal Data for $C_{58}H_{63}BF_{24}GeN_4Pt$ (**1.2**·$HGeEt_3$) (*M* =1550.61 g/mol): monoclinic, space group $P2_1/c$ (no. 14), *a* = 13.4189(14) Å, *b* = 25.073(2) Å, *c* = 19.563(2) Å, *β* = 102.844(4)°, *V* = 6417.4(11) $Å^3$, *Z* = 4, *T* = 193.00 K, μ(MoKα) = 2.755 mm^{-1}, *Dcalc* = 1.605 g/cm^3, 204997 reflections measured (3.888° ≤ 2Θ ≤ 61.108°), 19594 unique (R_{int} = 0.0574, R_{sigma} = 0.0318) which were used in all calculations. The final R_1 was 0.0354 (I > 2σ(I)) and wR_2 was 0.0880 (all data).

18. References

[1] Cowley, R. E.; Bontchev, R. P.; Duesler, E. N.; Smith. J. M. Removing the Sting from the Tail: Reversible Protonation of Scorpionate Ligands in Cobalt(II) Tris(carbene)borate Complexes. *Inorg. Chem.* **2006**, *45*, 9771–9779.

[2] Brookhart, M.; Grant, B.; Volp, A. F. $[(3,5\text{-}(CF_3)_2C_6H_3)_4B]\text{-}[H(OEt_2)_2]^+$: a convenient reagent for generation and stabilization of cationic, highly electrophilic organometallic complexes. *Organometallics* **1992**, *11*, 3920–3922.

[3] Bassan, R.; Bryars, K. H.; Judd, L.; Platt, A. W. G.; Pringle, P. G. Improved syntheses of $[PtMe_2(1,5\text{-}COD)]$. *Inorg. Chim. Acta* **1986**, *121*, L41–L42.

[4] Ríos, P.; Fouilloux, H.; Vidossich, P.; Díez, J.; Lledós, A.; Conejero; S. Isolation of a Cationic Platinum(II) σ-Silane Complex. *Angew. Chem., Int. Ed.* **2018**, *57*, 3217–3221.

[5] Fouilloux, H.; Rager, M.-N.; Ríos, P.; Conejero, S.; Thomas, C. M. Highly efficient synthesis of poly(silylether)s: access to degradable polymers from renewable resources. *Angew. Chem., Int. Ed.* **2022**, *61*, e202113443.

[6] Zhao, Y; Truhlar; D. G. The M06 suite of density functionals for main group thermochemistry, thermochemical kinetics, noncovalent interactions, excited states, and transition elements: two new functionals and systematic testing of four M06-class functionals and 12 other functionals. *Theor. Chem. Acc.* **2008**, *120*, 215–241.

[7] Gaussian 09, Revision E.01, Frisch, M. J.; Trucks, G. W.; Schlegel, H. B.; Scuseria, G. E.; Robb, M. A.; Cheeseman, J. R.; Scalmani, G.; Barone, V.; Mennucci, B.; Petersson, G. A.; Nakatsuji, H.; Caricato, M.; Li, X.; Hratchian, H. P.; Izmaylov, A. F.; Bloino, J.; Zheng, G.; Sonnenberg, J. L.; Hada, M.; Ehara, M.; Toyota, K.; Fukuda, R.; Hasegawa, J.; Ishida, M.; Nakajima, T.; Honda, Y.; Kitao, O.; Nakai, H.; Vreven, T.; Montgomery, Jr., J. A.; Peralta, J. E.; Ogliaro, F.; Bearpark, M.; Heyd, J. J.; Brothers, E.; Kudin, K. N.; Staroverov, V. N.; Keith, T.; Kobayashi, R.; Normand, J.; Raghavachari, K.; Rendell, A.; Burant, J. C.; Iyengar, S. S.; Tomasi, J.; Cossi, M.;

Rega, N.; Millam, J. M.; Klene, M.; Knox, J. E.; Cross, J. B.; Bakken, V.; Adamo, C.; Jaramillo, J.; Gomperts, R.; Stratmann, R. E.; Yazyev, O.; Austin, A. J.; Cammi, R.; Pomelli, C.; Ochterski, J. W.; Martin, R. L.; Morokuma, K.; Zakrzewski, V. G.; Voth, G. A.; Salvador, P.; Dannenberg, J. J.; Dapprich, S.; Daniels, A. D.; Farkas, O.; Foresman, J. B.; Ortiz, J. V.; Cioslowski, J.; Fox, D. J. Gaussian, Inc., Wallingford CT, 2013.

[8] Marenich, S. A. V.; Cramer, C. J.; Truhlar, D. G. Universal Solvation Model Based on Solute Electron Density and on a Continuum Model of the Solvent Defined by the Bulk Dielectric Constant and Atomic Surface Tensions. *J. Phys. Chem. B* **2009**, *113*, 6378−6396.

[9] (a) Hehre, W. J.; Ditchfield, R.; Pople, J. A. Self—Consistent Molecular Orbital Methods. XII. Further Extensions of Gaussian—Type Basis Sets for Use in Molecular Orbital Studies of Organic Molecules. *J. Chem. Phys.* **1972**, *56*, 2257-2261; (b) Hariharan, P. C.; Pople, J. A. The influence of polarization functions on molecular orbital hydrogenation energies. *Theor. Chim. Acta.* **1973**, *28*, 213-222; (c) Francl, M. M.; Pietro, W. J.; Hehre, W. J.; Binkley, J. S.; Gordon, M. S.; DeFrees, D. J.; Pople, J. A. Self-consistent molecular orbital methods. XXIII. A polarization-type basis set for second-row element. *J. Chem. Phys.* **1982**, *77*, 3654–3665.

[10] Andrae, D.; Häussermann, U.; Dolg M.; Stoll, H.; Preuss, H. Energy-adjusted ab initio pseudopotentials for the second and third row transition elements. *Theor. Chim. Acta* **1990**, *77*, 123–141.

[11] Ehlers, A. W.; Boehme, M.; Dapprich, S.; Gobbi, A.; Hoellwarth, A.; Jonas, V.; Koehler, K. F.; Stegmann, R.; Veldkamp, A.; Frenking, G. A set of f-polarization functions for pseudo-potential basis sets of the transition metals Sc-Cu, Y-Ag and La-Au *Chem. Phys. Lett.* **1993**, *208*, 111–114.

[12] (a) Weigend, F.; Ahlrichs, R.; Balanced basis sets of split valence, triple zeta valence and quadruple zeta valence quality for H to Rn: Design and assessment of accuracy. *Phys. Chem. Chem. Phys*. **2005**, *7*, 3297–3305; (b) Weigend, F. Accurate Coulomb-fitting basis sets for H to Rn. *Phys. Chem. Chem. Phys.* **2006**, *8*, 1057-1065.

[13] Grimme, S. Supramolecular binding thermodynamics by dispersion-corrected density functional theory. *Chem. Eur. J.* **2012**, *18*, 9955−9964.

[14] Luchini, G.; Alegre-Requena, J. V.; Funes-Ardoiz, I.; Paton, R. S. GoodVibes: Automated Thermochemistry for Heterogeneous Computational Chemistry Data. *F1000Research*, **2020**, *9*, 291. DOI:10.12688/f1000research.22758.1

[15] Chemcraft - graphical software for visualization of quantum chemistry computations. https://www.chemcraftprog.com

[16] Ahlrichs, R.; Bär, M.; Häser, M.; Horn, H.; Kölmel, C. Electronic structure calculations on workstation computers: The program system turbomole, *Chem. Phys. Lett.* **1989**, *162*, 165–169.

[17] a) Becke, A. D. Density-functional exchange-energy approximation with correct asymptotic behavior. *Phys. Rev. A* **1988**, *38*, 3098–3100; b) Perdew, J. P. Density-functional approximation for the correlation energy of the inhomogeneous electron gas. *Phys. Rev. B* **1986**, *33*, 8822–8824.

[18] Grimme, S.; Antony, J.; Ehrlich, S.; Krieg, H. A consistent and accurate ab initio parametrization of density functional dispersion correction (DFT-D) for the 94 elements H-Pu. *J. Chem. Phys.* **2010**, *132*, 154104–19.

[19] Eichkorn, K.; Treutler, O.; Öhm, H.; Häser, M.; Ahlrichs, R. Auxiliary basis sets to approximate Coulomb potentials. *Chem. Phys. Lett.* **1995**, *242*, 652–660.

[20] See, for instance: a) Fernández, I.; Holzmann, N.; Frenking, G. The valence orbitals of the alkaline-earth atoms. *Chem. Eur. J.* **2020**, *26*, 14194–14210; b) Wang, G.; Noonikara-Poyil, A.; Fernández, I.; Dias, H. V. R. Iron pentacarbonyl ligands on silver scorpionates. *Chem. Commun.* **2022**, *58*, 3222–3225; c) Noonikara-Poyil, A; Ridlen, S. G.; Fernández, I.; Dias, H. V. R. Isolable acetylene complexes of copper and silver. *Chem. Sci.* **2022**, *13*, 7190–7203.

[21] Glendening, E. D.; Landis, C. R.; Weinhold, F. NBO 6.0: Natural bond orbital analysis program. *J. Comput. Chem.* **2013**, *34*, 1429–1437.

[22] a) Morokuma, K. Molecular Orbital Studies of Hydrogen Bonds. III. C=O···H–O Hydrogen Bond in $H_2CO\cdots H_2O$ and $H_2CO\cdots 2H_2O$. *J. Chem. Phys.* **1971**, 55, 1236. b) Ziegler, T; Rauk, A. On the calculation of bonding energies by the Hartree Fock Slater method. *Theor. Chim. Acta* **1977**, 46, 1–10.

[23] Mitoraj, M. P.; Michalak, A.; Ziegler, T. A Combined charge and energy decomposition scheme for bond analysis. *J. Chem. Theory Comput.* **2009**, *5*, 962–975.

[24] a) te Velde, G.; Bickelhaupt, F. M.; Baerends, E. J.; Fonseca Guerra, C.; van Gisbergen, S. J. A.; Snijders, J. G.; Ziegler, T. Chemistry with ADF. *J. Comput. Chem.* **2001**, *22*, 931–967; b) ADF2020, SCM, Theoretical Chemistry, Vrije Universiteit, Amsterdam, The Netherlands, http://www.scm.com.

[25] Snijders, J. G.; Vernooijs, P.; Baerends, E. J. Roothaan-Hartree-Fock-Slater atomic wave functions: Single-zeta, double-zeta, and extended Slater-type basis sets for ^{87}Fr-^{103}Lr. *At. Data Nucl. Data Tables* **1981**, *26*, 483–574.

[26] Krijn, J.; Baerends, E. J. *Fit Functions in the HFS-Method, Internal Report* (in Dutch), Vrije Universiteit Amsterdam, The Netherlands, 1984.

[27] a) van Lenthe, E.; Baerends, E. J.; Snijders, J. G. Relativistic regular two-component Hamiltonians. *J. Chem. Phys.* **1993**, *99*, 4597–4610; b) van Lenthe, E.; Baerends, E. J.; Snijders, J. G. Relativistic total energy using regular approximations. *J. Chem. Phys.* **1994**, *101*, 9783–9792; c) van Lenthe, E.; Ehlers, A.; Baerends, E. J. Geometry optimizations in the zero order regular approximation for relativistic effects. *J. Chem. Phys.* **1999**, *110*, 8943–8953.

[28] Sheldrick, G. M. Crystal structure refinement with SHELXL. *Acta Cryst*. **2008**, *A64*, 112–122.

[29] Dolomanov, O. V.; Bourhis, L. J.; Gildea, R. J.; Howard, J. A. K.; Puschmann, H. OLEX2: a complete structure solution, refinement and analysis program. *J. Appl. Cryst*. **2009**, *42*, 339–341.

[30] van der Sluis, P.; Spek, A. L. BYPASS: an effective method for the refinement of crystal structures containing disordered solvent regions. *Acta Cryst.* **1990**, *A46*, 194–201.
